# Density-mediated freshwater plastisphere microbiomes preferentially degrade conventional rather than biodegradable microplastics

**DOI:** 10.1093/ismejo/wrag167

**Published:** 2026-06-27

**Authors:** Haiyu Zhang, Peng Liu, Yiqi Chen, Jing Lv, Xinrui Zhang, Jiale Zhang, Yifan Sun, Chaoyi Wang, Shichen Wei, Xiaojuan Wang, Shixiang Gao, Xun Qian, Hanzhong Jia, James M Tiedje

**Affiliations:** State Key Laboratory of Soil and Water Conservation and Desertification Control, College of Natural Resources and Environment, Northwest A&F University, Yangling 712100, China; Key Laboratory of Pollution Processes and Environmental Criteria (Ministry of Education), Tianjin Key Laboratory of Environmental Remediation and Pollution Control, College of Environmental Science and Engineering, Nankai University, Tianjin 300350, China; State Key Laboratory of Soil and Water Conservation and Desertification Control, College of Natural Resources and Environment, Northwest A&F University, Yangling 712100, China; State Key Laboratory of Soil and Water Conservation and Desertification Control, College of Natural Resources and Environment, Northwest A&F University, Yangling 712100, China; State Key Laboratory of Soil and Water Conservation and Desertification Control, College of Natural Resources and Environment, Northwest A&F University, Yangling 712100, China; State Key Laboratory of Soil and Water Conservation and Desertification Control, College of Natural Resources and Environment, Northwest A&F University, Yangling 712100, China; State Key Laboratory of Soil and Water Conservation and Desertification Control, College of Natural Resources and Environment, Northwest A&F University, Yangling 712100, China; State Key Laboratory of Soil and Water Conservation and Desertification Control, College of Natural Resources and Environment, Northwest A&F University, Yangling 712100, China; State Key Laboratory of Soil and Water Conservation and Desertification Control, College of Natural Resources and Environment, Northwest A&F University, Yangling 712100, China; State Key Laboratory of Soil and Water Conservation and Desertification Control, College of Natural Resources and Environment, Northwest A&F University, Yangling 712100, China; State Key Laboratory of Soil and Water Conservation and Desertification Control, College of Natural Resources and Environment, Northwest A&F University, Yangling 712100, China; State Key Laboratory of Water Pollution Control and Green Resource Recycling, School of Environment, Nanjing University, Nanjing 210023, China; State Key Laboratory of Soil and Water Conservation and Desertification Control, College of Natural Resources and Environment, Northwest A&F University, Yangling 712100, China; State Key Laboratory of Soil and Water Conservation and Desertification Control, College of Natural Resources and Environment, Northwest A&F University, Yangling 712100, China; State Key Laboratory of Soil and Water Conservation and Desertification Control, College of Natural Resources and Environment, Northwest A&F University, Yangling 712100, China; Center for Microbial Ecology, Michigan State University, East Lansing, MI 48824, United States

**Keywords:** microplastics, photoaging, plastisphere, microbial degradation, multi-omics

## Abstract

The escalating demand for plastics leads to ubiquitous microplastic (MP) pollution worldwide. Existing evidence suggests that biodegradable MPs degrade faster than conventional MPs in aquatic environments. Here, we demonstrate the greater biodegradability of conventional polypropylene (PP) over biodegradable polylactic acid (PLA) in freshwater based on field survey, mesocosm experiment, co-culture assay, and multi-omics analysis. The biodegradation rate is 3.3-fold higher for PP compared to PLA, and this difference is more pronounced between photoaged MPs (5.7-fold). The unexpected superior biodegradability of PP is supported by a greater diversity of MP-degrading bacteria in PP biofilm (predominantly aerobes) than in PLA biofilm (mainly facultative and obligate anaerobes). The inferior biodegradability of PLA is attributed to microbial growth constraints in the plastisphere driven by oxic-to-hypoxic/anoxic transition, oxygen-containing functional group detachment from the polymer, and lactide accumulation during long-term biodegradation. Our findings reveal previously overlooked but important environmental fates and impacts of biodegradable plastics against increasing substitution of conventional plastics with biodegradable alternatives.

## Introduction

Plastic pollution due to massive waste production poses severe threats to terrestrial and aquatic ecosystems, raising global concern. Studies estimate that approximately 12 000 million tons (Mt) of plastic waste will accumulate in the environment by 2050 [1]. Conventional plastics, including polypropylene (PP), polyethylene (PE), and polyethylene glycol terephthalate (PET), comprise the majority of plastic waste and persist for long periods [1]. To tackle plastic pollution, biodegradable plastics are increasingly used as sustainable substitutes for conventional plastics [2]. The global biodegradable plastic market is predicted to grow prominently, with production capacities increasing from 2.2 Mt in 2022 to 6.3 Mt in 2027 [3]. Among biodegradable plastics, polylactic acid (PLA) accounts for the largest proportion of plastic production, followed by polybutylene adipate terephthalate and polyhydroxyalkanoates [4]. The proliferation of biodegradable plastics necessitates close monitoring of their environmental fate and risks relative to conventional counterparts.

Upon entering the environment, plastics break down into fragments of varying sizes through weathering. Microplastics (MPs) are small particles <5 mm in size, which constitute the predominant fraction of plastic waste and pose major threats to environmental health [5, 6]. Recent studies identify freshwater as a critical sink of MPs [7], where >109 Mt have accumulated by 2022 [8]. In aquatic systems, a diver se microbiota colonizes the MP surface, forming biofilms [9]. Various microbial consortia composed of abundant bacteria, fungi, algae, and protozoans show a high ability to biodegrade MPs [10]. The prevailing paradigm posits that compared to conventional MPs containing a stable carbon–carbon backbone, biodegradable MPs exhibit superior biodegradability due to their hydrolyzable chemical bonds and functional groups (e.g. esters, carbonyls, and amides) [11].

This paradigm that biodegradable MPs have inherently higher biodegradability than conventional MPs has major biases deviating from real facts. (i) Unchanged biodegradation pattern of MPs in the full-life degradation period. Actually, the biodegradability of conventional and biodegradable MPs may reversely change under prolonged environmental exposure. Unlike continuous oxidation of conventional MPs, microbial-driven hydrolytic breakdown of biodegradable MPs triggers the detachment of oxygen-containing functional groups in short-term periods, potentially establishing unfavorable niches for long-term biodegradation due to declined hydrophilicity [12]. (ii) Same biodegradation environment for various MPs. In natural environment, the biodegradation environments for various MPs vary in a density-dependent manner. For example, conventional PE and PP with lower densities (0.88–0.96 g/cm^3^) float in surface oxic water [13, 14], whereas biodegradable PLA and polybutylene adipate terephthalate with higher densities (1.18–1.30 g/cm^3^) tend to settle in bottom hypoxic/anoxic water [15, 16]. These distinct environmental conditions lead to the involvement of different MP-degrading microbiota in biodegradation. (iii) Original form of MPs in the biodegradation. However, MPs are subjected to universal photoaging prior to biofilm formation, which suggests the biodegradation of MPs in photoaged forms rather than original forms. Photoaging may invert the biodegradation hierarchy between conventional and biodegradable MPs by triggering physicochemical modification (e.g. oxidizability, hydrophobicity, and crystallinity) [17, 18]. Such modification can modulate the colonization potential of MP-degrading microbiota [19], affecting MP biodegradability. However, current studies mainly focus on laboratory conditions with short period and small scale, which exhibits large deviations from real biodegradation conditions. Therefore, long-term, large-scale, and outdoor biodegradation tests should be conducted to truly uncover the biodegradability of conventional and biodegradable MPs.

The primary objective of this study was to uncover the real biodegradability evolution of conventional and biodegradable MPs before and after photoaging in freshwater. We firstly monitored the biodegradability of actual plastic waste samples based on field survey. Then, we assessed the biodegradation kinetics of conventional and biodegradable MPs using a long-term mesocosm experiment. Through metagenomic and high-throughput sequencing, we identified distinct microbiota and metabolic pathways in MP biofilms related to biodegradation and isolated key MP-degrading strains to perform co-culture assay. By integrating gas chromatography-tandem mass spectrometry (GC–MS/MS) with metabolomic and proteomic analyses, we elucidated differential biodegradation routes and mechanisms for conventional and biodegradable MPs. In contrast to previous findings, our results show that conventional PP possesses an unexpected higher biodegradability than biodegradable PLA under long-term freshwater exposure, and this pattern is more pronounced in photoaged MPs. The differences in MP biodegradability are predominantly attributed to aerobic-to-facultative/obligate anaerobic transition of MP-degrading microbiota in the plastisphere driven by varying density and biodegradation properties of PP and PLA. This study highlights the important environmental impact of biodegradable MPs, underscoring the sustaining assessment on their ecological risks over increasing substitution of conventional plastics with biodegradable alternatives.

## Materials and methods

### Field sampling of plastic waste and preparation of microplastic samples

The detailed information on field sampling of plastic waste and preparation of virgin and photoaged MP samples is provided in Supplementary Text, Figs S1–S3 and Table S1.

### Mesocosm incubation experiment

To simulate the natural biodegradation of MPs, a mesocosm experiment was conducted in 25 L containers (inner diameter 300 mm, height 450 mm) at ~25°C. The experiment consisted of four treatment groups: virgin PP, photoaged PP, virgin PLA, and photoaged PLA (Fig. S4). A 25 g MP sample was added to 20 L of natural freshwater, giving a concentration of 1.25 g/L (92 000 particles/L) [20, 21]. There were three replicates for each treatment group. To evaluate potential abiotic degradation during incubation, control groups were set up in parallel by incubating PP or PLA in natural freshwater after autoclaving (121°C, 0.1 MPa) and HgCl_2_ treatment (Supplementary Text). During the experimental period, water parameters were monitored to assess potential changes in aquatic environments (Supplementary Text). The water pH remained stable, whereas the dissolved oxygen, total organic carbon, total nitrogen, and total phosphorus concentrations trended downward with prolonging incubation time (Fig. S5). To maintain nutrient levels, two-thirds of the incubation water was exchanged with fresh natural water every 60 days (with no extra nutrient addition). A 6-mm inner diameter silicone tube was used to siphon out the incubation water, and natural freshwater was gently added to minimize disturbance of biofilm formation. A stainless-steel sieve (pore size 100 μm) was used to prevent the loss of MPs during water exchange. MPs (2.5 g) and water samples (50 ml) were collected at 0, 60, 120, 180, and 210 days by filtrating through a 0.22 μm glass fiber filter. The collected samples were cleaned with phosphate-buffered saline, freeze-dried, and then stored in sealed containers at −20°C until use.

### Biofilm biomass and microbial community analysis

Biofilm biomass of MPs was quantified using a modified crystal violet staining method [22]. After centrifugation to obtain biofilm suspensions, the protein and polysaccharide contents were determined by the Lowry method and phenol-sulfuric acid assay using a ultraviolet–visible (UV–vis) spectrophotometer (GENESYS 50; Thermo Fisher Scientific, Waltham, USA) at wavelengths of 500 and 490 nm, respectively [23, 24]. MP-colonizing microbiota and extracellular polymeric substance (EPS) were characterized using scanning electron microscopy (SEM; Nova NanoSEM 450; FEI Corp., Hillsboro, USA). The chemical composition of EPS was determined by Fourier transform infrared (FTIR) spectroscopy (Vetex70; Bruker, Ettlingen, Germany). The live and dead cells in biofilm were observed by a confocal laser scanning microscope (FV3000; Olympus, Hachioji, Japan). The detailed methods are described in Supplementary Text. The microbial communities in water samples and MP biofilms were analyzed by high-throughput sequencing. To delve deeper into microbial community compositions and functions of 210 day-MP biofilms were performed by metagenomic sequencing (Supplementary Text).

### Isolation of microplastic-degrading strains and co-culture assay with microplastics

MP-degrading strains in biofilms were screened and enriched using an inorganic salt medium supplemented with 0.5% (*w/v* carbon content) PP or PLA as the sole carbon source (Fig. S4, Table S2) [25]. Detailed operation on isolation of MP-degrading strains was provided in Supplementary Text.

The ability of single strains and microbial consortia for biodegradation of MPs were determined by co-culture assay. There were eight treatment groups: (i) PP and (ii) PLA as blank controls; (iii) PP + PP_strain_ and (iv) PLA + PLA_strain_ to determine the biodegradation ability of single strains; (v) PP + PLA_strain_ and (vi) PLA + PP_strain_ to verify the generality or specificity of single strains; (vii) PP + PP_consortium_, and (viii) PLA + PLA_consortium_ to assess the biodegradation ability of microbial consortia. PP or PLA (0.5% *w/v* carbon content) was added to 150 ml of inorganic salt medium in 250 mL culture flasks, followed by inoculation (6 × 10^8^ cells/ml). All cultures were incubated in a sterile oscillator at 30°C and 150 rpm for 45 days. One-half of the medium was exchanged every 20 days to maintain nutrient supply. Bacterial growth was monitored by measuring the optical density of bacterial cultures at 600 nm (OD_600_) of the medium every 2 days using a UV–vis spectrophotometer (GENESYS 50; Thermo Fisher Scientific). Culture broth and MP samples were collected at 0, 20, and 45 days. The culture samples were directly stored at −20°C and the MP samples were freeze-dried and then stored at −20°C until use.

### Characterization of microplastic biodegradation properties and products

As for mesocosm and co-culture tests, the biofilms in MPs were removed using an ultrasonic-assisted ethanol lysis method (Supplementary Text), which achieved a removal efficiency of 70.0–100.0% (Fig. S6). The weight was determined using an electronic balance (BSA124S; Sartorius, Göttingen, Germany), and weight loss (%) was calculated *via* the formula: 100.0% × [(initial weight – final weight)/initial weight]. The morphological changes were characterized by SEM (Nova NanoSEM 450; FEI Corp.). The size distributions were quantified by enumerating fragments in SEM images using the Nano Measurer software (v1.2.5; Fudan University, Shanghai, China) [26, 27]. The specific surface areas were measured by using an automated surface area analyzer (ASAP 2460; Micromeritics, Norcross, USA). The chemical properties were examined using FTIR (Vetex70; Bruker) and X-ray photoelectron spectroscopy (Nexsa; Thermo Fisher Scientific). The thermal stability was evaluated by thermogravimetric analysis (TGA 4000; PerkinElmer, Waltham, USA). The molecular weight distribution was determined using gel permeation chromatography (1260 Infinity II; Agilent Technologies, Santa Clara, USA) with tetrahydrofuran as the mobile phase for PLA, and high-temperature gel permeation chromatography (PL-GPC 220; Agilent Technologies) with trichlorobenzene as the mobile phase for PP. The carbon content was measured by an organic elemental analyzer (vario MACRO cube; Elementar, Hanau, Germany). Water contact angle measurements of co-cultured MP samples, to assess microbial degradation-induced hydrophobicity changes, were performed using an optical contact angle meter (SZ-CAMC32; Sunzern, Shanghai, China). To reveal the biodegradation routes, the biodegradation products were detected by GC–MS/MS (Trace 1610-TSQ 9610; Thermo Fisher Scientific). To quantitatively compare the biodegradability between PP and PLA, the integrated biodegradation rate (IBR) was calculated by integrating multiple biodegradation properties. Details on these analysis methods were described in Supplementary Text.

### Determination of fluorescein diacetate hydrolase activity and reactive oxygen species contents

Fluorescein diacetate (FDA) hydrolase activity in the co-culture medium of MPs and strains was assayed using commercial kits (Solarbio Science, Beijing, China). The contents of intracellular/extracellular superoxide anion (O_2_^•–^) and hydrogen peroxide (H_2_O_2_) were determined using reactive oxygen species (ROS) detection kits (Solarbio Science) according to the manufacturer’s instructions. Detailed protocols were provided in Supplementary Text.

### Multi-omics analysis of microplastic-degrading strains

Whole-genome sequencing was performed to identify PP_strain_ and PLA_strain_ and elucidate their biodegradation mechanisms for PP and PLA. Genomic DNA was extracted from strain cultures using the TIANamp Bacteria DNA Kit (Tiangen Biotech). The extracted DNA was then split into two fetches and sequenced using an Illumina second-generation sequencing platform (Biomarker Technologies) with a PE150 strategy. Species annotation was carried out using the genome taxonomy database (https://gtdb.ecogenomic.org/), and functional annotation was achieved using the Kyoto Encyclopedia of Genes and Genomes (KEGG; https://www.genome.jp/kegg/) and gene ontology databases (https://www.geneontology.org/) (Supplementary Text).

Metabolomic and proteomic analyses were performed to detect the metabolites and proteins of PP and PLA produced during biodegradation. Metabolomic analysis was conducted using an Acquity I-Class PLUS UPLC system (Waters Corporation, Milford, USA) coupled with a Waters Xevo G2-XS QT high-resolution mass spectrometer (Waters Corporation), with a Waters Acquity UPLC HSS T3 column (1.8 μm, 2.1 × 100 mm). The raw data collected with MassLynx V4.2 were processed using the Progenesis QI software for peak extraction, alignment, and identification [28]. The metabolites were further analyzed for pathway information using the KEGG database (https://www.genome.jp/kegg/), Human Metabolome database (https://hmdb.ca/), and LIPID MAPS database (https://lipidmaps.org/). Shotgun proteomic analyses were performed using a U3000 UHPLC system (Thermo Fisher Scientific) coupled with an Orbitrap Fusion mass spectrometer (Thermo Fisher Scientific) in the data-dependent acquisition mode [29]. MS2-based LFQ was carried out by analyzing DIA raw data using the Biognosys Spectronaut (v13) software with dynamic data extraction and a q-value cutoff of 0.01 [30]. Gene ontology (https://www.geneontology.org) and InterPro (https://www.ebi.ac.uk/interpro/) analyses were conducted using the InterProScan-5 program (https://www.ebi.ac.uk/jdispatcher/pfa/iprscan5) against the non-redundant protein database. Clusters of Orthologous Groups (COG) and KEGG were used to analyze protein families and pathways [31]. Detailed protocols were provided in Supplementary Text.

### Statistical analysis

Microbial *α*-diversity was calculated and displayed by the QIIME2 and R software, respectively [32]. *β*-diversity was determined using QIIME. One-way analysis of variance followed by Tukey’s test was performed using GraphPad Prism (v9.5.0; https://www.graphpad.com/). Spearman correlation coefficients (*ρ*) among amplicon sequence variants (ASVs) were computed using the vegan package in R (v4.3.1; https://cran.r-project.org/package=vegan). Gephi (v0.10.1; https://gephi.org/) was used to visualize the co-occurrence networks of biofilm bacteria. Partial least squares structural equation modeling (PLS-SEM) was performed using the SmartPLS 4 software [33]. Detailed analysis methods were described in Supplementary Text.

## Results

### Field survey of plastic waste biodegradation in freshwater

**Figure 1:**
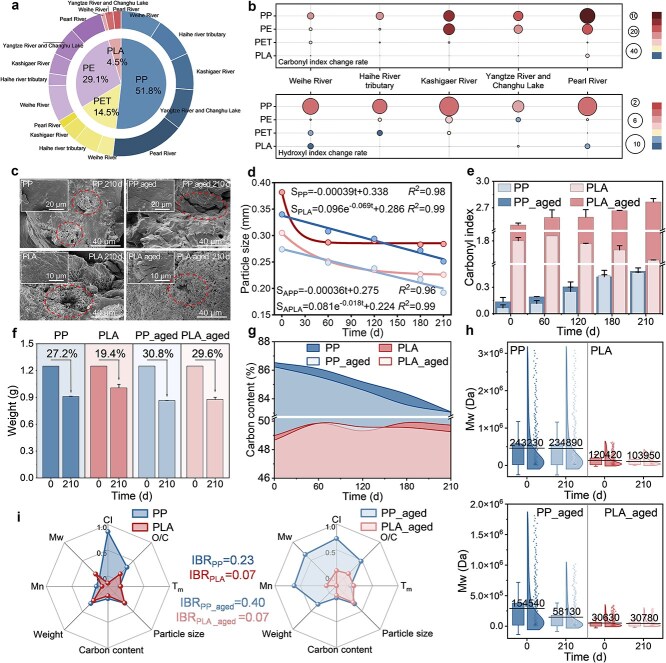
Field survey of plastic waste biodegradation and mesocosm assessment of MP biodegradability in freshwater. (a) Types of field-collected plastic waste from six important river systems in China (*n* = 111): Yangtze River, Pearl River, Haihe River, Weihe River, Kashigaer River, and Changhu Lake. (b) Surface oxidation of field-collected plastic waste denoted by hydroxyl index and carbonyl index change rate relative to original new sample. (c) Surface morphology of conventional PP and biodegradable PLA after 210 days of mesocosm incubation. “PP_210 d”, “PP_aged_210 d”, “PLA_210 d”, and “PLA_aged_210 d” denote virgin PP, photoaged PP, virgin PLA, and photoaged PLA, respectively. (d) Particle size, (e) carbonyl index, (f) weight, (g) carbon content, and (h) Mw of PP and PLA during mesocosm incubation. (i) Star plots showing the IBR value of MPs based on their weight, carbon content, particle size, and T_m_, carbonyl index, O/C ratio, Mw, and Mn.

More than 100 types of plastic waste, including bags, bottles, straws, films, boxes, lids, scoops, cups, and bowls were retrieved from 31 freshwater sampling sites in five different major regions of China (Figs S1 and S2). Among the plastic samples, PP was the most frequently identified conventional plastic by FTIR spectroscopy (51.8%), followed by PE (29.1%) and PET (14.5%) (Fig. 1a). The frequent detection of PP in freshwater is related to its common use in daily life [34]. Despite the low detection frequency of PLA (4.5%), the result suggests biodegradable plastic pollution [35]. SEM revealed the presence of diverse microbial morphotypes, primarily filamentous, spherical, and rod-shaped structures on plastic surfaces (Fig. S7). Biofilm formation analysis revealed different levels of biofilm accumulation across all samples. The highest biomass values were observed for PP bowl_1 and PE bag_2 in Kashigaer River and PP bag_5 in Weihe River. In contrast, PLA box_1 in Yangtze River, together with PE bag_1 and PLA straw_2 in Pearl River, yielded the lowest biomass values (Fig. S8). The differences in biofilm biomass among plastic samples are likely due to their distinct physicochemical properties and exposure periods in the environment [36].

After biofilm removal, bioerosion occurred in some plastic samples (e.g. PP bag, PE film, and PLA box in various river systems), as indicated by the formation of defects, holes, and fine cracks (see elliptical annotation in Fig. S9). Also, new oxygen-containing functional groups (e.g. C=O and H–O) were detected in biofilm-removed samples, reflecting oxidative modification mediated by biofilm microbiota (Fig. S10). Compared to their virgin counterparts, PP and PE plastics exhibited maximum increases of 32.8- and 5.3-fold, respectively, in the hydroxyl index (characterizing the oxidation degree of plastics). Conversely, the hydroxyl index of most PLA plastics decreased by a maximum of 16.2-fold (Fig. S11). A similar case occurred in the carbonyl index, with a greater increase in PP than PLA (Fig. S12). These results imply enhanced oxidation of PP plastics and diminished oxidation of PLA plastics in the field (Fig. 1b). This disparity underscores potentially differential responses of conventional and biodegradable plastics to microbial degradation in aquatic environments.

### Mesocosm assessment of polypropylene and polylactic acid biodegradability dynamic

In view of the high detection frequency of PP and its similar biofilm formation and biodegradation profiles with PE, we conducted a mesocosm experiment with PP and PLA. These two types of MPs were used as representative samples to characterize their biodegradation dynamics in freshwater (Figs S4 and S13). After 60 days of incubation, small pits appeared on both MP types, which evolved into larger holes and cracks at 210 days (Fig. 1c, Fig. S14). Additionally, plastic fragmentation occurred during incubation, where PP exhibited a slightly greater reduction in particle size over 210 days compared to PLA (26.5% versus 26.3%; Table S3). The particle size of PP followed a linear regression model, steadily decreasing throughout the incubation period (*R*^2^ = 0.98). The particle size of PLA followed an exponential model, rapidly decreasing within 30 days and then leveling off (*R*^2^ = 0.99, Fig. 1d). Distinct fragmentation profiles were also observed for photoaged PP versus PLA, possibly governed by their inherent physicochemical properties, such as oxygen-containing functional groups (as discussed in the last section) [37]. Further, particle size distribution analysis revealed differences in the fragmentation process between PP and PLA. In both cases, MPs were dominated by ≥0.3 mm particles (>82.0%), mainly distributed in the size range of 0.3–0.4 mm. There was a progressive shift in the particle size of PP from large (0.3–0.4 mm) to small (0.2–0.3 mm) during incubation. However, PLA experienced a sharp decrease in particle size exclusively within 60 days (−46.9%) and remained stable afterwards (−8.9%; Fig. S15). These results establish that, unlike PP showing a relatively stable fragmentation pattern, PLA develops evolving recalcitrance, rather than being continuously fragmented, during prolonged biodegradation.

FTIR spectra indicated the formation of oxygen-containing functional groups (C–O/C=O and –OH) in PP, a clear sign of oxidation during incubation. In contrast, PLA exhibited minimal changes in functional groups (Fig. S16). The carbonyl index of PP progressively increased by 7.15-fold over the 210-day incubation period (Fig. 1e), suggesting continuous oxidation. As for PLA, its carbonyl index showed a transient rise followed by a precipitous decline (Fig. 1e), implying hydrolysis and cleavage of ester bonds [38]. These results were validated by X-ray photoelectron spectroscopy (Fig. S17). PP showed a greater increase in the oxygen-to-carbon (O/C) ratio (2.4-fold) compared to PLA (1.2-fold) over 210 days (Fig. S18, Table S4), indicating its enhanced oxidation capacity and supporting the field survey results. The abiotic control samples of PP and PLA showed minor changes in surface morphology, particle size, and oxidation capacity throughout the entire incubation period (Fig. S19, Table S5). This indicates the low effects of abiotic factors on surface alterations in MPs. Thus, the surface alterations observed in the mesocosm experiment were mainly caused by biofilm microbiota (detailed descriptions are provided in Supplementary Text).

PP and PLA underwent weight loss, carbon depletion, and chain scission during 210-day incubation, despite different biodegradation degrees. The weight loss of PP reached 27.2%, higher than 19.4% of PLA (Fig. 1f). Progressive carbon depletion was observed in PP, whereas PLA showed a modest elevation in the carbon content within 60 days, followed by a plateau (Fig. 1g). A similar pattern emerged in photoaged MPs, where PP exhibited greater weight loss (30.8%) and carbon depletion (4.0%) compared to PLA (29.6% and 1.5%; Fig. 1f and g). Regarding plastic backbone stability, thermogravimetric analysis revealed a single-stage degradation profile in both MP types. The pyrolysis temperature (T_m_) of PP decreased from 468°C to 455°C after incubation (Fig. S20), suggesting thermal alterations induced by microbial degradation [39]. There was a greater decrease in the T_m_ of PLA (382°C to 364°C) over 210 days (Fig. S20). This implies more rapid breakage of high-molecular-weight fractions in PLA [40], which was confirmed by gel permeation chromatography. Both MP types exhibited a reduction in weight-average molecular weight (Mw) after incubation (Fig. 1h). The reduction rate of Mw in PP was lower than that of PLA, evidencing more extensive cleavage of high-molecular-weight fractions in biodegradable MPs [41]. However, the opposite case occurred for number-average molecular weight (Mn), which decreased in PP but increased in PLA after incubation (Fig. S21). These results point to that unlike random chain scission in PP, the high-molecular-weight fractions of PLA rapidly break down to a certain molecular range and subsequently become relatively stable. Consistently, a greater decrease occurred in the polydispersity index of PLA (20.8%) than PP (1.1%) over 210 days (Table S6). Compared to the virgin counterparts, photoaged MPs showed more pronounced chain scission, with PP undergoing particularly significant alterations relative to PLA (Table S6). This demonstrates that photoaging improves the biodegradability of PP whereas heightening the relative stability of PLA in freshwater.

To quantitatively compare the biodegradability between PP and PLA, an IBR value was calculated by considering weight, carbon content, molecular weight, carbonyl index, O/C ratio, particle size, and T_m_. The IBR value of PP (0.23) was 3.3-fold higher than that of PLA (0.07; Fig. 1i), indicating its greater biodegradability. Photoaging enhanced the difference in the IBR value between various MPs, with a 5.7-fold increase in PP (0.40) compared to PLA (0.07; Fig. 1i). Many researchers hold that biodegradable plastics, including MPs, break down more readily than their conventional counterparts in the environment [42, 43]. Our study provides new evidence that biodegradable MPs (e.g. PLA) do not necessarily degrade faster than conventional MPs (e.g. PP) under long-term freshwater exposure, especially after photoaging. One possible explanation for this discrepancy is that previous studies mainly looked at the biodegradation of MPs within short incubation periods [44, 45]. Indeed, biodegradable MPs are more readily biodegraded than conventional MPs during short-term incubation (<60 days), which was corroborated by our study. However, the biodegradation process of biodegradable MPs (PLA) slows down over a longer exposure period compared to that of conventional MPs (PP).

### Microbial community structure and function in microplastic biofilms related to biodegradation

**Figure 2:**
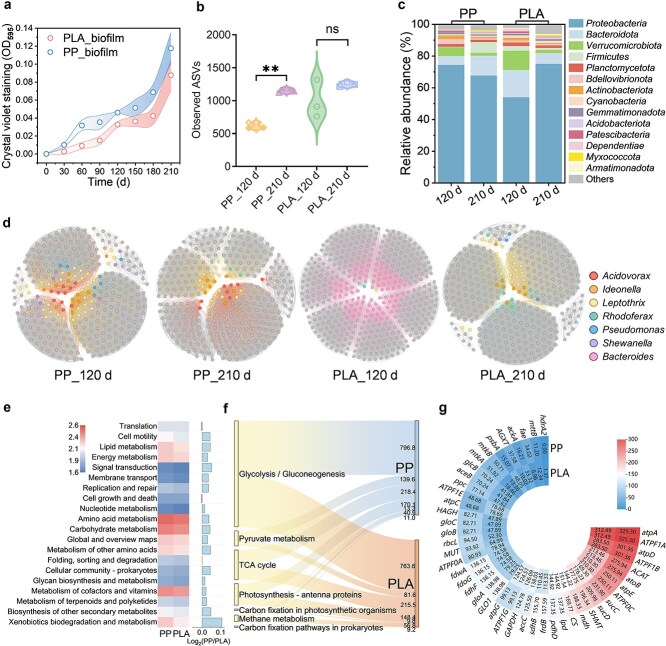
Taxonomic and functional profiling of biofilm microbiota in PP and PLA. (a) Biofilm biomass as a function of incubation time. (b) Microbial *α*-diversity based on observed ASVs. ^**^P < .01; ns means not significant. (c) Microbial community compositions at the phylum level. (d) Co-occurrence networks based on ASVs data. The nodes representing MP-degrading bacteria and none MP-degrading bacteria, respectively were identified by comparing sequencing data with PlasticDB. (e) Functional enrichment of KEGG level 2 pathways and their fold change differences between PP and PLA biofilms. In the bar chart, pathways upregulated in PP and PLA biofilm are shown with positive and negative fold changes respectively. (f) Abundance of carbon cycle pathways. (g) Roles of various gene families in biodegradation.

SEM images showed the presence of heterogeneous microbial assemblages, including filamentous, bacillary bacteria, and ellipsoid diatoms in incubated MPs (Fig. S22). Biofilm quantification revealed sustained biomass accumulation in MPs over time (Fig. 2a). However, relative to accumulation of EPS (polysaccharides and protein) in PP, PLA underwent EPS depletion after 180 days (Fig. S23), a possible result of reduced microbial activity identified by living microbiota diminishment in PLA (Fig. S24). This phenomenon was in line with the biodegradability pattern between PP and PLA (Fig. 1). Further, photoaging contributed to increased biofilm biomass (1.0–1.2-fold) and EPS content (1.8–8.5-fold) (Figs S23 and S25), indicating the preference of microbiota toward photoaged MPs. In addition, we observed a significantly positive linear correlation between carbonyl index and biofilm biomass for PP (*P* < .05), but a negative correlation for PLA (*P* < .05; Fig. S26). The results hint at the importance of MP-colonizing microbiota in promoting the oxidative degradation of PP and facilitating the cleavage of ester bonds in PLA.

Given that photoaged MPs exhibited biofilm formation and biodegradation patterns similar to those of virgin MPs, microbial profiling based on high-throughput sequencing and metagenomic sequencing was carried out for virgin PP and PLA after mesocosm incubation. *α*-Diversity analysis revealed a higher microbial community diversity in MP biofilms than in surrounding water (Fig. S27). Additionally, the observed ASVs were significantly lower in PP biofilm than in PLA biofilm at 120 days, followed by a sharp increase in PP biofilm and minimal change in PLA biofilm at 210 days (Fig. 2b). These diversity patterns are consistent with the biodegradation dynamics of MPs (Fig. 1). The distinct microbial communities within biofilms were identified between PP and PLA (Fig. S28). The relative abundance of Proteobacteria decreased in PP biofilm but increased in PLA biofilm from 120 to 210 days of incubation, which contrasted with trends in *Firmicutes* and *Bacteroidota* (Fig. 2c). Comparing full-length 16S rRNA gene sequencing with PlasticDB revealed that the MP-degrading bacteria in PP biofilms were dominated by aerobes, such as *Pseudomonas, Ideonella*, and *Leptothrix*. Those in PLA biofilm primarily consisted of facultative anaerobes (e.g. *Rhodoferax*) and obligate anaerobes (e.g. *Bacteroides*) (Fig. 2d). Additionally, the diversity and abundance of MP-degrading bacteria in PP biofilm were higher than that in PLA biofilm at 120 and 210 days, supporting the superior biodegradability of PP compared to PLA.

KEGG analysis identified distinct metabolic pathways between PP and PLA biofilms. At KEGG level 2, PP biofilm exhibited greater enrichment of amino acid metabolism, carbohydrate metabolism, cofactor and vitamin metabolism, and lipid metabolism pathways compared to PLA biofilm (Fig. 2e). This reflects higher metabolic capacity of microbiota in PP biofilm, which could drive continuous biodegradation of PP substrates. In particular, the xenobiotic biodegradation and metabolism pathway was more prominently enriched in PP biofilm than in PLA biofilm. Considering the xenobiotic status of MPs and their metabolic intermediates [46], the observed functional enrichment signifies the greater potential of biofilm microbiota for biodegradation of PP compared to PLA. KEGG level 3 analysis demonstrated enrichment of glycolysis/gluconeogenesis flux and tricarboxylic acid (TCA) cycle in PP biofilm (Fig. 2f). Key energy metabolism genes (*ACAT, AGXT, ATPF0C, ATPF1A*, and *ATPF1B*) were upregulated in PP biofilm (Fig. 2g), which implies enhanced aerobic catabolic pathways providing ample energy for biodegradation. Compared to PP biofilm, PLA biofilm showed an elevated level of anaerobic metabolism, particularly methanogenic pathway. Upregulation of methanogenesis-related genes (*hdrA2, mttB*, and *ackA*) was observed in PLA biofilm, indicating a transition to facultative anaerobe/anaerobe-dominated metabolic regimes (Fig. 2g). This phenomenon stems from the fact that once interfacial tension is breached, PLA sinks to deeper hypoxic/anoxic zones owing to its high density (1.25–1.28 g/cm^3^) [15], thereby imposing selective pressure on attached microbial communities.

### Isolation of microplastic-degrading strains and assessment of their biodegradation ability

**Figure 3:**
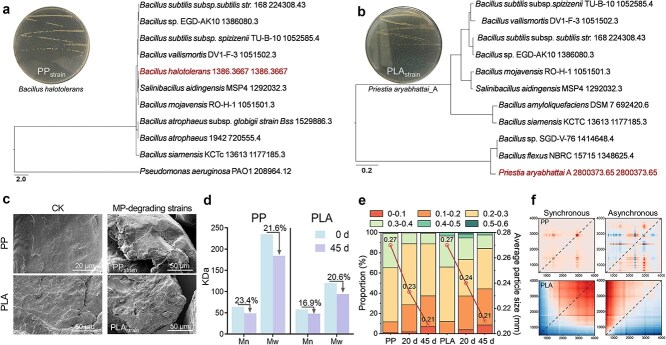
Identification and biodegradation ability of MP-degrading strains isolated from PP and PLA biofilms. Digital photographs and phylogenetic trees of (a) PP_strain_ and (b) PLA_strain_. (c) Surface morphology, (d) molecular weight, (e) particle size, and (f) reaction orders of chemical bonds of PP and PLA after co-culture with strains. CK represents the control with no strains.

Two representative strains that achieved outstanding growth performance in MP biofilms were isolated, designated PP_strain_ and PLA_strain_. Based on phylogenetic analysis, PP_strain_ was classified as *Bacillus halotolerans* (*Bacteria*; *Firmicutes*; *Bacilli*; *Bacillales*; *Bacillaceae*; *Bacillus*) (Fig. 3a), and PLA_strain_ was taxonomically designated as *Priestia aryabhattai*_A (*Bacteria*; *Firmicutes*; *Bacilli*; *Bacillales*; *Bacillaceae*_H; *Priestia*) (Fig. 3b). *B. halotolerans* is an aerobic and halotolerant bacterium that plays a role in plant growth promotion and phytopathogen biocontrol [47]. *P. aryabhattai*_A is a facultative anaerobic and salt-tolerant bacterium that can facilitate plant growth [48]. This study identified *B. halotolerans* and *P. aryabhattai*_A as degraders of MPs by mining the PlasticDB and comparing with known plastic-degrading strains (Tables S7 and S8) [49].

To verify the ability of the isolated strains and microbial consortia for biodegradation of conventional and biodegradable MPs, co-culture assay was conducted with PP and PLA (Fig. S4). Measurements of the OD_600_ revealed sustained cell growth across all eight treatments over a 45-day period (Fig. S29), confirming the favorable activity of single strains and consortia. SEM images showed extensive colonization of bacterial assemblages on PP and PLA substrates (Fig. S30), indicating that both MPs provided favorable surfaces for microbial attachment. After removing the colonized strains and consortia, surface pits and micropores emerged in both PP and PLA (Fig. 3c, Fig. S31). The molecular weight (Mn and Mw) and particle size of PP and PLA both decreased after biodegradation (Fig. 3d and e), suggesting chain scission and fragmentation of both MPs by single strains. Contact angle measurements showed enhanced hydrophilicity in PP after biodegradation by PP_strain_, whereas PLA treated with PLA_strain_ became more hydrophobic (Fig. S32). This observation was supported by FTIR spectra showing enhanced C=O stretching in PP and slight attenuation of H–O bond in PLA after co-culture with the respective single strains (Fig. S33), which suggests different biodegradation processes of PP and PLA. Two-dimensional correlation spectroscopy revealed different reaction orders of chemical bonds in conventional and biodegradable MPs during strain-driven biodegradation (Fig. 3f). For PP, the chemical bonds were prioritized as H–O > –CH > C–O > C=O > C–OH based on synchronous and asynchronous data (Table S9). This indicates that hydroxylation was the dominant oxidation pathway in PP, followed by formation of carbonyl compounds. In the case of PLA, the priority ranking of chemical bonds was –CH > H–O > C=O > –C–OH > –CH–OH > –CH_2_–OH (Table S10). Accordingly, ester bond hydrolysis was the primary pathway in PLA to release lactate derivatives, followed by generation of alcohol and carboxyl groups. Further, cross-culture assays showed a smaller reduction in the particle size of PP (3.7%) treated with PLA_strain_ compared to PLA (7.4%) treated with PP_strain_ (Table S11). This indicates that PP_strain_ and PLA_strain_ possessed biodegradation ability, despite of the superior ability of PP_strain_.

### Divergent metabolic strategies and genetic adaptations of microplastic-degrading strains

**Figure 4:**
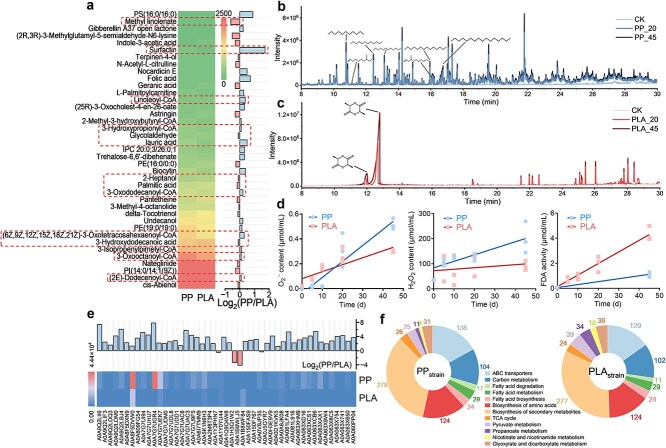
Key metabolites and biomarkers involved in biodegradation of PP and PLA by the isolated strains. (a) Heatmap of key metabolite abundance and fold change differences. In the bar chart, upregulated metabolites are shown with positive and negative fold changes for PP and PLA, respectively. Biodegradation products from (b) PP and (c) PLA by GC–MS/MS. CK represents the control with no strains. (d) Changes in O_2_^•–^ and H_2_O_2_ contents and FDA hydrolase activity. (e) Heatmap of protein abundance and fold change differences. In the bar chart, upregulated proteins are shown with positive and negative fold changes for PP andPLA, respectively. (f) Classification of functional genes annotated in PP_strain_ and PLA_strain_.

The major types of chain-scission intermediates and metabolites from MP biodegradation by PP_strain_ and PLA_strain_ were identified using metabolomic and GC–MS/MS analyses. With regard to PP, upregulation of surfactin production was observed (Fig. 4a), indicating an adaptive response facilitating bacterial attachment onto hydrophobic surfaces [50, 51]. Detection of long-chain alkanes (undecane, tetradecane, and hexadecane) and *α*-olefins (1-undecene, 4-methyl, 1-decene, and 8-methyl) substantiated carbon–carbon backbone scission and oxidation of PP (Fig. 4b). Furthermore, hydroxylated alkane derivatives (undecanol and 2-heptanol) were identified in PP extracts, reflecting oxidative hydroxylation. Fatty acid intermediates (lauric and palmitic) and acyl-coenzyme A (acyl-CoA) derivatives (3-oxooctanoyl-CoA and 3-oxododecanoyl-CoA) were also detected (Fig. 4a), indicating the activation of fatty-acid *β*-oxidation pathways for PP biodegradation [52]. Upregulation of fatty acid metabolism (linoleoyl-CoA, (6Z,9Z,12Z,15Z,18Z,21Z)-3-oxotetracosahexaenoyl-CoA) and degradation (3-oxododecanoyl-CoA) corroborated the biodegradation profile of PP (Fig. 4a). As for PLA, lactide was predominantly accumulated compared to other intermediates (Fig. 4c), suggesting ester bond hydrolysis as the primary depolymerization mechanism [53]. Hydrolysis was further evidenced by a continuous increase in FDA hydrolase activity over time (Fig. 4d), aligning with microbial-driven ester bond cleavage. Additionally, lactate oligomers, hydroxy aldehydes (glycolaldehyde), and hydroxy fatty acids (3-hydroxydecanoic acid and 3-hydroxy-dodecanoic acid) were detected in PLA extracts, providing evidence for subsequent oxidation cascades after ester bond hydrolysis (Fig. 4a). Identification of other key metabolites (3-hydroxypropionyl-CoA, (2E)-dodecenoyl-CoA, and 2-methyl-3-hydroxybutyryl-CoA) highlighted the crucial role of fatty acid degradation in PLA_strain_ metabolism (Fig. 4a).

Proteomic analysis of PP_strain_ identified key enzymes such as superoxide dismutase (SOD; A0A1Y3PHN7) and catalase (A0A0V8JP35; Fig. 4e). Since these enzymes are responsible for detoxifying ROS, the result implies ROS production during co-culture [54]. Consistently, ROS measurements showed that the contents of O_2_^•–^ and H_2_O_2_ in PP extracts trended upward over time (Fig. 4d). Additionally, a ferritin-like domain protein (A0A5D4NB62) was detected, indicating the production of hydroxyl radical (•OH) [55]. These ROS can actively attack C–H bond in PP to enable oxidation, although attack on microbial cells remains potential concurrence [56, 57]. Furthermore, detection of aldehyde/ketone dehydrogenase (A0A1B8WLJ5) suggested transformation of oxidation products into fatty acids, followed by metabolism for energy production. As for PLA_strain_, low O_2_^•–^ and H_2_O_2_ contents in PLA extracts indicated a minor role of ROS production in biodegradation (Fig. 4d). However, pyruvate dehydrogenase (A0A7G7U9F5) was detected, indicating oxidation of hydrolytic intermediates into acetyl-CoA. Aldehyde dehydrogenase (A0A0J5GYR7) capable of oxidizing aldehydic termini in hydrolytic oligomers to carboxylic acids was also identified (Fig. 4e), which could initiate the fatty acid degradation pathway.

Whole-genome sequencing of PP_strain_ and PLA_strain_ revealed diverse gene clusters encoding key degradation enzymes, supporting their functional specialization in MP biodegradation (Fig. 4f). PP_strain_ harbored three oxygenase and hydrocarbon catabolic genes that encode cytochrome P450/NADPH-cytochrome P450 reductase (EC 1.14.14.1), 2-oxoglutarate dehydrogenase (EC 1.2.4.2), and succinate dehydrogenase/fumarate reductase (EC 1.3.5.1) (Table S12). All of these genes are linked to O_2_^•–^ production through redox cycling [58]. Additionally, the fatty acid degradation pathway contained five key genes encoding aldehyde dehydrogenase (EC 1.2.1.3; Table S12), which can break down PP into metabolizable components and feed them into central metabolic pathways, including the TCA cycle [59]. PLA_strain_ carried five genes encoding carboxylesterase (EC 3.1.1.1) and serine protease (EC 3.4.21.107; Table S12), which are essential for ester hydrolysis to release oligomers [60]. Moreover, there were seven genes encoding aldehyde dehydrogenase (EC 1.2.1.3), lactate dehydrogenase (EC 1.1.1.27), and pyruvate dehydrogenase (EC 1.2.4.1; Table S12), supporting the oxidative metabolic potential of PLA_strain_. The acetyltransferase-acetate kinase pathway comprised two genes for acetyltransferase (EC 2.3.1.8) and acetate kinase (EC 2.7.2.1; Table S12), representing a typical anaerobic reaction in microbial metabolism [61]. This pathway mediates the reversible conversion of acetate, CoA, and adenosine triphosphate (ATP) into acetyl-CoA, adenosine diphosphate (ADP), and inorganic phosphate, establishing dynamic regulation of acetyl-CoA flux [62]. The divergent metabolic strategies between PP_strain_ and PLA_strain_ mirror their specialized adaptations to distinct MP structures. PP_strain_ achieves biodegradation mainly through oxidation pathways, whereas PLA_strain_ adopts hydrolysis and oxidation pathways.

### Differential biodegradation routes and mechanisms for polypropylene and polylactic acid

**Figure 5:**
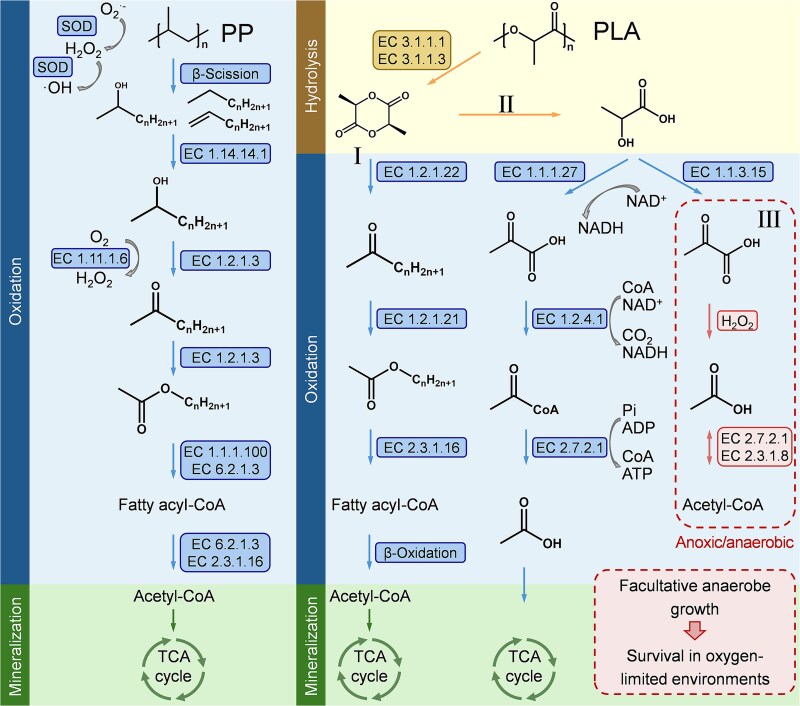
Biodegradation routes of PP and PLA. The biodegradation routes were proposed based on multi-omics analyses integrating GC–MS/MS, metabolomics, proteomics, and whole-genome sequencing data. NADH, reduced nicotinamide adenine dinucleotide; Pi, inorganic phosphate.

The biodegradation of PP involved multi-step biochemical reactions induced by the secretion of surfactin (Fig. 5), a surface-active metabolite facilitating bacterial adhesion and biofilm formation on hydrophobic substrates [50, 51]. Once colonization was established, bacterial metabolic processes enabled sustained production of O_2_^•–^. SOD (EC 1.15.1.1) catalyzed the dismutation of O_2_^•–^ into H_2_O_2_ and then •OH *via* Fenton-mediated cascades [63]. Then, the generated ROS with high redox potential induced substantial oxidation (C–O and –OH formations) and destruction of carbon–carbon backbone into long-chain alkanes (C_10_–C_16_) [56, 57]. Subsequently, long-chain alkanes underwent hydroxylation mediated by cytochrome P450/NADPH-cytochrome P450 reductase (EC 1.14.14.1). Alcohol dehydrogenase (EC 1.2.1.3) catalyzed the oxidation of long-chain hydroxylated alkanes into aldehydes and ketones, with H_2_O_2_ as a byproduct [64]. The catalase (EC 1.11.1.6) decomposed H_2_O_2_ into H_2_O and O_2_, decreasing ROS toxicity and preserving cellular viability. Thereafter, aldehyde dehydrogenase (EC 1.2.1.3) catalyzed the oxidation of aldehydes into fatty acids, which were then activated as acyl-CoA derivatives by long-chain acyl-CoA synthetase (EC 6.2.1.3). Finally, acyl-CoA derivatives were channeled into the *β*-oxidation pathway, yielding two acetyl-CoA molecules through acetyl-CoA acyltransferase (EC 2.3.1.16). This step progressively shortened carbon chains and produced acetyl-CoA to participate in the TCA cycle.

During biodegradation of PLA, carboxylesterase (EC 3.1.1.1) and triacylglycerol lipase (EC 3.1.1.3) catalyzed hydrolytic depolymerization of polyester backbone into lactide monomers and oligomers (Fig. 5). These intermediates participated in several metabolic pathways. In the first oxidation pathway, lactaldehyde dehydrogenase (EC 1.2.1.22) catalyzed the conversion of oligomers (lactide) into hydroxylated aldehydes, which were subsequently oxidized into hydroxylated fatty acids by lactaldehyde dehydrogenase/glycolaldehyde dehydrogenase (EC 1.2.1.21). Then, the hydroxylated fatty acids were activated as acyl-CoA derivatives and ultimately channeled into the *β*-oxidation and TCA cycle. In an additional oxidation pathway, hydrolysis of lactide generated lactate, which was oxidized to pyruvate by lactate dehydrogenase (EC 1.1.1.27) or glycolate oxidase (EC 1.1.3.15). Nicotinamide adenine dinucleotide (NAD^+^) synthetase (EC 6.3.1.5) facilitated sustained NAD^+^ generation to maintain redox balance [65]. Pyruvate was subsequently converted to acetyl-CoA by pyruvate dehydrogenase (EC 1.2.4.1). Finally, acetate kinase (EC 2.7.2.1) catalyzed the conversion of acetyl-CoA to acetate coupled with ATP production, thereby facilitating microbial growth [66]. Under anoxic conditions, pyruvate bypassed acetyl-CoA biosynthesis and was oxidized to acetate *via* H_2_O_2_-mediated reactions [67]. Then, acetate underwent reversible conversion to acetyl-CoA through acetyltransferase-acetate kinase pathway (EC 2.3.1.8/EC 2.7.2.1), a critical mechanism regulating acetyl-CoA flux [62]. This adaptation underscores facultative anaerobic growth of PLA_strain_, allowing for survival in oxygen-limited environments.

## Discussion

**Figure 6:**
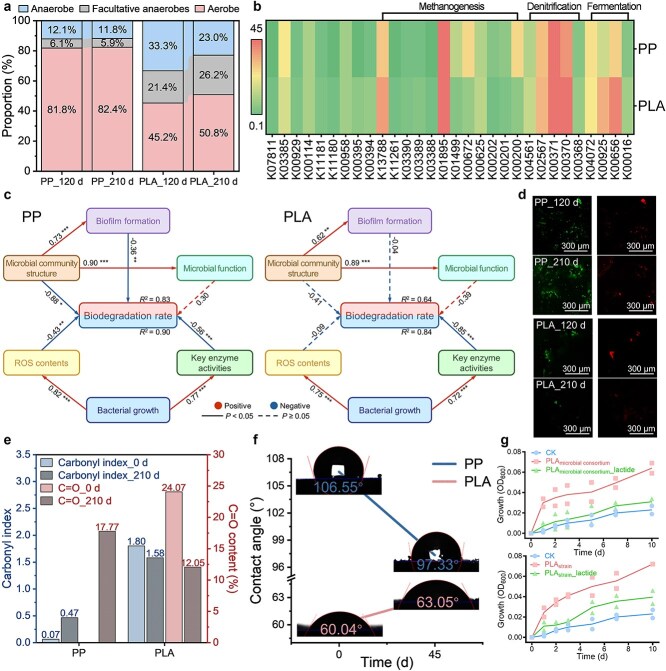
Biotic and abiotic factors driving biodegradability of MPs. (a) Relative abundance of anaerobic, facultatively anaerobic, and aerobic MP-degrading bacteria in PP and PLA biofilms based on PlasticDB and BacDive annotations. (b) Enrichment of anaerobic metabolic pathways under mesocosm incubation. (c) PLS-SEM showing the direct and indirect effects of various factors on MP biodegradation. ^*^P < .05, ^**^P < .01, and ^***^P < .001. (d) The distribution of live and dead bacteria in PP and PLA biofilms under mesocosm incubation. (e) Carbonyl index and C=O content under mesocosm incubation and (f) water contact angle under co-culture with the isolated strains. (g) Growth curves of PLA_strain_ and PLA_consortium_ with and without lactide. CK represents the control with no bacterial strain, consortium, or lactide.

This study provides the evidence that compared to conventional PP, biodegradable PLA not only displayed an inferior biodegradability, but also possessed more complex biodegradation routes. PP undergoes stable and progressive biodegradation over time, whereas PLA breaks down rapidly in the initial stage and then become recalcitrant afterward. The biodegradation of PLA and PP were driven by a multitude of biotic and abiotic factors. First of all, the diversity, composition, and function of microbial communities varied between PP and PLA biofilms (Figs 2 and 6a and b), altering the biodegradability of MPs. Oxygen tolerance annotation revealed that the aerobes maintained predominance (81.8%–82.4%) among MP-degrading bacteria in PP biofilm between 120–210 days of mesocosm incubation. Conversely, facultative and obligate anaerobes accounted for high proportions (49.2%–54.7%) of MP-degrading bacteria in PLA biofilm (Fig. 6a). The community variations mainly arise from the difference in MP density; low-density PP (0.90–0.91 g/cm^3^) remains buoyant in surface water [14], whereas high-density PLA (1.25–1.28 g/cm^3^) tends to sink into deeper water [15]. Additionally, the anaerobic pathways related to methanogenesis, denitrification, and fermentation were considerably enriched in PLA biofilm relative to PP biofilm (Fig. 6b) [68, 69]. The *hdrA2, mttB*, and *ackA* genes related to methane metabolism were upregulated in PLA biofilm (Fig. 2g). In contrast, PP biodegradation was predominantly governed by aerobic oxidation. These results can be supported by PLS-SEM, where ROS production exhibited a significant effect on biodegradation of PP (*β* = −0.434, *P* < .01), but not PLA (*β* = −0.087, *P* > .05; Fig. 6c). In principle, microbial communities in aerobic environments are more abundant and reactive than those in hypoxic/anoxic environments, accelerating pollutant degradation [70, 71]. Our study showed that the microbial diversity and abundance in PP biofilm increased during prolonged incubation (Figs 2b and 6d), which acted as a major contributor to the higher biodegradation of PP over PLA. A previous study demonstrated that the biodegradation of biodegradable PLA was accelerated compared to that of conventional PE when artificially immobilized at equivalent water column depths. These results provide valuable information about the intrinsic biodegradability of MPs under controlled experimental conditions, whereas it may not fully reflect real-world scenarios, because MPs with different densities are distributed at various water depths, thereby leading to distinct environmental settings [72].

The distinct molecular structures of PP and PLA affected their intrinsic biodegradability in a time-dependent manner. PP containing stable C–C/C–H bonds required considerable energy for chain scission and oxidation, whereas the ester bonds of PLA were susceptible to hydrolases for depolymerization (Table S1) [73, 74]. Compared to PP, PLA featured abundant oxygen-containing functional groups (e.g. C–O/C=O) and high hydrophilicity (Fig. 6e and f), which facilitated microbial attachment and hence biodegradation during initial incubation (Figs 1 and 6d). The important role of oxygen-containing functional groups can be validated by the higher biodegradability of photoaged PP than its virgin counterpart (Fig. S34). As biodegradation progressed, the oxygen-containing functional groups in PLA were detached, accompanied by a slight increase in hydrophobicity (Figs 6e and f). As such, PLA became less favorable for microbial growth compared to its virgin counterpart (Figs 2b and 6d), leading to reduced biodegradability (Fig. 1). However, the opposite was true for PP, where degradation promoted the accumulation of C=O and –OH groups and chain scission (Figs 1h and 6e). This transformation not only enhanced microbial attachment, but also facilitated biodegradation of PP (Figs 1i and 6d).

The accumulation of differential metabolic products influenced the biodegradability of PP and PLA by regulating microbial growth and activity. The biodegradation products of PP, consisting of predominantly long-chain alkanes and oxidation derivatives, were metabolized *via β*-oxidation and TCA cycle (Fig. 4b). Lactide was the most abundant product of PLA (Fig. 4c), which had potential toxicological effects [75, 76]. To verify the effect of lactide on microbial growth, co-culture assay was conducted by adding 100 μM lactide into the medium containing PLA_strain_ and PLA_consortium_ [77]. Compared to the control, lactide addition reduced the growth of PLA_strain_ (54.9%) and PLA_consortium_ (48.4%; Fig. 6g). The collective findings illuminate that lactide accumulation inhibits microbial growth and metabolic activity, thereby lowering the biodegradability of PLA during long-term incubation.

Biodegradable plastics with environmental friendliness and easy degradability are produced as alternatives to conventional plastics. Our findings suggest potential environmental impacts of biodegradable MPs that have previously been overlooked. The initial rapid biodegradation of biodegradable MPs can result in substantial production of small fragments and hazardous byproducts. These components may persist in the environment over extended time periods, posing greater environmental risk than the original forms and even conventional MPs undergoing continuous biodegradation. The limitations of this study include a limited number of MP types used in laboratory experiments and the absence of in situ incubation attributable to heterogeneous environmental parameters. Future studies should carry out field incubation experiments with commonly used commercial plastics and assess the differential biodegradability of conventional and biodegradable plastic/MPs in other environmental matrices, such as soil and atmosphere.

## Supplementary Material

Supplementary_material_wrag167

## Data Availability

All data are available in the main text or the Supplementary Materials. The high-throughput sequencing and whole-genome sequencing data have been submitted to the NCBI Sequence Read Archive (SRA) database under BioProject accession number PRJNA1454712. The metagenomic sequencing data have been submitted to the NCBI SRA database under BioProject accession number PRJNA1454199. The metabolomic and proteomic profiling data were provided in Supplementary Tables S13 and S14, respectively.
