# Supplementary material for: Density-mediated freshwater plastisphere microbiomes preferentially degrade conventional rather than biodegradable microplastics
Source: ISME J. 2026 Jun 27;20(1):wrag167. doi: 10.1093/ismejo/wrag167 (PMC13403190; doi:10.1093/ismejo/wrag167)
Supplement: Supplementary_material_wrag167 [file supplementary_material_wrag167.zip › SM.docx]

Supplementary Materials for

**Density-mediated freshwater plastisphere microbiomes preferentially degrade conventional rather than biodegradable microplastics**

**Authors**

Haiyu Zhang^1,2†^, Peng Liu^1†^*, Yiqi Chen^1^, Jing Lv^1^, Xinrui Zhang^1^, Jiale Zhang^1^, Yifan Sun^1^, Chaoyi Wang^1^, Shichen Wei^1^, Xiaojuan Wang^1^*, Shixiang Gao^3^, Xun Qian^1^, Hanzhong Jia^1^*, James M. Tiedje^1,4^

**Affiliations**

1. State Key Laboratory of Soil and Water Conservation and Desertification control, College of Natural Resources and Environment, Northwest A & F University, Yangling 712100, China.

2. Key Laboratory of Pollution Processes and Environmental Criteria (Ministry of Education), Tianjin Key Laboratory of Environmental Remediation and Pollution Control, College of Environmental Science and Engineering, Nankai University, Tianjin, 300350, China.

3. State Key Laboratory of Water Pollution Control and Green Resource Recycling, School of Environment, Nanjing University, Nanjing 210023, China.

4. Center for Microbial Ecology, Michigan State University, East Lansing, MI 48824, USA.

*Corresponding Author: Peng Liu, Xiaojuan Wang, Hanzhong Jia

Email: pengliu@nwafu.edu.cn (P. Liu), xiaojuan7069@sina.com (X. Wang), jiahz@nwafu.edu.cn (H. Jia)

^†^These authors contributed equally to this work.

**This PDF file includes:**

Supplementary Text

Figs. S1 to S34

Tables S1 to S12

References

Supplementary Text

**Field sampling of plastic waste and determination of natural water parameters**

**Field sampling of plastic waste and natural water:** A total of 202 plastic samples were collected from 6 important river systems in China: Yangtze River and Changhu Lake (Hubei), Pearl River (Guangdong), Haihe River tributary (Tianjin), Weihe River (Shaanxi), and Kashigaer River (Xinjiang) in China during October 2022 and May 2025 (Fig. S1). After eliminating similar types, 111 types of plastics were selected for the determination of chemical composition, biofilm biomass, and biodegradation properties (Fig. S2). Natural freshwater samples were collected from Weihe River in October 2022, filtered through a 2 mm sieve, and mixed for incubation experiments. The background microplastic (MP) concentration in the freshwater sample was 275 particles/L, determined by Micro-Raman technique [1, 2], which exhibited minimal interference with incubation experiments. The freshwater sample had an initial pH value of 8.70 and contained 10.59 mg/L of dissolved oxygen, 15.67 mg/L of total organic carbon, 3.03 mg/L of total nitrogen, and 0.08 mg/L of total phosphorus.

**Background MP concentration:** The background concentration of MPs in freshwater sample was quantified using Micro-Raman technique [1, 2]. A 2 mL water sample was digested with 50 mL of HNO_3_ in a water bath maintained at 50°C for 30 min. Digested suspensions were vacuum-filtered through polytetrafluoroethylene membranes (0.45 μm pore size), and then subjected to ultrasonic dispersion in anhydrous ethanol for 15 min. Subsequently, the ethanol suspension was vacuum-filtered onto a fresh polytetrafluoroethylene membrane, freeze-dried and analyzed using a push-broom micro-Raman imaging system (DXR3xi; Thermo Fisher Scientific, Waltham, USA). The sample was analyzed using a five-point method, where the membrane was divided into 9 areas and each contained 5 scan points (upper, middle, lower, left, and right), totaling 45 scan points. The instrumental parameters were set as follows: Laser wavelength of 785 nm, laser power of 15 mW, exposure time of 0.50 sec (2 Hz) and 50 scans. All particles at each scan point were analyzed, with the spectral range set from 300 to 3500 cm^–1^. The calculated background MP concentration was 275 particles/L, which was significantly lower than the addition of 92,000 particles/L (1.25 g/L) in mesocosm incubation experiments, indicating minimal interference from background MPs.

**pH:** The pH of water samples was measured using a pH meter (PHS-3C; INESA, Shanghai, China).

**Dissolved oxygen:** Dissolved oxygen was measured using an electrochemical probe method (HQ30D; Hach Company, Loveland, USA) [3].

**Total organic carbon:** Total organic carbon was quantified using a total organic carbon analyzer (Element, Langenselbold, Germany).

**Total nitrogen:** Total nitrogen was determined by the alkaline potassium persulfate digestion protocol coupled with ultraviolet-visible (UV-vis) spectrophotometric analysis [4]. Initially, 10 mL of water sample and 5 mL of alkaline potassium persulfate solution (600 mL of 6.7% (*w*/*v*) K_2_S_2_O_8_ mixed with 300 mL of 5.0% (*w*/*v*) NaOH, diluted to 1000 mL) were added to a digestion tube. The mixture was digested in a high-pressure steam sterilizer at 121°C for 30 min. After cooling, 1 mL of HCl was added and the volume was adjusted to 25 mL with ultrapure water. The solution was mixed and the absorbance was measured at 220 nm and 275 nm using a UV-vis spectrophotometer (GENESYS 50; Thermo Fisher Scientific). To correct for interferences, the absorbance was calculated as follows:

*A*_b_=*A*_b220_ – 2*A*_b275_ (1)

*A*_s_=*A*_s220_ – 2*A*_s275_ (2)

*A*_r_=*A*_s_ – *A*_b_ (3)

where *A*_b_ represents the corrected absorbance of the blank, with *A*_b220_ and *A*_b275_ represent the blank absorbances at 220 nm and 275 nm, respectively, *A*_s_ is the corrected absorbance of a standard solution, *A*_s220_ and *A*_s275_ represent the standard absorbances at 220 nm and 275 nm, respectively, *A*_r_ represents the difference between the corrected absorbance of the standard and blank. A calibration curve was constructed by plotting total-nitrogen content (µg N) on the abscissa against the corresponding absorbances on the ordinate. Using this curve, the total nitrogen concentration of each sample was calculated and expressed as mg N/L.

**Total phosphorus:** Total phosphorus was determined using ammonium molybdate spectrophotometric method with potassium persulfate as the oxidant [5]. 25 mL of water sample and 4 mL of potassium persulfate solution were added to a digestion tube and digested in a high-pressure steam sterilizer at 121°C for 30 min. After cooling, ultrapure water was added for dilution to 50 mL. Subsequently, 1 mL ascorbic acid was introduced, and after 30 seconds, 2 mL ammonium molybdate solution [100 mL of 13.0% (*w*/*v*) (NH_4_)_6_Mo_7_O_24_·4H_2_O was added to 300 mL H_2_SO_4_ (1:1 v/v) then mixed with 100 mL of 0.4% (*w*/*v*) potassium antimonyl tartrate solution] was mixed in. The absorbance was measured at 700 nm using a UV-vis spectrophotometer (GENESYS 50; Thermo Fisher Scientific). A working curve was prepared by treating 0.0, 0.5, 1.0, 3.0, 5.0, 10.0, and 15.0 mL aliquots of standard phosphate solution as described above; the resulting absorbance was plotted against the corresponding phosphorus mass (*m*, µg) to obtain a linear calibration. For each sample, the phosphorus mass was interpolated from this curve, blank-corrected, and the total phosphorus concentration was calculated with equation (4).

$C=\frac{m}{V}$ (4)

where *C* is the total phosphorus concentration (mg P/L) and V is the undigested sample volume (25 mL). Results were reported to two decimal places.

**Experimental materials and chemicals**

**Preparation of virgin and photoaged MP samples:** Polypropylene (PP) and polylactic acid (PLA) samples were purchased from Fengtai Polymer Material Co., Ltd. (Dongguan, China), with particle sizes of 0.34 mm and 0.38 mm, number-average molecular weight (Mn) of 65456 and 58290, and carbon content of 86.5% and 49.0%, respectively (Fig. S3, Table S1). The MP samples were washed three times with ethanol and deionized water to remove impurities and then air-dried at 45°C and stored in dark, sealed glass containers. Photoaged MPs were obtained using ultraviolet (UV) irradiation, because a long time period was needed to observe surface alterations under sunlight irradiation [6, 7]. Photoaging was conducted in a 30°C light chamber equipped with three 40-W UV fluorescent lamps under an irradiance intensity of 20.53 ± 2.61 W/m^2^. PP and PLA samples were placed in separate tin foil boxes and exposed to UV irradiation for 7 days, with stirring at 60 rpm to simulate wave disturbance. After irradiation, MP samples were collected, sealed in glass containers, and stored in dark. Compared to their virgin counterparts, photoaged PP and photoaged PLA showed cracking (Fig. S3), increased oxidation (2.0- and 1.4-fold), reduced particle size (–20.6% and –34.2%), and smaller molecular weight (Mn –27.8% and –93.0%; Table S1).

**Other materials and chemicals:** Biochemical assay kits for fluorescein diacetate (FDA) hydrolase, superoxide anion (O_2_^•–^), and hydrogen peroxide (H_2_O_2_) quantification were obtained from Solarbio (Beijing, China). HNO_3_ (AR), HCl (AR), H_2_SO_4_ (AR), and phenol (AR) were purchased from Keshi Chemical (Chengdu, China). Anhydrous ethanol (AR), KH_2_PO_4_ (AR), potassium antimonyl tartrate (AR), KNO_3_ (AR), crystal violet (AR), sodium tartrate (AR), KH_2_PO_4_ (AR), MgSO_4_·7H_2_O (AR), and NaCl (AR) were purchased from Guanghua Sci-Tech Co., Ltd. (Guangdong, China). K_2_S_2_O_8_ (AR), ammonium molybdate tetrahydrate ((NH_4_)_6_Mo_7_O_24_·4H_2_O) (AR), CuSO_4_·5H_2_O (AR), Na_2_CO_3_ (AR), FeSO_4_·7H_2_O (AR), ZnSO_4_·7H_2_O (AR), MnSO_4_·H_2_O, and methylene chloride (> 99.5%) were purchased from Kermel (Tianjin, China). Ascorbic acid (> 99.0%) and tetrahydrofuran (> 99.0%) were purchased from Macklin (Shanghai, China). Folin-Ciocalteu’s phenol reagent and propidium iodide (PI; > 95.0%) were purchased from Solarbio. Syto9 green fluorescent nucleic acid stain was acquired from Thermo Fisher Scientific (USA). K_2_HPO_4_·3H_2_O (AR) and KBr (AR) were purchased from Tianli Chemical Reagent Co., Ltd. (Tianjin, China) and Guangfu Chemical Reagent Co., Ltd. (Tianjin, China). Hexane (> 99.0%) and NaOH (AR) were supplied by CNW Technologies GmbH (Germany) and Xilong Scientific Co., Ltd. (Sichuan, China), respectively.

**Setup of abiotic control experiments**

To evaluate the abiotic changes of MPs in the absence of natural freshwater microbiota, abiotic control experiments were conducted using natural freshwater subjected to 30 min of autoclaved sterilization (121℃, 0.1 MPa), followed by HgCl_2_ treatment. Specifically, the natural freshwater in mesocosm incubation was pre-treated with high-temperature and high-pressure sterilization procedures for 30 min, and subsequently disinfected with HgCl_2_ to eliminate indigenous functional microorganisms. Thereafter, virgin PP, photoaged PP, virgin PLA, and photoaged PLA samples were added to the preprocessed abiotic control systems. The whole operational process and incubation conditions were similar to those of the experimental mesocosm groups. At 0, 60, 120, 180, and 210 days, 50 mL of water samples were collected, and all MPs were collected at 210 days by filtration through a 0.22 µm glass fiber filter. The collected samples were cleaned with phosphate-buffered saline, freeze-dried, and then stored in sealed containers at –20°C until use.

**Biofilm biomass analysis**

**Biofilm biomass analysis:** Biofilm biomass was quantified using a modified crystal violet staining method, which is widely used to quantify biofilm biomass due to its simple operation [8]. Specifically, 10 mg of MPs were mixed with 0.5 mL of 0.1% (*w*/*v*) crystal violet solution for 45 min. After staining, the MPs were washed three times with deionized water, air-dried, and decolorized by 5.0 mL of 95.0% ethanol for 10 min. The absorbance of the solution was measured at 595 nm using a UV-vis spectrophotometer (GENESYS 50; Thermo Fisher Scientific). Despite the wide use of this method, impurities within the biofilm may interfere with the staining signal and contribute to the absorbance values. Therefore, to provide a more accurate assessment of biofilm formation on MPs, the extracellular polymeric substances, including proteins and polysaccharides, were analyzed.

**Biofilm extraction:** 100 mg of incubated MPs were transferred to 10 mL of ultrapure water, followed by centrifugation at 8000 rpm for 15 min to yield a biofilm suspension. This suspension was used for the determination of extracellular polymeric substance (polysaccharide and protein content).

**Protein analysis:** A modified Lowry method was used for protein measurements, with bovine serum albumin as the standard [9]. A 1 mL aliquot of the biofilm suspension was mixed with 0.5 mL of 1 mol/L NaOH and vortexed for 30 min. Subsequently, 5 mL of sodium carbonate alkaline copper solution (1 mL of 1.0% CuSO_4_·5H_2_O mixed with 100 mL of 2.0% Na_2_CO_3_ and 1 mL of sodium tartrate) was added. After 10 min, 0.5 mL of 1 mol/L Folin-Phenol reagent was added. Following 30 min of incubation, the absorbance was measured at 500 nm using a UV-vis spectrophotometer (GENESYS 50; Thermo Fisher Scientific). Protein concentrations were calculated from a bovine serum albumin standard curve using the linear regression equation.

**Polysaccharide analysis:** A modified phenol-H_2_SO_4_ method was used to analyze the content of polysaccharides [10]. Specifically, 1 mL of biofilm suspension was thoroughly mixed with 1 mL of 5.0% phenol and 5 mL of H_2_SO_4_ in a glass bottle for 10 min. The bottle was then placed in a 30°C-water bath for 20 min and analyzed using a UV-vis spectrophotometer (GENESYS 50; Thermo Fisher Scientific) at a wavelength of 490 nm. Polysaccharide contents were calculated based on a glucose standard curve.

**Confocal laser scanning microscope:** MPs were washed with sterile water and stained with 3.34 μmol/L Syto9 solution and 20 μmol/L PI solution for 30 min [11]. Thereafter, MPs were rinsed again with sterile deionized water to remove the residual dye solution, placed on the glass slides, and observed by a confocal laser scanning microscope (FV3000; Olympus, Hachioji, Japan). The live and dead cells were stained green (Syto9) and red (PI), with corresponding excitation wavelengths at 488 nm and 561 nm, respectively.

**Microbial community analysis**

**High-throughput sequencing:** V3–V4 region 16S rRNA gene amplicon sequencing was sequenced to profile bacterial communities in water samples incubated for 0 and 210 days. Total genomic DNA was extracted from the samples using the TGuide S96 Magnetic Soil/Stool DNA Kit (Tiangen Biotech, China) following the manufacturer’s protocol. DNA integrity was verified by 1.8% agarose gel electrophoresis and concentration and purity were quantified using a NanoDrop 2000 UV-vis spectrophotometer (Thermo Fisher Scientific). The V3-V4 region was amplified with universal primers 338F (5’-ACTCCTACGGGAGGCAGCA-3’) and 806R (5’-GGACTACHVGGGTWTCTAAT-3’). PCR amplicons were purified using the Omega DNA purification kit (Omega Inc., Norcross, USA) and quantified with Qsep-400 (BiOptic, Taipei, Taiwan). The amplicon libraries were prepared for paired-end sequencing (2×250 bp) and sequenced on the NovaSeq 6000 System (Illumina, Biomarker Technologies, Beijing, China). Raw reads were processed using DADA2 to generate high-resolution amplicon sequence variants (ASVs), with low-abundance ASVs (≤ 2 reads across all samples) filtered to minimize noise [12]. Taxonomic classification was performed in QIIME2 using the SILVA 138.1 database and a Naive Bayes classifier at a 70.0% confidence threshold [13, 14].

Full-length 16S rRNA gene sequencing was performed to characterize bacterial communities in MP biofilm samples. The V1–V9 region of bacterial 16S rRNA genes was amplified using the universal primers 27F (5’-AGRGTTTGATYNTGGCTCAG) and 1492R (5’-TASGGHTACCTTGTTASGACTT), which were flanked by sample-specific PacBio barcode sequences for multiplexed library preparation. PCR amplicons were purified using VAHTSTM DNA Clean Beads (Vazyme, Nanjing, China), quantified with a Qubit 3.0 Fluorometer (Invitrogen, Waltham, USA), and pooled in equimolar ratios. SMRTbell libraries were constructed from the pooled amplicons using the SMRTbell Express Template Prep Kit 2.0 (Pacific Biosciences) and sequenced on the PacBio Sequel II System (Biomarker Technologies, Beijing, China) with Sequel II Binding Kit 2.0. Raw circular consensus sequencing reads were processed using DADA2 to generate high-resolution ASVs, with low-abundance ASVs (≤ 2 reads across all samples) filtered to minimize noise [12]. Taxonomic classification was performed in QIIME2 using the SILVA 138.1 database *via* a Naive Bayes classifier at a 70.0% confidence threshold [13, 14].

**Metagenomic sequencing:** Metagenomic sequencing was performed on MP biofilms after 210 days of incubation. DNA was extracted using the CTAB method and its quality, concentration, integrity, and purity were assessed using the Agilent 5400 system (Fragment Analyzer 5400; Agilent, Santa Clara, USA). The sequencing library was constructed using the NEBNext Ultra DNA Library Prep Kit for Illumina (NEB, Ipswich, USA). DNA was fragmented to a size of 350 bp with the Covaris M220 sonicator (Covaris, Massachusetts, USA). The DNA fragments underwent end polishing, A-tailing, and ligation with Illumina adapters, followed by PCR amplification. After PCR products were purified by the AMPure XP system (Beckman Coulter, Inc., Brea, USA), library quality was assessed on the Agilent 5400 system (Agilent, Santa Clara, USA) and quantified by QPCR (1.5 nM). The qualified libraries were sequenced using the PE150 strategy on the NovaSeq System (Illumina, USA), generating high-resolution microbial genomic data. The raw data from biofilm samples were obtained by metagenomic sequencing using the Illumina NovaSeq high-throughput sequencing platform. To ensure data reliability, raw sequencing data were preprocessed with Kneaddata (v0.7.4) to remove low-quality reads and contaminants [15, 16]. Taxonomic classification was performed using Kraken2 (v2.0.7-beta) against a custom-built microbial nucleic acid database, derived from the NCBI NT and RefSeq databases. Bracken (v2.0) was subsequently utilized to predict the relative abundance of species in the samples. For functional analysis, quality-controlled, and host-filtered sequences were aligned to the UniRef90 protein database using HUMAnN2 (based on DIAMOND) [17], generating functional annotations and relative abundance tables across various functional databases.

**Isolation of MP-degrading strains**

MP-degrading strains in biofilms were screened and enriched using an inorganic salt medium (Table S2) supplemented with 0.5% (*w*/*v* carbon content) PP or PLA as the sole carbon source (Fig. S4) [18]. Biofilm-formed MPs (0.05 g, 210 days) were inoculated into 250 mL culture flasks containing 150 mL of medium, because biofilms became more mature and adaptable to degrade MPs after prolonged incubation. The flasks were incubated at 30°C in the dark with shaking at 150 rpm for 7 days. The enrichment was repeated five times (35 days in total) to obtain stable microbial consortia. Agar plates were prepared using the inorganic salt medium supplemented with (0.5% *w*/*v* carbon content) MPs plus 1.0% agar to isolate key MP-degrading strains. Following a 1:100 dilution, 100 μL of the enriched consortia were spread onto replicate plates and cultured at 30°C for 7 days. Morphologically distinct colonies were picked and streaked onto fresh plates until pure isolates were obtained. One PP-degrading strain (PP_strain_) and one PLA-degrading strain (PLA_strain_) were isolated from MP biofilms based on their growth performance.

**Characterization of MP biodegradation properties**

**Biofilm removal:** The incubated MPs were initially lysed using 75.0% ethanol for 30 min, followed by disruption *via* ultrasonication using a KQ-300DE ultrasonic cleaner (CNC, Kunshan, China) for 30 min. The bacterial lysate was discarded and the MPs were washed three times with ultrapure water to remove remaining bacterial debris. Finally, the MPs were dried at 45°C for 12 hours. The biofilm removal efficiency was assessed by the changes in biofilm biomass, which is calculated using equation (5):

$\text{Removal efficiency}\text{ = (}\text{A}_{\text{MPs\_biofilm}}\text{ }\text{–}\text{ }\text{A}_{\text{MPs}}\text{)/}\text{A}_{\text{MPs\_biofilm}}$ (5)

where *A*_MPs_biofilm_ denotes the biofilm biomass of the incubated MPs and *A*_MPs_ denotes the biofilm biomass of the MPs after biofilm removal. This method removed at least 70.0–100.0% of the biofilm biomass on PP, PLA, photoaged PP, and photoaged PLA (shown in Fig. S6), minimizing potential interference of biofilms in subsequent analyses. This method is gentle with no modification to the surface characteristics or chemical properties of the MPs [19, 20].

**Particle size analysis:** The particle size distribution of MPs was quantified by the combined method of scanning electron microscopy (SEM; Nova NanoSEM 450; FEI Corp., Hillsboro, USA) observation and Nano Measurer software (v1.2.5; Fudan University, Shanghai, China), which was widely adopted and used in a large number of published studies [21, 22]. To ensure statistical reliability, at least 100 particles were measured for each sample and measurements were conducted in at least two orientations to account for particle irregularities [23]. At the same time, three replicates for each sample were conducted in order to reduce the testing error. To model the degradation kinetics of MPs, we fitted the degradation curves to the average particle size data of PP and PLA using two different approaches: a linear regression model for PP (equation 6) and an exponential decay model for PLA (equation 7) [24]. These models were chosen based on the observed degradation patterns of the MPs, with PP exhibiting a linear reduction in particle size, whereas PLA showed an initial rapid degradation followed by stabilization over time. The models are expressed as follows:

$\text{S}\text{ = –}\text{kt}\text{ }\text{+}\text{ }\text{S}_{\text{0}}$ (6)

$\text{S}\text{ = (}\text{S}_{\text{0}}\text{ }\text{–}\text{ }\text{S}_{\text{eq}}\text{)}\text{ }\text{e}^{\text{–kt}}\text{ }\text{+}\text{ }\text{S}_{\text{eq}}$ (7)

where *S* represents the particle size of MPs, *S*_eq_ is the predicted equilibrium particle size, *S*_0_ is the initial particle size, *t* is the incubation time, and *k* is the degradation rate constant. The goodness of fit for each model was evaluated using *R*^2^, demonstrating that both models favorably described the respective fragmentation kinetics.

**Fully automatic physical adsorption analyzer:** The gas-accessible specific surface areas of MPs were measured using N_2_ adsorption-desorption analysis on a fully automatic physical adsorption analyzer (ASAP 2460, Micromeritics, USA). Before analysis, all samples were degassed at 70°C for 6 h. N_2_ was used as the adsorption gas, and the specific surface area was calculated using the Brunauer-Emmett-Teller (BET) method when a reliable BET linear region was obtained.

**Fourier transform infrared (FTIR) spectroscopy:** The oxidative degradation of MPs was determined by Vetex70 FTIR spectrometer (Vetex70; Bruker, Ettlingen, Germany). The MPs were placed on an automatic scanning stage with a spectral range of 500–4000 cm^–1^. 16 scans were taken per sample and the spectra were treated with atmosphere correction. Based on FTIR data, carbonyl index were calculated using the maximum intensity of the carbonyl group (I_1714_ for PP, polyethylene glycol terephthalate (PET), and polyethylene (PE) and I_1760_ for PLA) relative to the methylene group (I_977_ for PP, I_971_ for PET, I_1471_ for PE, and I_1452_ for PLA) [25-28]. Also, the hydroxyl index was calculated using the maximum intensity of the hydroxyl group (I_3348–3369_ for PP, PET, and PE and I_3502_ for PLA) relative to the methylene group (I_977_ for PP, I_971_ for PET, I_1471_ for PE, and I_1452_ for PLA) [29, 30]. The carbonyl and hydroxyl index values were normally used to characterize the oxidation degree of plastics/MPs, which have been widely used in previous studies [31-33].

**X-Ray photoelectron spectroscopy (XPS):** XPS measurements were performed using a Nexsa spectrometer (Nexsa; Thermo Fisher Scientific), which could detect most essential elements except hydrogen on the nanometer-thick surface of MPs. High-resolution core-level spectra were collected using an Al Kα source, with survey and detailed scans performed at pass energies of 1 eV and 0.1 eV, respectively. The standard C 1s at 284.8 eV was used as the reference.

**Thermal gravimetric analysis:** Thermal gravimetric analysis 4000 (PerkinElmer, Waltham, USA) was applied to characterize thermal property during biodegradation process of MPs. The analysis involved subjecting the samples to a controlled heating process, ranging from 50 to 500°C at a heating rate of 20°C/min, under constant nitrogen flow.

**Gel Permeation chromatography:** The gel permeation chromatography (GPC) measurement of PLA was performed on an Agilent 1260 system (Agilent, Santa Clara, USA) equipped with a refractive index (RI) detector and a PLgel MINED-B LS (300×7.5 mm), using tetrahydrofuran as the mobile phase. For PP, the analysis was conducted using high-temperature GPC on an Agilent PL-GPC 220 (Agilent Technologies, Santa Clara, USA) unit equipped with RI detectors and two PLgel MIXED-B LS columns (300×7.5 mm). The HT-GPC analysis was conducted at 150°C with 1,2,4-trichlorobenzene as the mobile phase. Samples were prepared by dissolving 0.1 wt% of the polymer in the mobile phase solvent within an external oven.

**Calculation of integrated biodegradation rate (IBR):** To quantitatively compare the biodegradability between PP and PLA, IBR was calculated by integrating multiple biodegradation properties—weight, carbon content, molecular weight (Mn and weight-average molecular weight (Mw)), carbonyl index, oxygen-to-carbon (O/C) ratio, pyrolysis temperature (T_m_), and particle size. This calculation builds on the Integrated Biomarker Response approach, which is widely used in the comprehensive analysis of biomonitoring [34, 35]. We refined and optimized this method to integrate all critical parameters associated with MP biodegradation, including subtle and non-significant variations, to improve the sensitivity and comparability of biodegradation assessments. This integrated approach allows direct quantitative comparison of degradation capacities among different materials, overcoming the limitations of single-parameter assessments.

The change rate of each degradation parameter was calculated using equation 8:

$\text{Change rate = (original value}\text{ }\text{–}\text{ }\text{degradation value)/original value}$ (8)

Because oxidation parameters exhibited an increase during biodegradation, the change rate was calculated using equation 9:

$\text{C}\text{hange rate}\text{ = (}\text{oxidation value}\text{ }\text{–}\text{ }\text{original value}\text{)/}\text{original value}$ (9)

Finally, the IBR was obtained by integrating the change rates of all individual parameters using the following equation 10:

$\text{IBR}\text{ = Σ(}\text{Change rates of all parameters}\text{)/}\text{N}$ (10)

where Σ represents the sum of change rates of all parameters and *N* is the total number of parameters.

**Gas chromatography-tandem mass spectrometry (GC-MS/MS) technique:** To reveal the biodegradation routes of the isolated strains for PP and PLA, the biodegradation products were detected by GC-MS/MS (Trace 1610-TSQ 9610; Thermo Fisher Scientific). At 10, 20, and 45 days, culture suspensions were filtered through 0.22 μm glass fiber membrane to remove MPs. The resulting filtrates were then subjected to solid-phase extraction using an Agilent Bond Elut C18 cartridges (500 mg, 6 mL). Hexane and methylene chloride were used for extracting degradation products of PP and PLA, respectively. The C18 cartridges were conditioned with 10 mL of hexane for PP and methylene chloride for PLA, followed by rinsing with 15 mL of ultrapure water to remove residual impurities. The sample was slowly added to the C18 column and passed through the solid-phase extraction column at a flow rate of < 5 mL/min to facilitate the enrichment of target compounds. Following sample elution, 5 mL of ultrapure water was introduced to rinse the column of residual contaminants. The column was then air-dried, followed by sequential elution with 5 mL hexane for PP and 5 mL methylene chloride for PLA, and the eluate was collected sequentially. The extract was concentrated to 2 mL *via* nitrogen gas drying. The GC oven program started at 40°C for 3 min, followed by a linear ramp of 10°C/min to 280°C and maintained for 5 min), with a total chromatographic running time of 33 min. The key parameters were set as follows: solvent delay time 4 min; transfer line temperature 250°C; ion source temperature 280°C; scan time 0.3 s. The samples were injected in pulsed splitless mode at 225°C, with an injection volume of 2 μL. The mass spectrometer worked in an electron impact (EI) mode at 70 eV, using a non-targeted approach in the full scan mode. The compounds for which no standards were available were tentatively identified with the help of the NIST and Willey mass spectrometer libraries (https://sciencesolutions.wiley.com/) when the correlation was greater than 95.0%, which indicates a great similarity between the MS spectrum of the identified compounds in the PP and PLA and that of the MS database.

**Determination of FDA hydrolase activity and reactive oxygen species (ROS) contents**

FDA hydrolase activity in the co-culture medium of MPs and strains was assayed using commercial kits (Solarbio Science, Beijing, China). FDA, a non-fluorescent compound, undergoes enzymatic hydrolysis to release stable fluorescein *via* a dehydration reaction, which can be measured at a wavelength of 490 nm. The contents of intracellular/extracellular O_2_^•–^ and H_2_O_2_ were determined using ROS detection kits (Solarbio Science) according to the manufacturer’s instructions. Although both ROS are mainly generated intracellularly, they can diffuse across cell membranes or export *via* membrane vesicles [36]. Determination of O_2_^•–^ content involved the reaction of O_2_^•–^ with hydroxylamine hydrochloride to form nitrite (NO_2_^–^), and subsequent reaction of NO_2_^–^ with sulfanilamide and N-1-naphthylethylenediamine dihydrochloride to yield a purple azo compound (which shows a characteristic absorption peak at 530 nm). H_2_O_2_ quantification relied on the reaction with titanium sulfate to form yellow titanium-peroxide complex with a characteristic absorption at 415 nm. Although hydroxyl radical (•OH) is known to contribute to oxidative degradation, the short half-life prevents direct quantification. Therefore, •OH level was assessed based on the upregulation of related antioxidant enzymes and iron transporter proteins revealed by proteomic analysis and the accumulation of ROS precursors (O_2_^•–^ and H_2_O_2_) [37, 38].

**Multi-omics analysis of MP biodegradation by the isolated strains**

**Whole-genome** **sequencing:** To identify bacterial species and investigate the MP biodegradation-related genes, whole-genome sequencing was performed. Genomic DNA was extracted from pure cultures of PP_strain_ and PLA_strain_ using the TIANamp Bacteria DNA Kit (Tiangen Biotech). The extracted DNA was sequenced on the NovaSeq System (Illumina, PE150) by Beijing Biomarker Technologies, following standardized protocols for library preparation, quality control, and sequencing. The bioinformatics analysis included five major steps: raw data quality control, genome assembly, species annotation, gene functional annotation, and genome visualization. In brief, the quality control of raw short reads was performed in Trim. Then, the clean short reads and long reads were co-assembled to reconstruct complete genomes using the Spades assembler (v3.6.2; https://github.com/ablab/spades) to generate complete sequences. Coding genes prediction was performed by Prodigal (v2.6.3; https://github.com/hyattpd/Prodigal). Species annotation was performed using the genome taxonomy database (https://gtdb.ecogenomic.org/) and databases such as the Kyoto Encyclopedia of Genes and Genomes (KEGG, https://www.genome.jp/kegg/) and Gene Ontology (GO, https://www.geneontology.org/) were used for functional annotation.

**Metabolomic profiling:** Metabolomic analysis was conducted to investigate the metabolite profiles of PP and PLA following 45 days of biodegradation by PP_strain_ and PLA_strain_. The analysis was conducted using a Waters Acquity I-Class PLUS UPLC system (Waters Corporation, Milford, USA) coupled with a Waters Xevo G2-XS QTof high-resolution mass spectrometer (Waters Corporation) equipped with a Waters Acquity UPLC HSS T3 column (1.8 µm, 2.1×100 mm). For both positive and negative ion modes, the mobile phases consisted of 0.1% formic acid (FA) in water (phase A) and 0.1% FA in acetonitrile (phase B), with an injection volume of 1 µL. Mass spectrometry data were acquired in MSe mode using the MassLynx V4.2 software, with dual-channel data acquisition at low collision energy (2 V) and high collision energy (10–40 V), with a scan rate of 0.2 seconds per mass spectrum. Electrospray ionization (ESI) parameters included a capillary voltage of 2000 V (positive ion mode) or –1500 V (negative ion mode), cone voltage of 30 V, ion source temperature of 150°C, desolvation gas temperature of 500°C and flow rates of 50 L/h for backflush gas and 800 L/h for desolvation gas.

Raw data from MassLynx v4.2 were processed using Progenesis QI software for peak extraction, alignment and metabolite identification and metabolite identification was performed using the Progenesis QI online METLIN database and Biomark’s self-built library, with a mass deviation threshold of ≤100 ppm for theoretical fragment matching [39]. The original peak area data were normalized to the total peak area, followed by principal component analysis and Spearman correlation analysis to evaluate sample repeatability and quality control. Pathway analysis of identified metabolites was conducted using the KEGG (https://www.genome.jp/kegg/), Human Metabolome Database (https://hmdb.ca/) and Lipid Maps databases (https://lipidmaps.org/). Differential metabolites were identified through fold change analysis and Student’s t-tests (*P* < 0.05). Orthogonal partial least squares discriminant analysis was performed using the R package ropls and the model’s reliability was validated through 200 permutation tests [40]. Differential metabolites were screened based on fold change (FC > 1), *P* value (*P* < 0.05), and variable importance in projection (VIP > 1). The significance of KEGG pathwayenrichment for differential metabolites was assessed using a hypergeometric distribution test.

**Proteomic profiling:** Proteomic analysis was conducted to characterize the protein profiles of PP and PLA following 45 days of biodegradation by PP_strain_ and PLA_strain_. Samples were prepared by lysing the cultures in lysis buffer (50 mmol/L NH_4_HCO_3_, pH 7.4, 10 mmol/L MgCl_2_, 7 mol/L urea, and 2 mol/L thiourea), followed by 5 min of ultrasonication on ice. The lysate was centrifuged at 12,000 g for 15 min at 4°C and the supernatant was collected. Protein concentration was determined using the Bradford assay. For protein digestion, 100 μg of protein from each sample was reduced with 10 mmol/L dithiothreitol for 1 h at 56°C and alkylated with iodoacetamide for 1 h at room temperature in the dark. The protein was digested with Trypsin Gold (Promega) at a 1:50 enzyme-to-substrate ratio for 16 h at 37°C. Peptides were desalted with a C18 cartridge and dried by vacuum centrifugation. Shotgun proteomics analyses were performed using a U3000 UHPLC system (Thermo Fisher Scientific) coupled with an Orbitrap Fusion mass spectrometer (Thermo Fisher Scientific) in data-dependent acquisition (DDA) mode [41]. A 1 μg sample of total peptides was injected onto a home-made C18 Nano-Trap column (2 cm×100 μm, 3 μm). Peptides were separated on an analytical column (25 cm × 75 μm, 100 Å pore size) using a 120-minute linear gradient of eluent B (0.1% formic acid in 80.0% acetonitrile/20.0% water) in eluent A (0.1% formic acid in water) at a flow rate of 350 nL/min. The mass spectrometer operated in DDA mode, acquiring a single full-scan mass spectrum in the Orbitrap (250–1450 m/z, 120,000 resolution) followed by data-dependent MS/MS scans (3-second cycle) in an Ion Routing Multipole at 30.0% normalized collision energy. For data-independent acquisition (DIA), samples were reconstituted in 0.1% formic acid, spiked with 0.2 μL of iRT standard peptides (Biognosys) and analyzed under identical chromatographic conditions. DIA parameters included an MS1 resolution of 120,000, MS2 resolution of 30,000, an m/z range of 350–1350 and 60 variable cycles. The full scan AGC target was set to 4×10^6^, with an injection time of 50 ms.

Raw data from DDA and DIA modes were processed using Proteome Discoverer 2.4 (Thermo Fisher Scientific), Biognosys Spectronaut 13 and the R statistical framework. DDA data were searched against a protein database with the following parameters: cysteine carbamidomethylation as a fixed modification; N-terminal acetylation and methionine oxidation as variable modifications; a maximum of two missed cleavages; and a 1.0% false discovery rate (FDR) threshold. Label-free quantification (LFQ) was performed using the MaxLFQ algorithm for MS1-based quantification [42]. For DIA data, MS2-based LFQ was executed in Spectronaut v13 with dynamic data extraction (q-value cutoff: 0.01) and protein quantities were calculated as the average precursor intensity [43]. Functional annotation was performed using InterProScan-5 against the non-redundant protein database for GO (https://www.geneontology.org/) and InterPro (https://www.ebi.ac.uk/interpro/) terms. Protein families and pathways were analyzed using Clusters of Orthologous Groups (COG) and KEGG databases [44]. Protein-protein interaction networks were predicted using the STRING-db server and GO/KEGG pathway enrichment analysis was conducted using a hypergeometric test-based pipeline [41].

**Statistical analysis**

Microbial *α*-diversity was calculated and displayed by the QIIME2 and R software, respectively [13]. Beta diversity was determined to evaluate the degree of similarity of microbial communities from different samples using QIIME. One-way analysis of variance followed by Tukey’s test was performed using GraphPad Prism (v9.5.0; https://www.graphpad.com/) to assess the differences in microbial diversity between PP and PLA biofilms at different incubation time (*P* < 0.05). To construct microbial co-occurrence networks, Spearman correlation coefficients (*ρ*) among ASVs were computed using the vegan package in R (v4.3.1; https://cran.r-project.org/package=vegan). Gephi (v0.10.1; https://gephi.org/) was used to visualize the co‑occurrence networks of biofilm bacteria across PP or PLA. Nodes were colored to highlight MP‑degrading genera, illustrating possible ecological interactions relevant to MP degradation. Nodes and edges with *P* < 0.001 and ρ > 0.95 were selected for network construction.

Partial least squares structural equation modeling (PLS-SEM) analysis was performed using SmartPLS 4 software to elucidate biodegradation mechanisms of PP and PLA in mesocosm and co-culture systems [45]. The variables for mesocosm biodegradation included biodegradation properties (weight, carbon content, molecular weight, carbonyl index, T_m_, and particle size), biofilm formation (biomass, polysaccharide, and protein), microbial community structure (ASVs count, Observed ASVs, Shannon index, MP-degrading strains, and Firmicutes abundance), and microbial function (glycolysis/gluconeogenesis, pyruvate metabolism, tricarboxylic acid (TCA) cycle, methane metabolism, and metabolism of xenobiotics by cytochrome P450). The variables for co-culture biodegradation included biodegradation properties (molecular weight, water contact angle, weight, and particle size), ROS contents (H_2_O_2_ and O_2_^•−^), and key enzyme activities (FDA, aldehyde/ketone reductase, dehydrogenase, acetyl-coenzyme A (acetyl-CoA) acetyltransferase, and catalase). The relationships among variables were quantified using path coefficients and the model fit was assessed based on the *R*^2^ values and path coefficients. To validate the stability and accuracy of the model, Bootstrap analysis (5000 resamples) was used to assess the significance of the path coefficients at *P* < 0.05. For PP and PLA, PLS-SEM explained 79.8% and 54.1% of the variance in mesocosm biodegradation, and 91.8% and 85.3% of the variance in co-culture biodegradation.

**Data results about abiotic control groups**

SEM images showed that, compared to original MPs at 0 day, the surface morphology and particle size of MPs showed less changes, with relative smooth surface in the abiotic control after 210 days (Fig. S19A). However, the MPs in experimental incubation developed clear holes, cracks, and different degrees of fragmentation, indicating more pronounced surface erosion and fragmentation under natural freshwater incubation (Fig. S14). Particle size analysis further supported this observation. After 210 days of incubation in the abiotic control, the particle sizes of virgin PP, virgin PLA, photoaged PP, and photoaged PLA decreased by only 2.9%, 5.3%, 3.7%, and 4.0%, respectively (Fig. S19A), which were much lower than decreased particle sizes at 26.5%, 26.3%, 29.6%, and 8.0%, respectively, of corresponding MPs in experimental incubation (Fig. S15). These results indicate that abiotic factors caused limited changes in surface morphology and particle size of MPs during mesocosm incubations. Therefore, the pronounced surface alterations and fragmentation of MPs were mainly caused by the microbial degradation. Analysis in chemical properties further validated the similar results. FTIR spectra showed only slight differences between original MPs and 210 d-incubated MPs in abiotic control (Fig. S19B). However, MPs incubated in experimental groups showed more pronounced changes after 210 days of incubation (Fig. S16). Similar results were obtained for the carbonyl index and O/C ratio. The variation degrees in carbonyl index and O/C ratios of virgin and photoaged MPs in experimental groups were higher than those of original and 210 d-incubated MPs in the abiotic control groups (Fig. 1E, Fig. S18, Table S5), indicating that microbial action in natural freshwater promoted oxidative degradation on MPs. Carbon content analysis showed that the carbon content of MPs displayed slight decrease in the abiotic controls, whereas the decrease magnitude was obviously lower than that observed in the experimental groups (Fig. 1G). Together, the results in abiotic control groups demonstrated that abiotic factors resulted in minor degradation of MPs, and the degradation of MPs during long-term freshwater incubation were mainly caused by the biofilm microbiota.


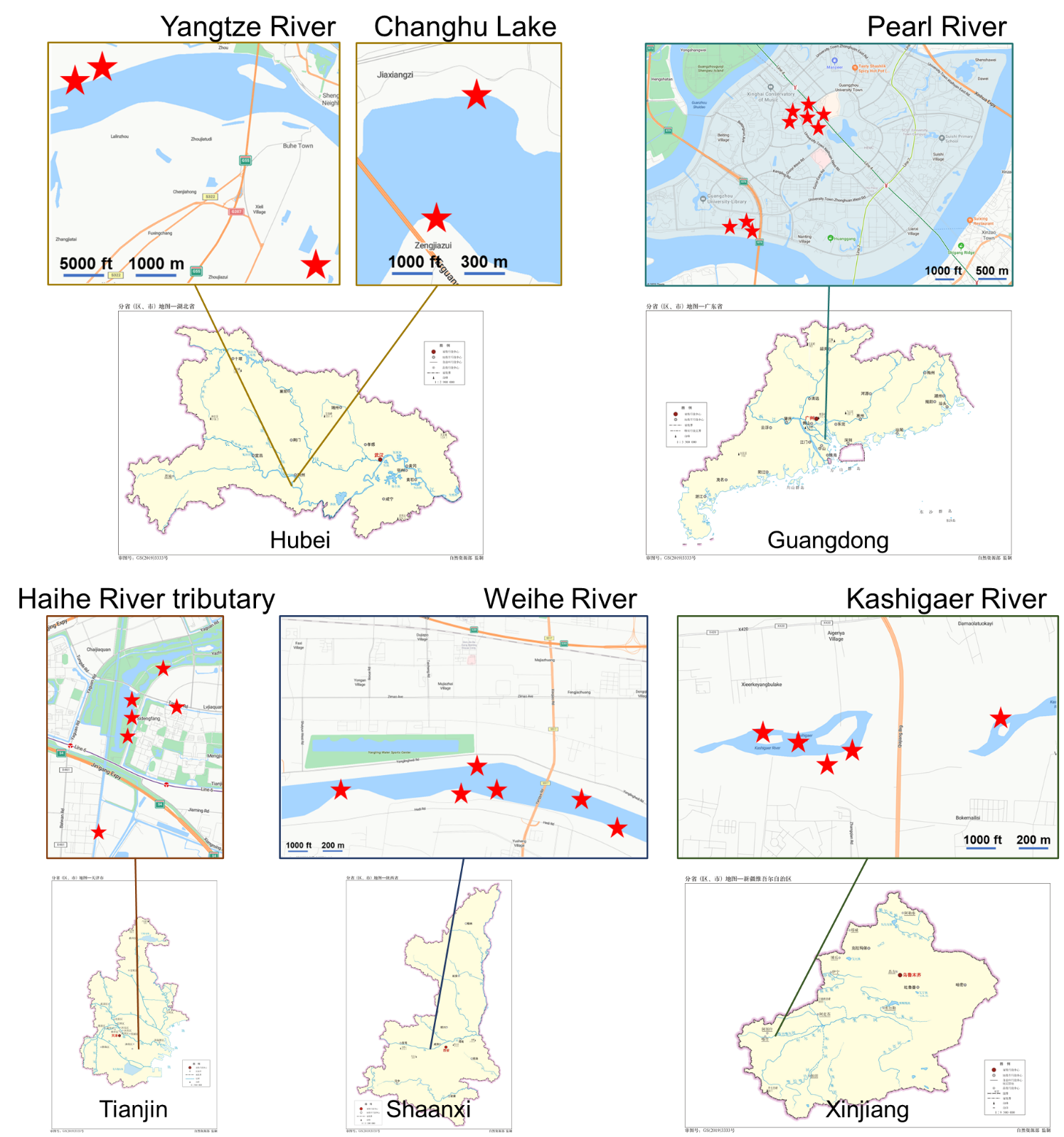


# **Fig. S1. Distribution of sampling sites in** **several important river systems in China (shown as red stars).** Sampling was conducted in Yangtze River and Changhu Lake (Hubei), Pearl River (Guangdong), Haihe River tributary (Tianjin), Weihe River (Shaanxi), and Kashigaer River (Xinjiang).


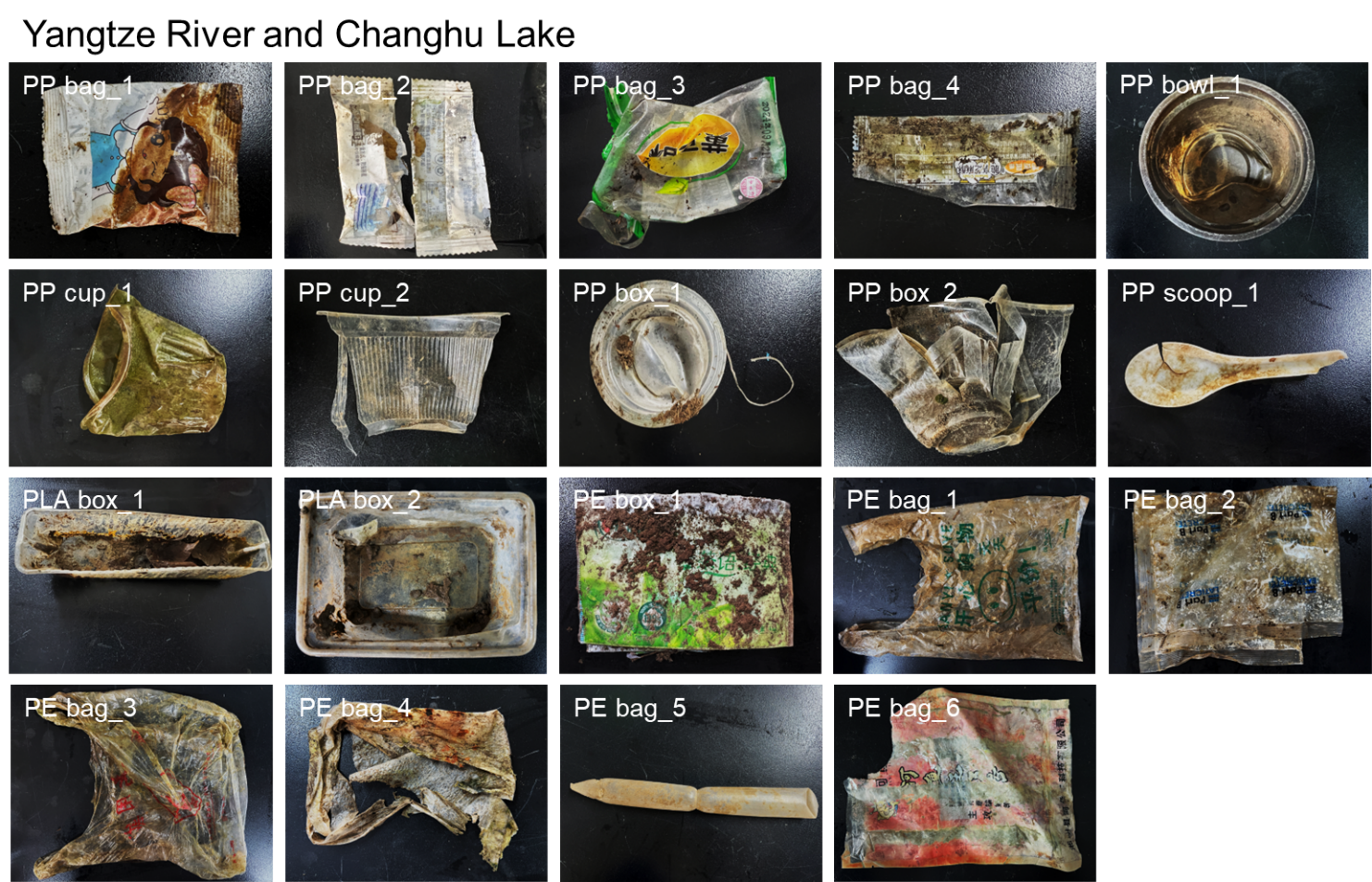

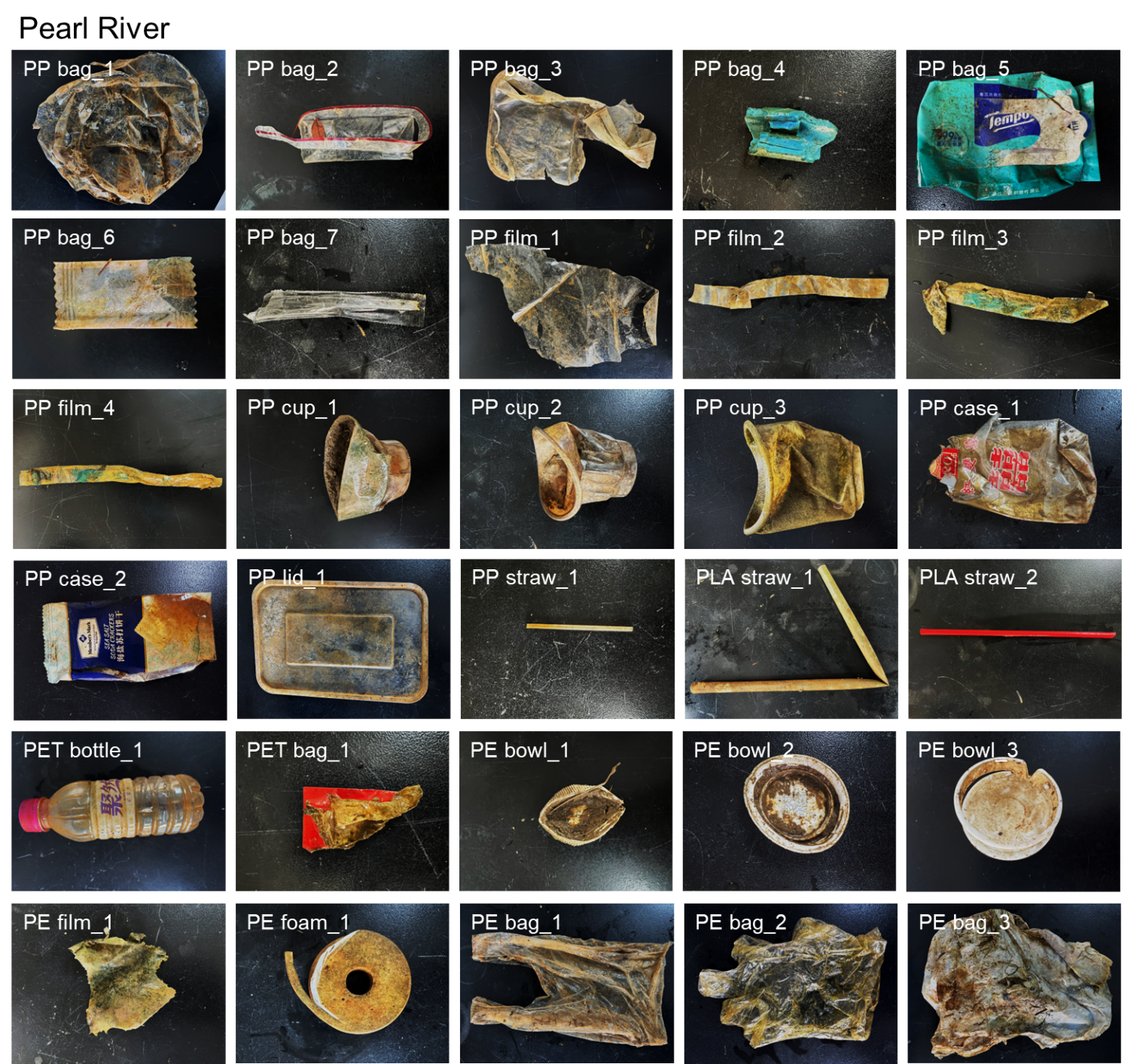

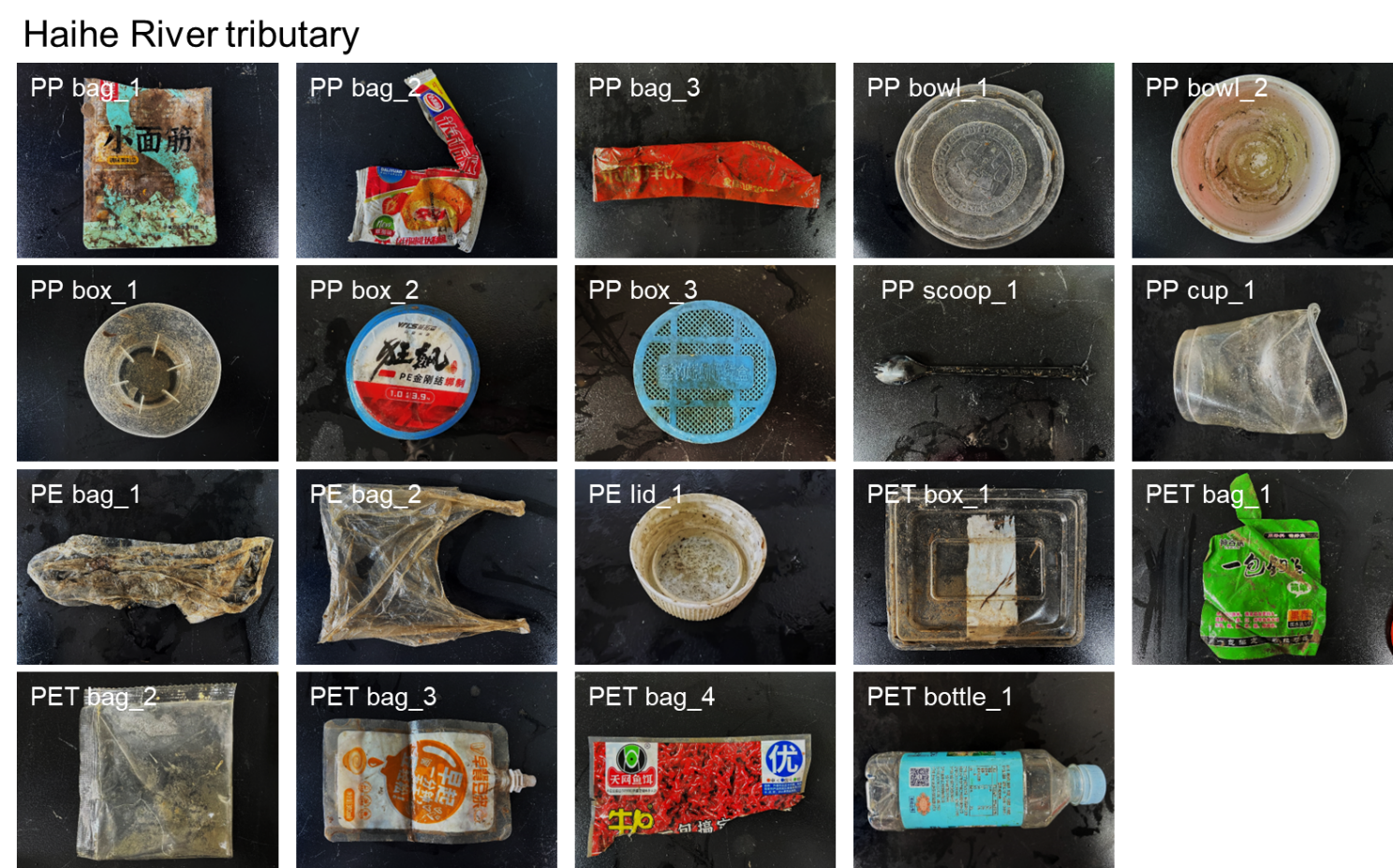

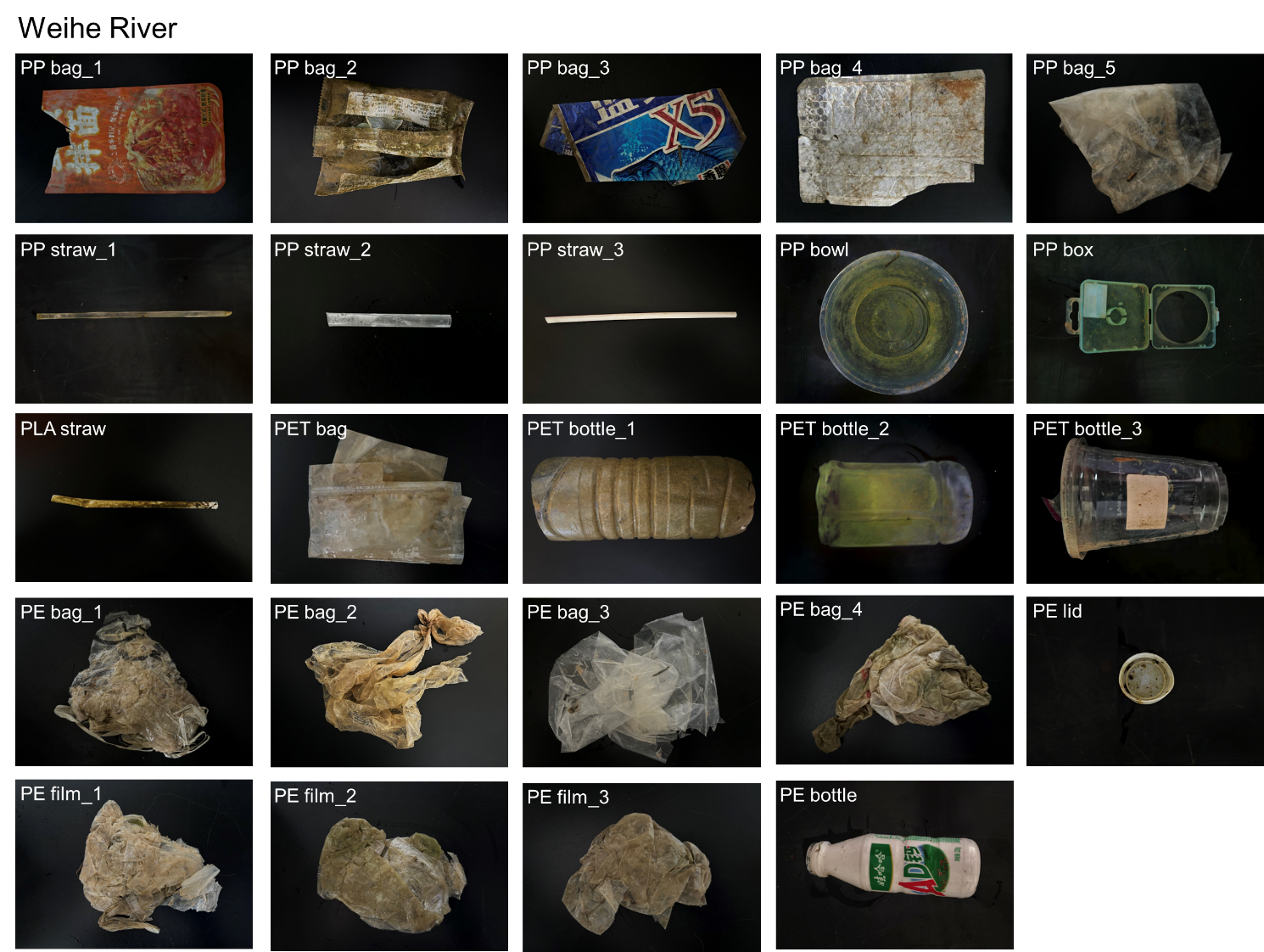


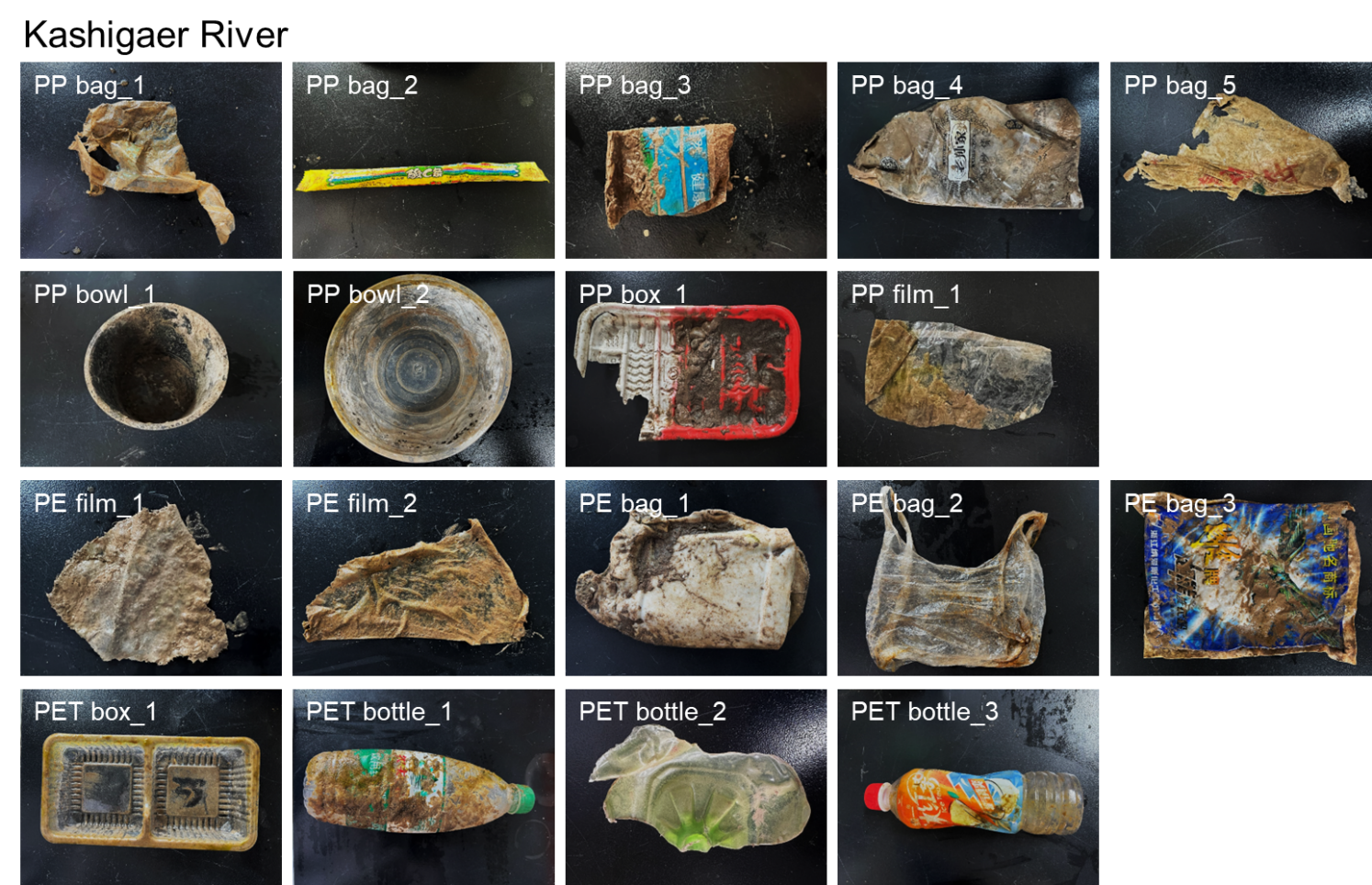


# **Fig. S2.** Digital images **of plastic samples collected from 6 important river systems in China.**


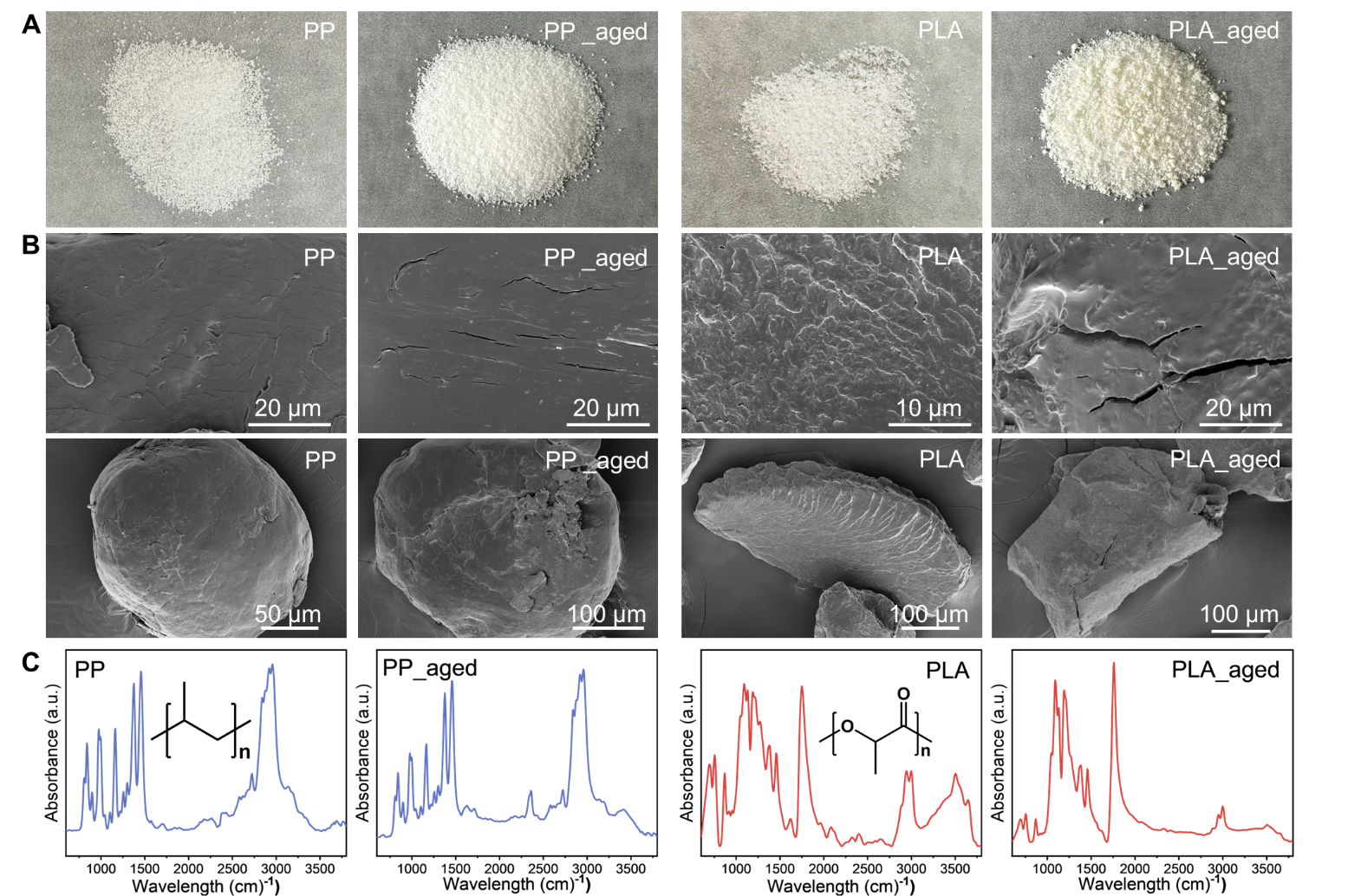


# **Fig. S3. Physicochemical characterization of virgin and** photoaged **MPs.** (A) Digital images, (B) SEM images, and (C) FTIR spectra. The terms “PP”, “PLA”, “PP_aged” and “PLA_aged” denote virgin PP, virgin PLA, photoaged PP, photoaged PLA, respectively. The similar meanings apply to the following figures and tables.


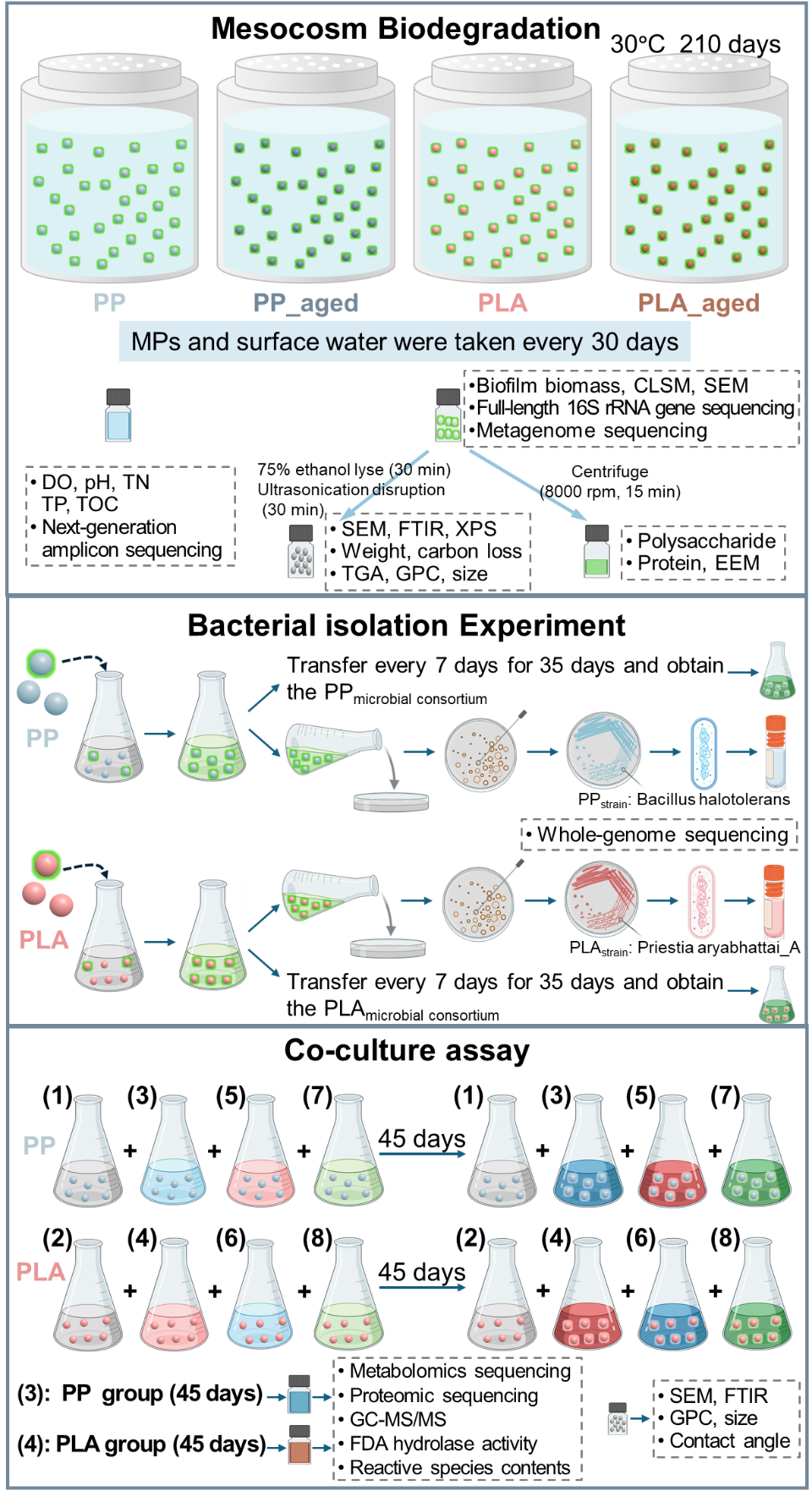


# **Fig. S4. Schematic illustration of the experimental design and analytical workflow.** The schematic outlines the experimental setup for mesocosm incubation, bacterial isolation, and co-culture assay. The treatment groups, sampling intervals, and analysis methods for each experiment are described.


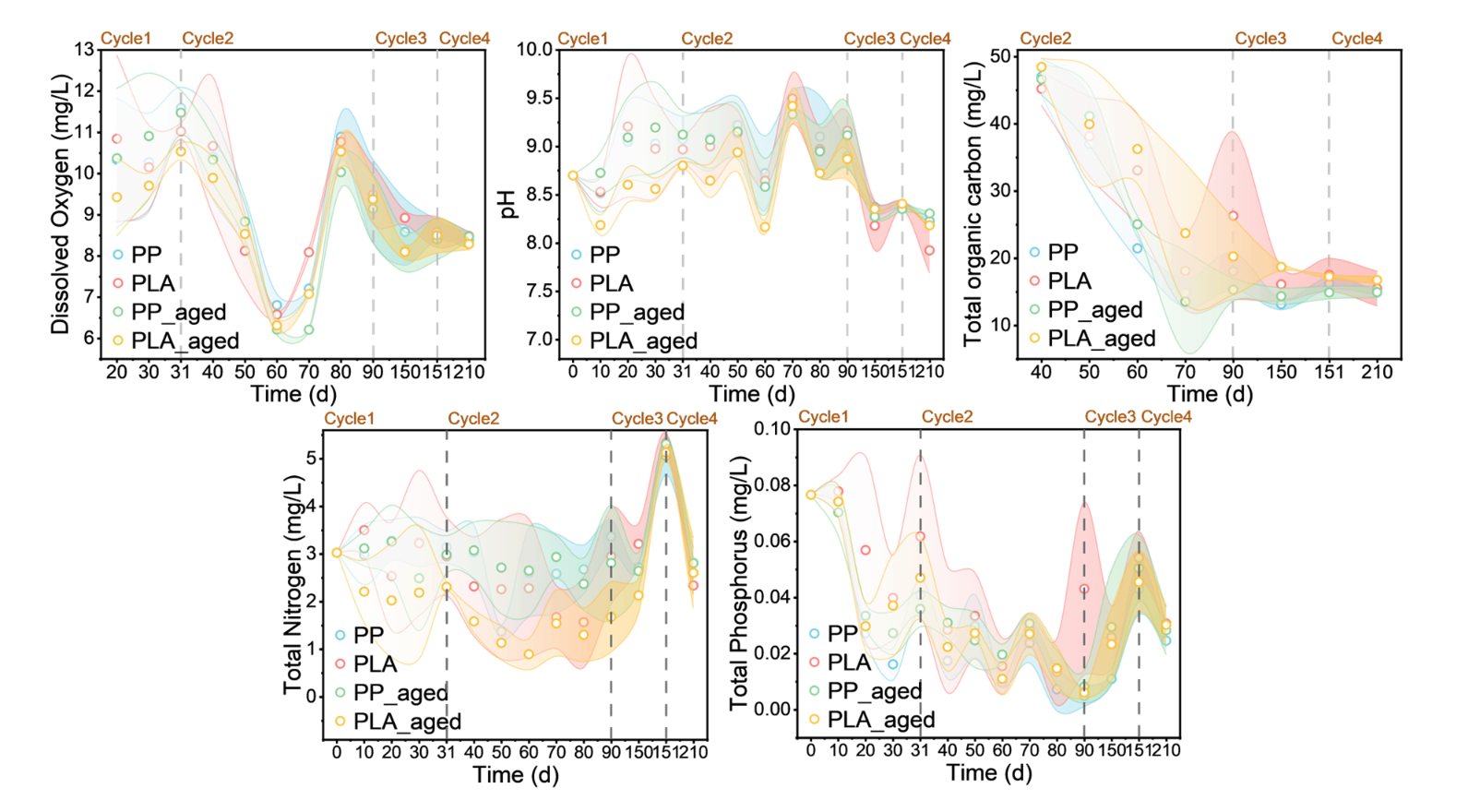


# Fig. S5. Changes in water parameters during mesocosm incubation. Error bars represent the standard deviation of three replicates of mesocosm experiment (n = 3).


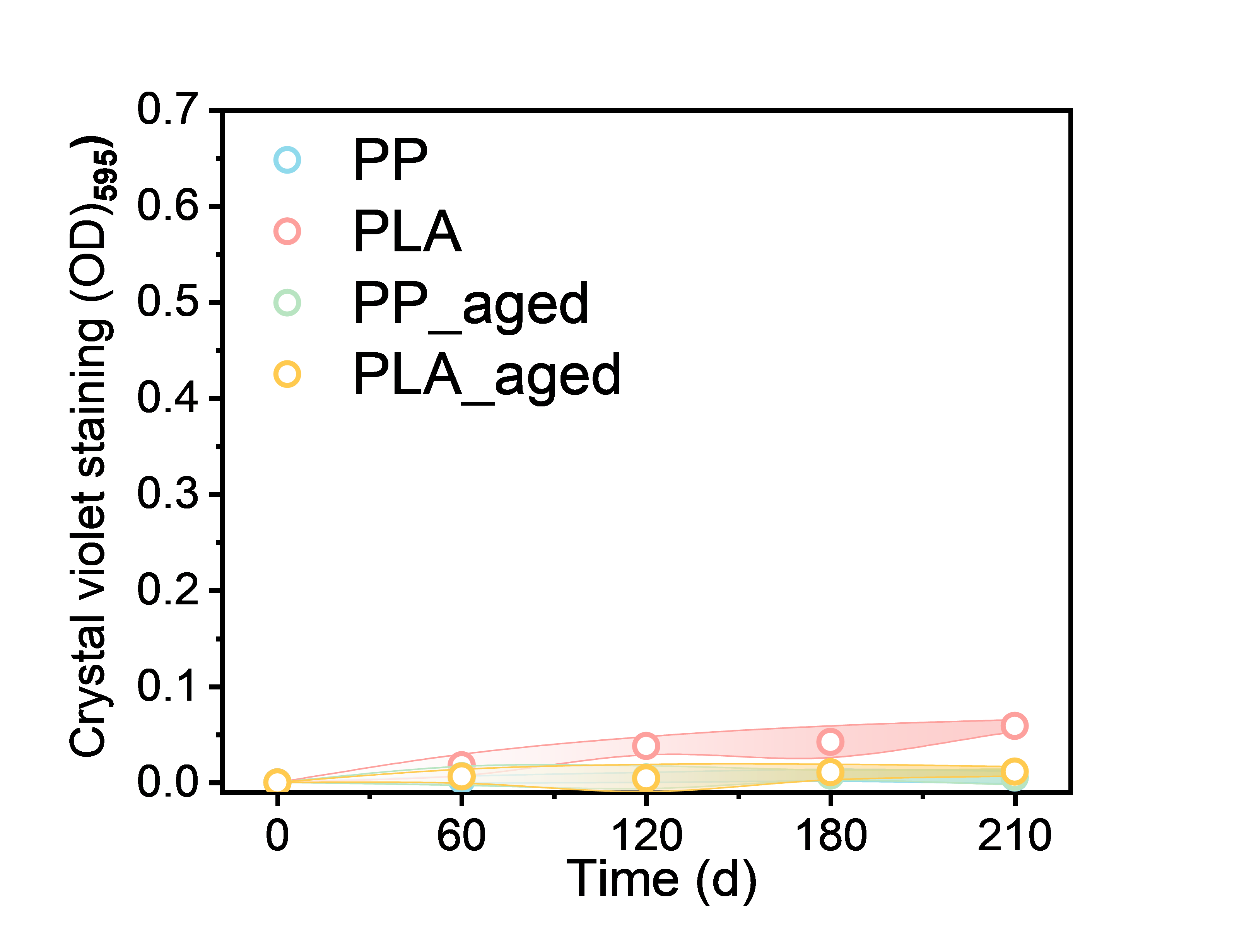


# Fig. S6. Residual biofilm biomass of virgin and photoaged MPs after mesocosm incubation and biofilm removal. Error bar represents the standard deviation from three replicate biofilm removal samples (n = 3).


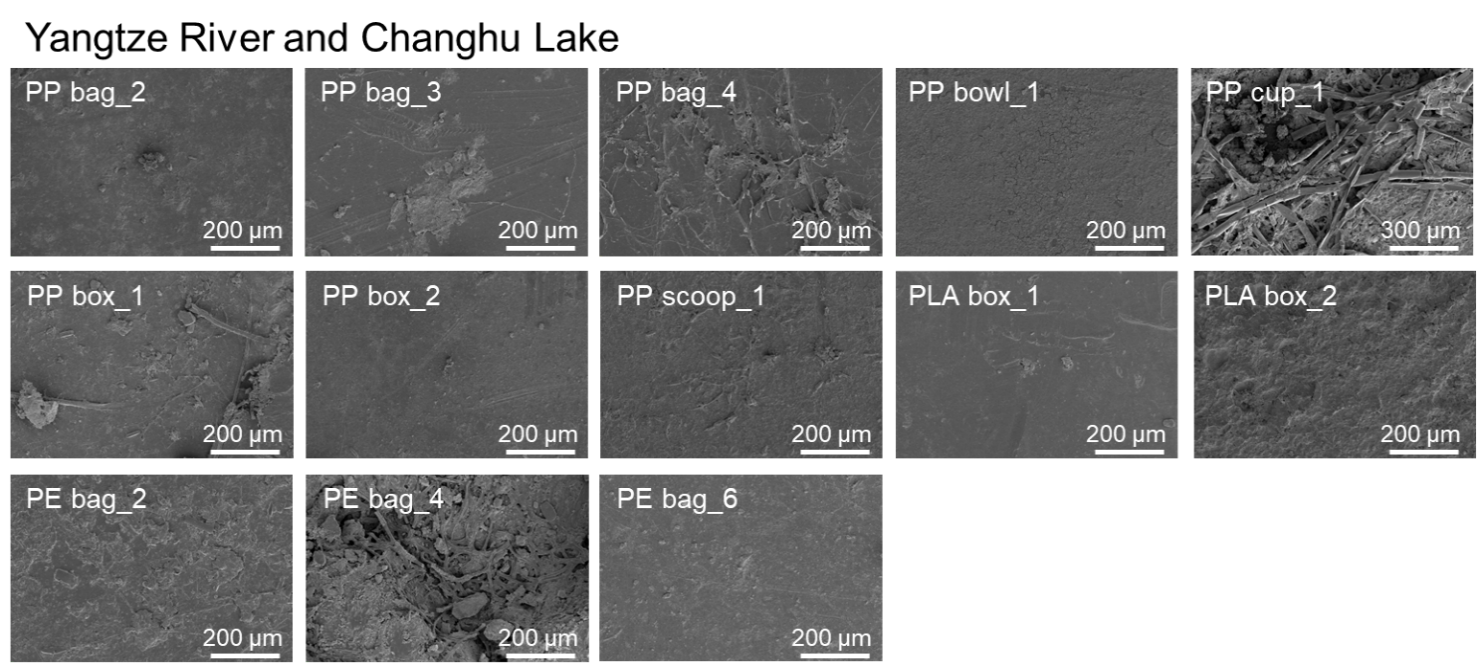


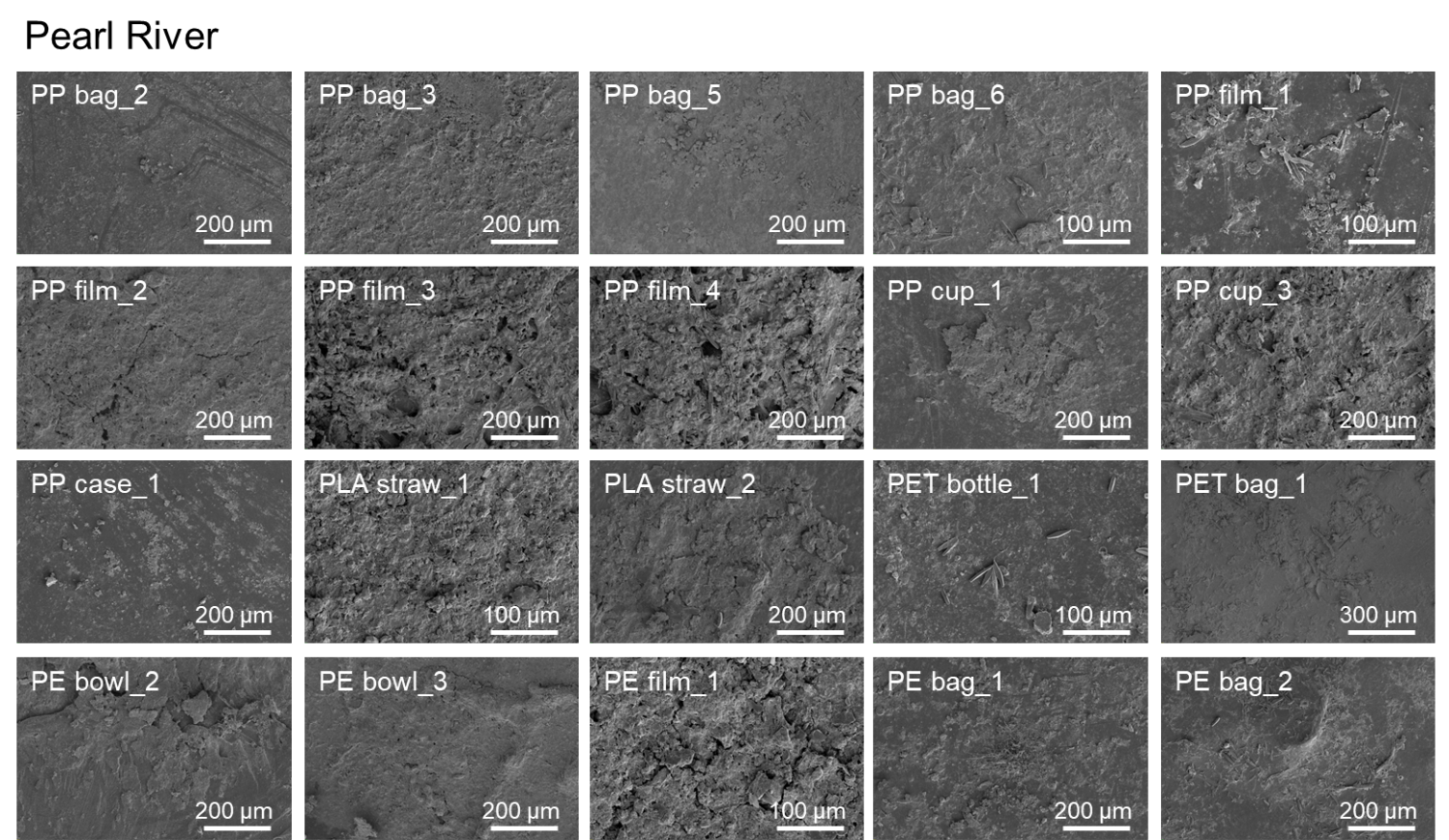


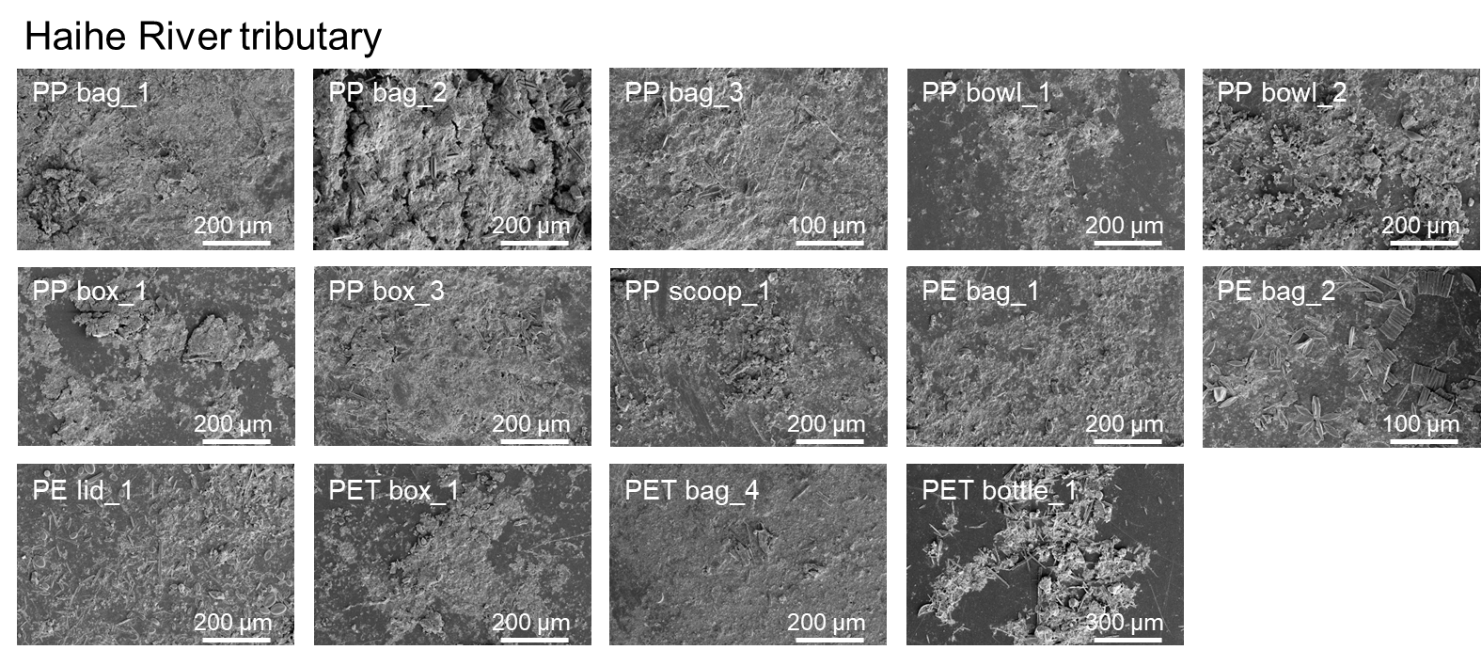


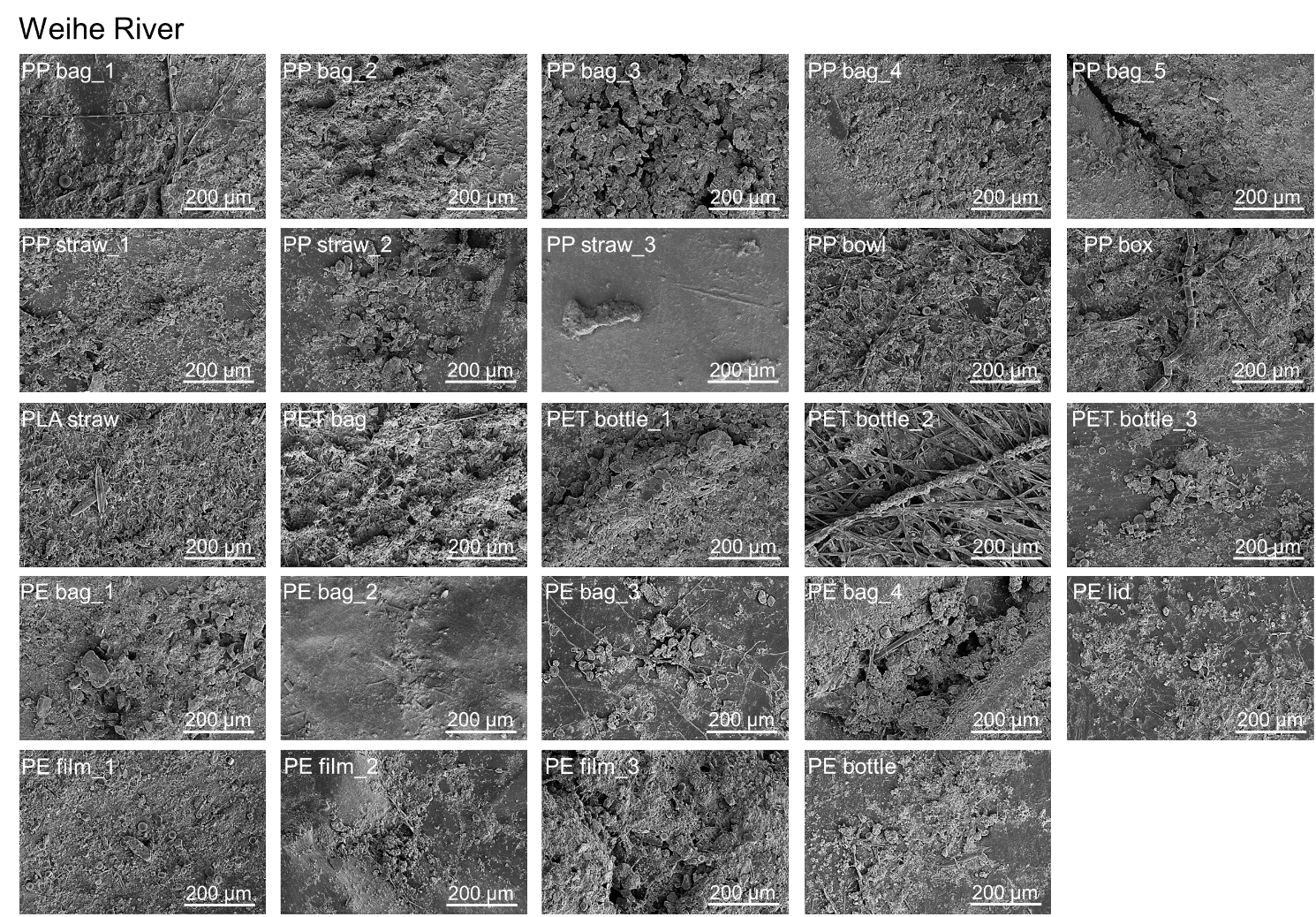


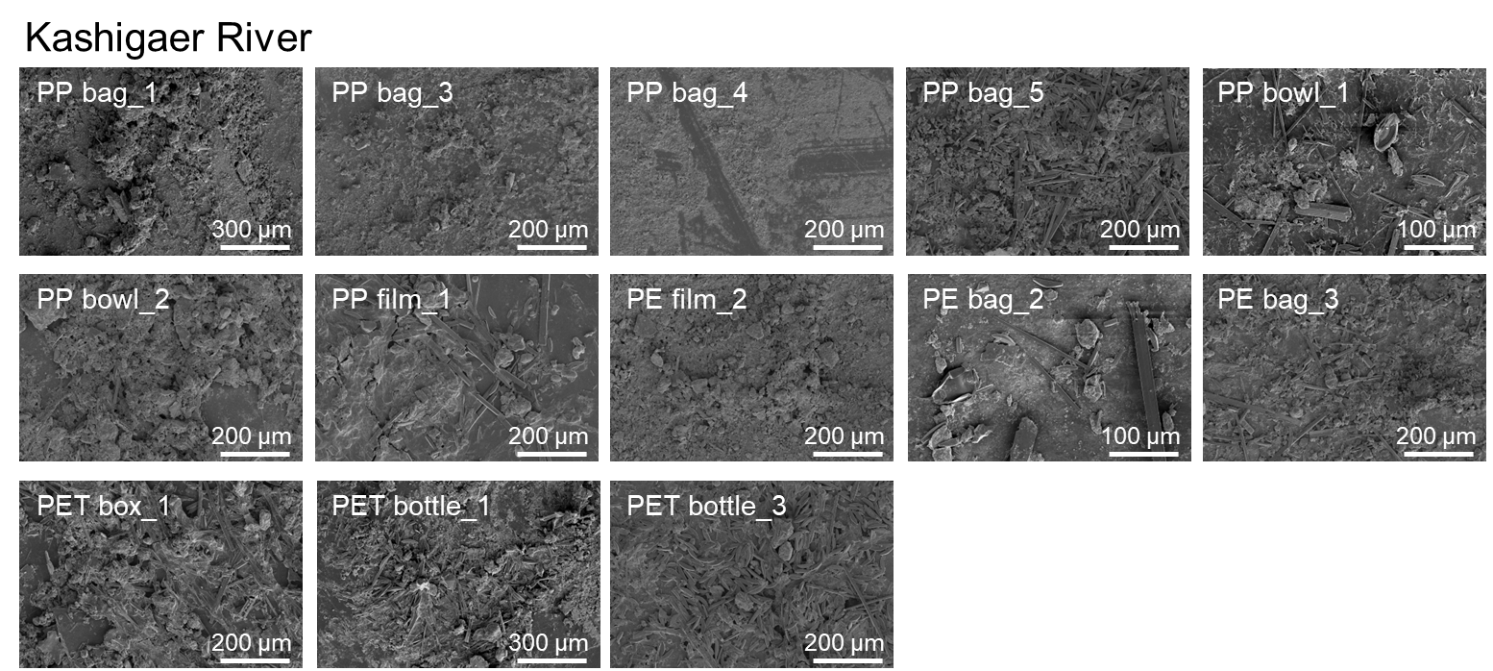


# **Fig. S7. SEM images of biofilm-formed plastic samples in 6 important river systems in China.** Diverse microbial morphotypes, primarily filamentous, spherical, and rod-shaped structures colonized plastic surfaces.


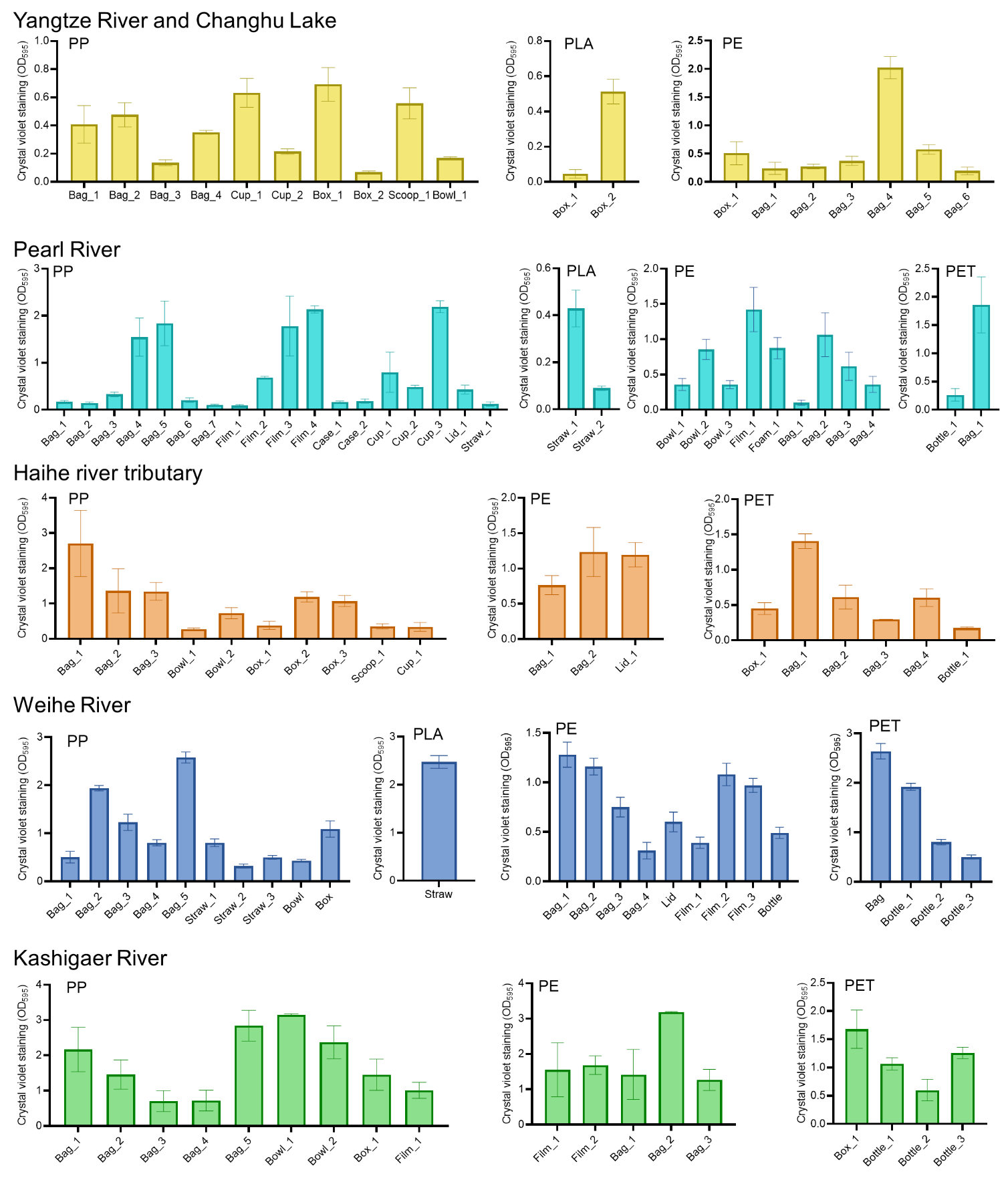


# **Fig. S8.** Biofilm biomass **of plastic samples in 6 important river systems in China.** Error bars represent the standard deviation of triplicate measurements from each plastic sample.


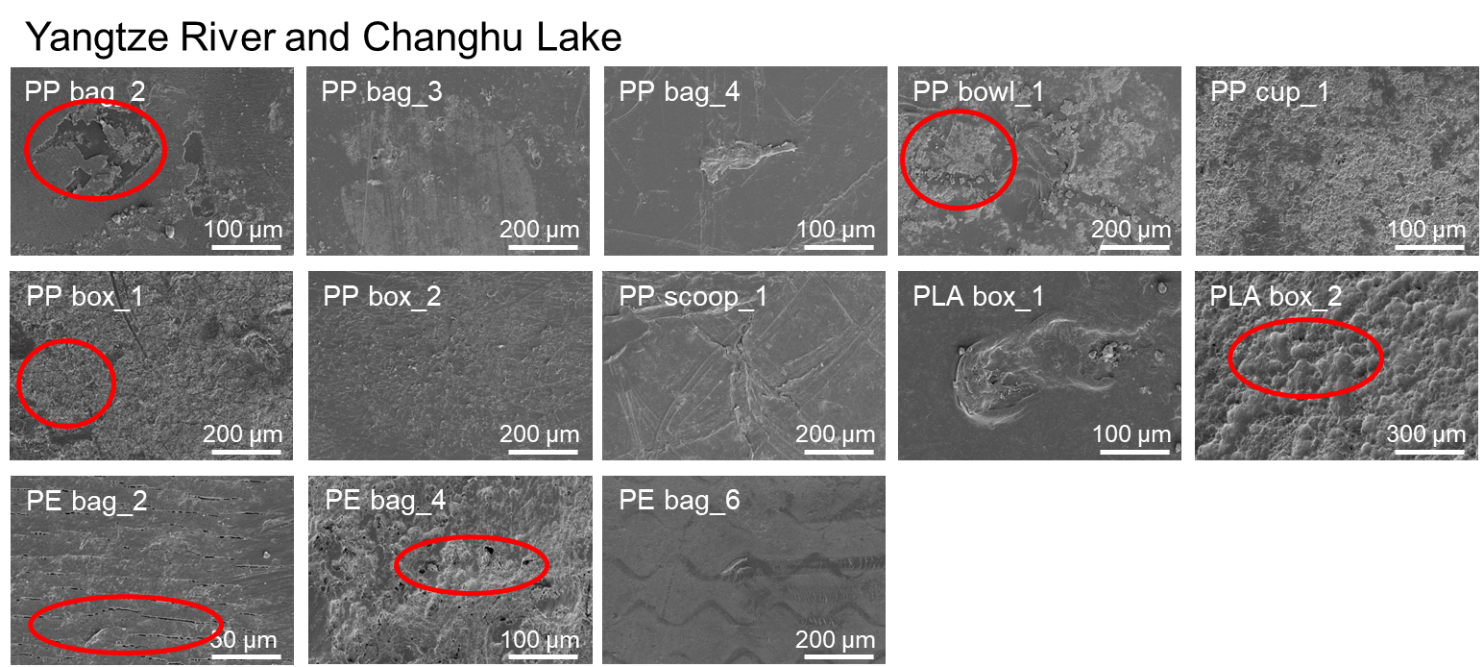

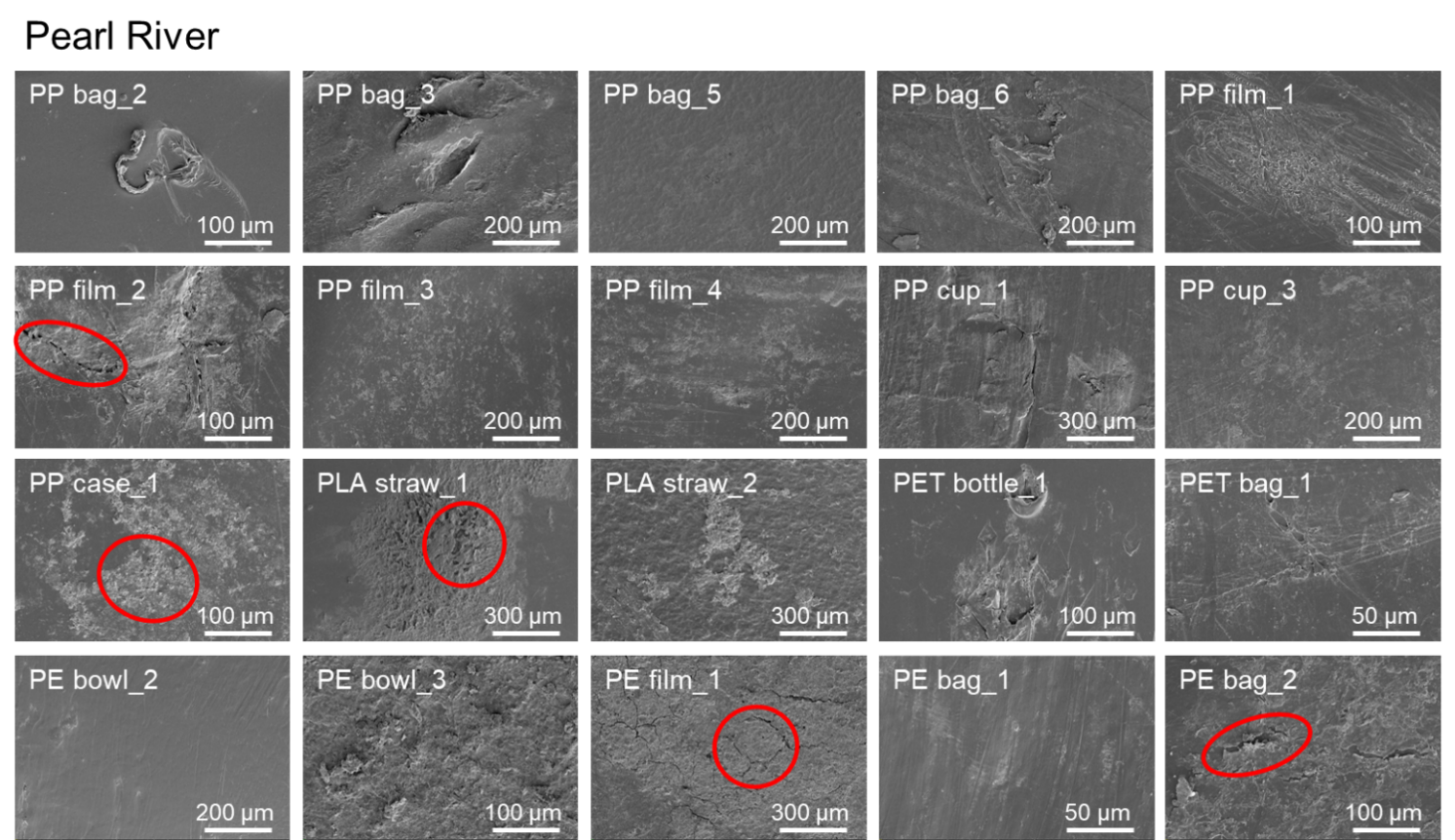


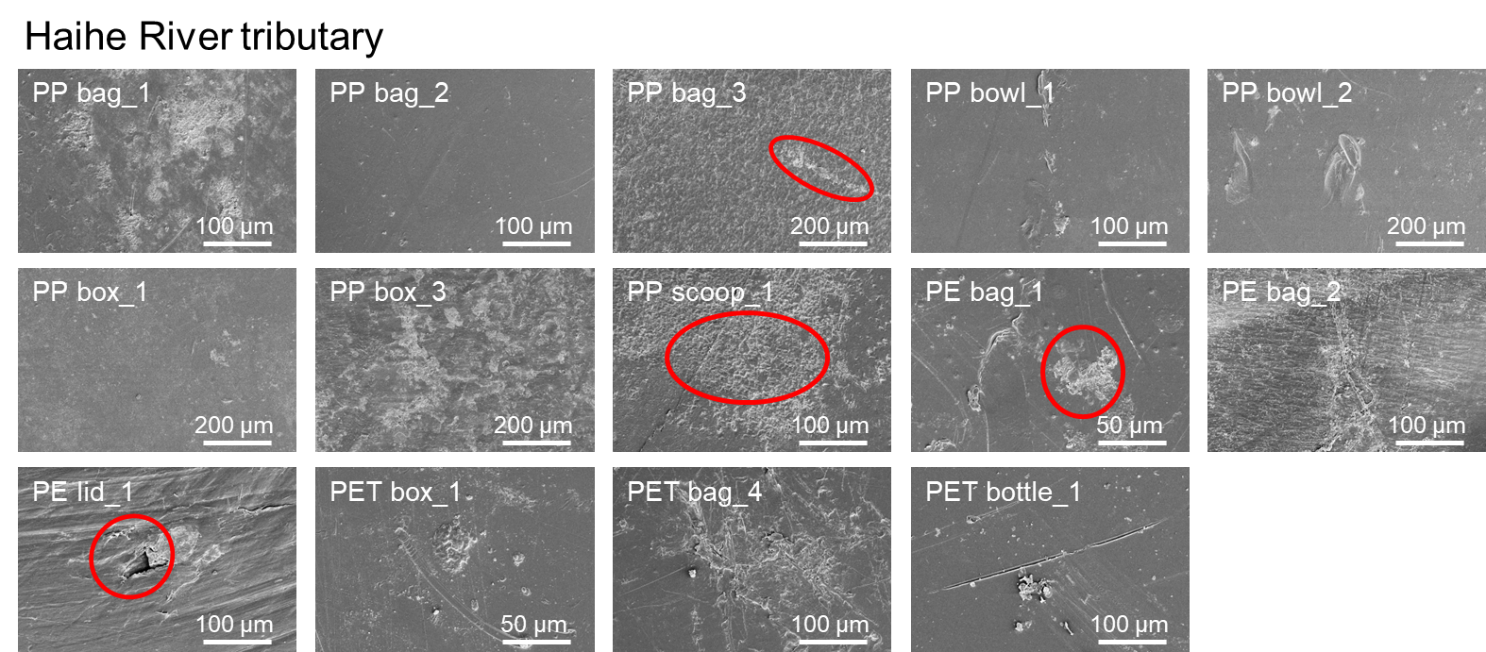


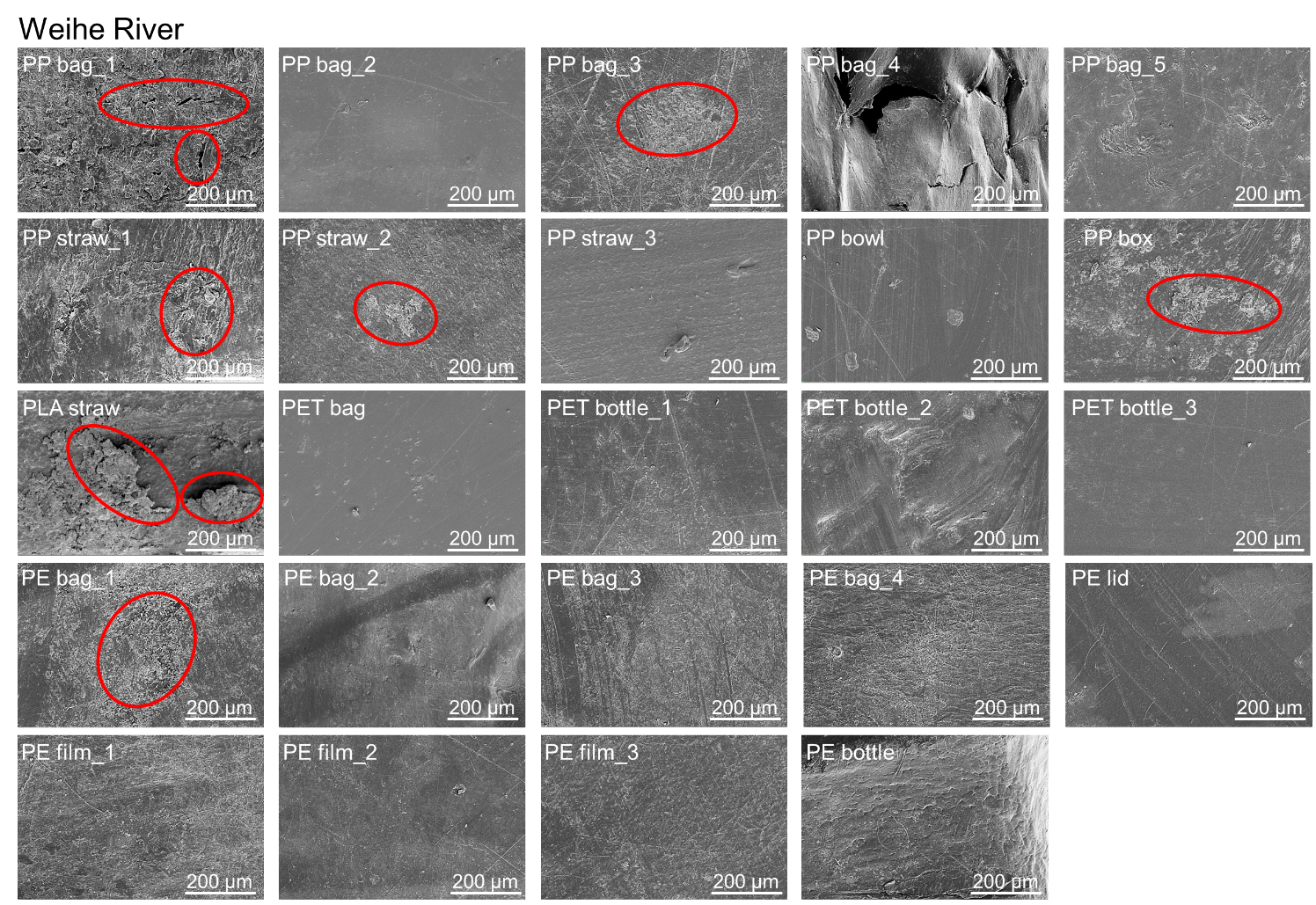


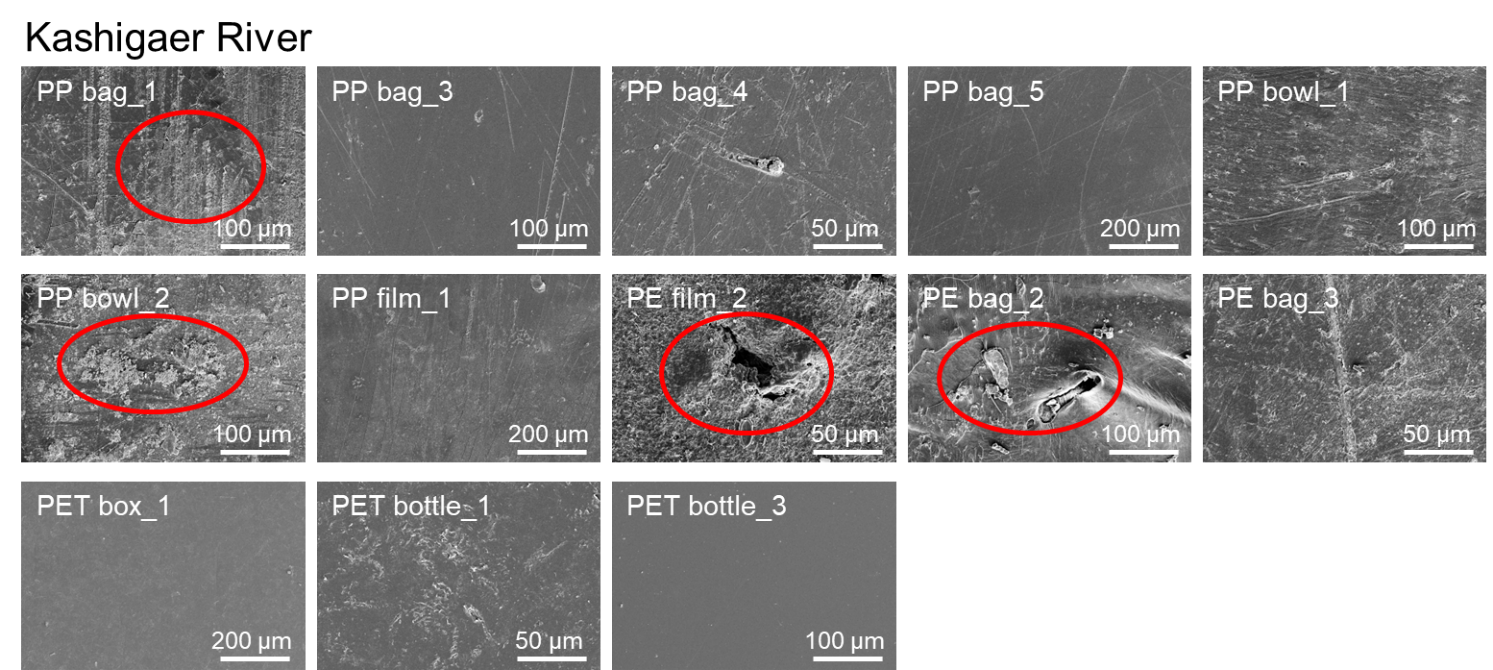


# **Fig. S9.** SEM images of biofilm-removed plastic samples **in 6 important river systems in China**. The bioerosions are marked in red circles.


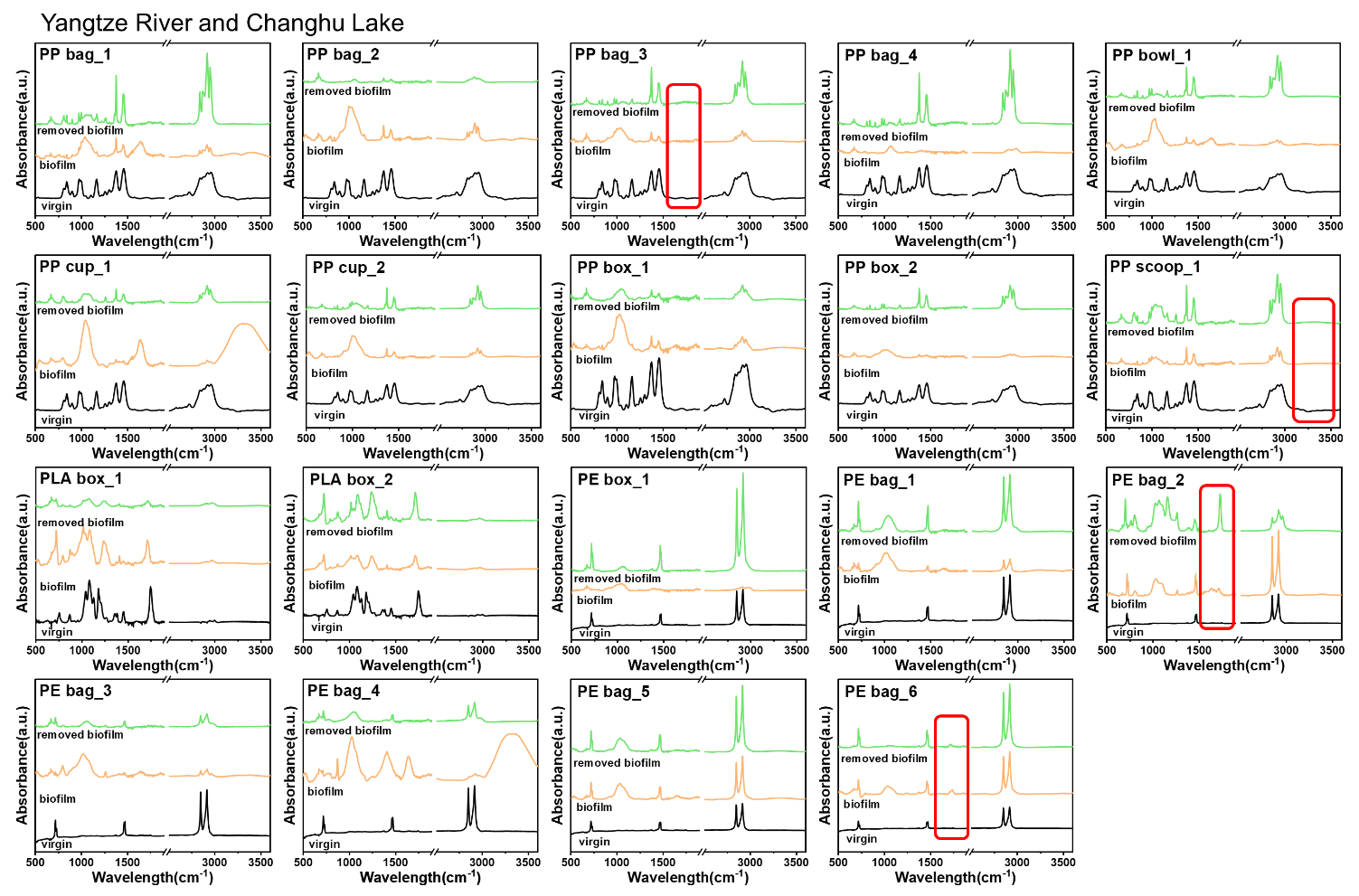

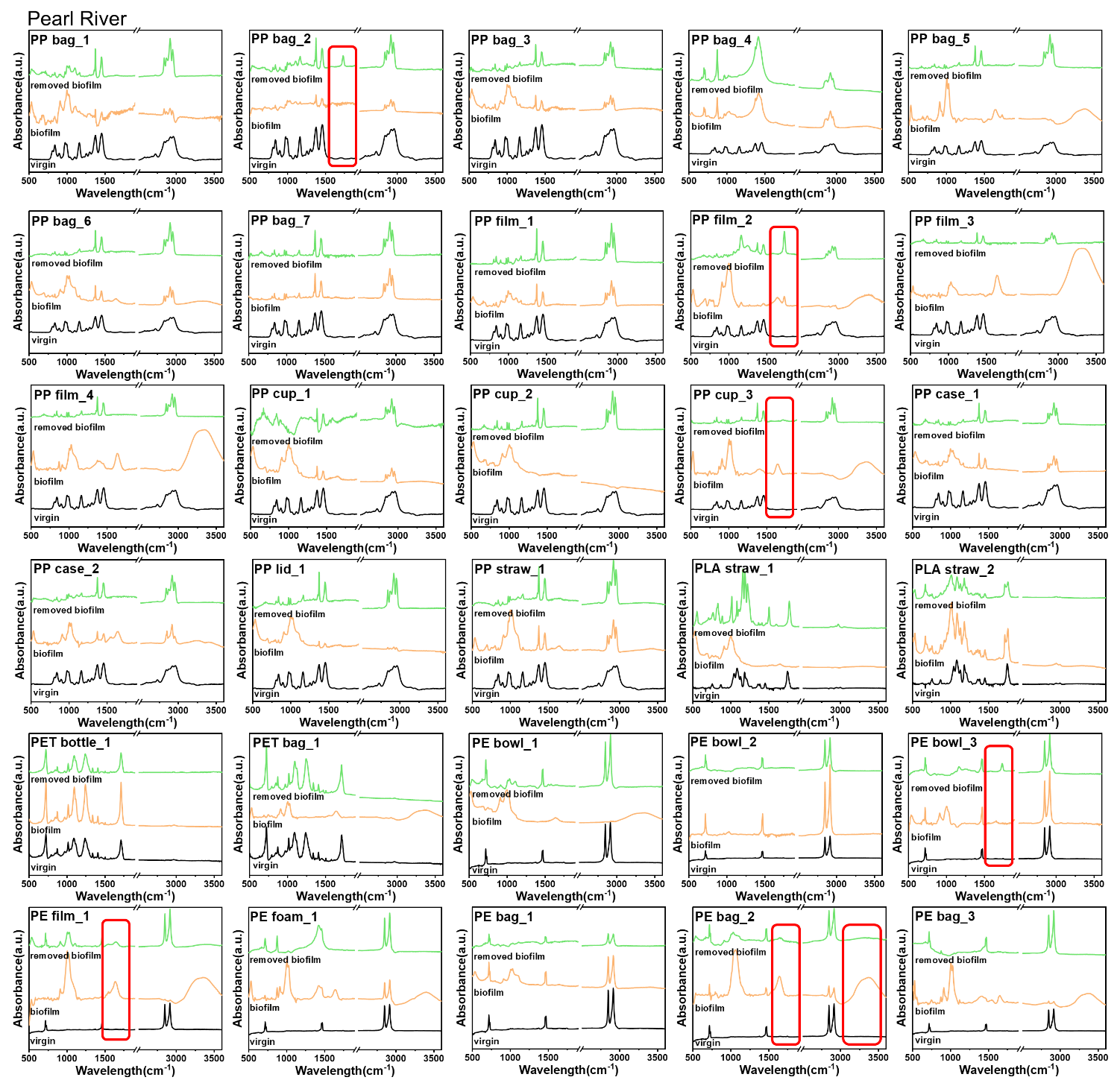

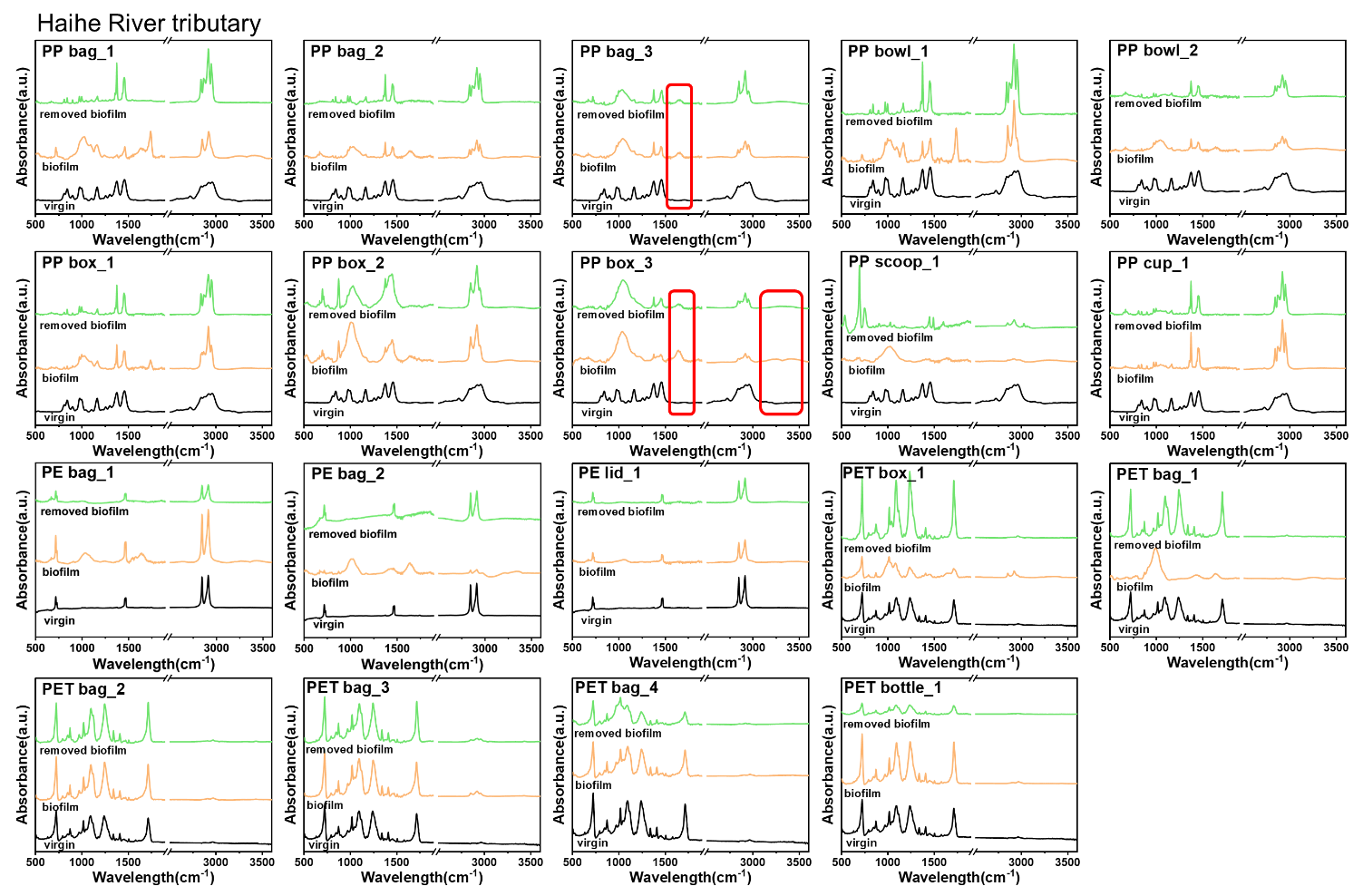

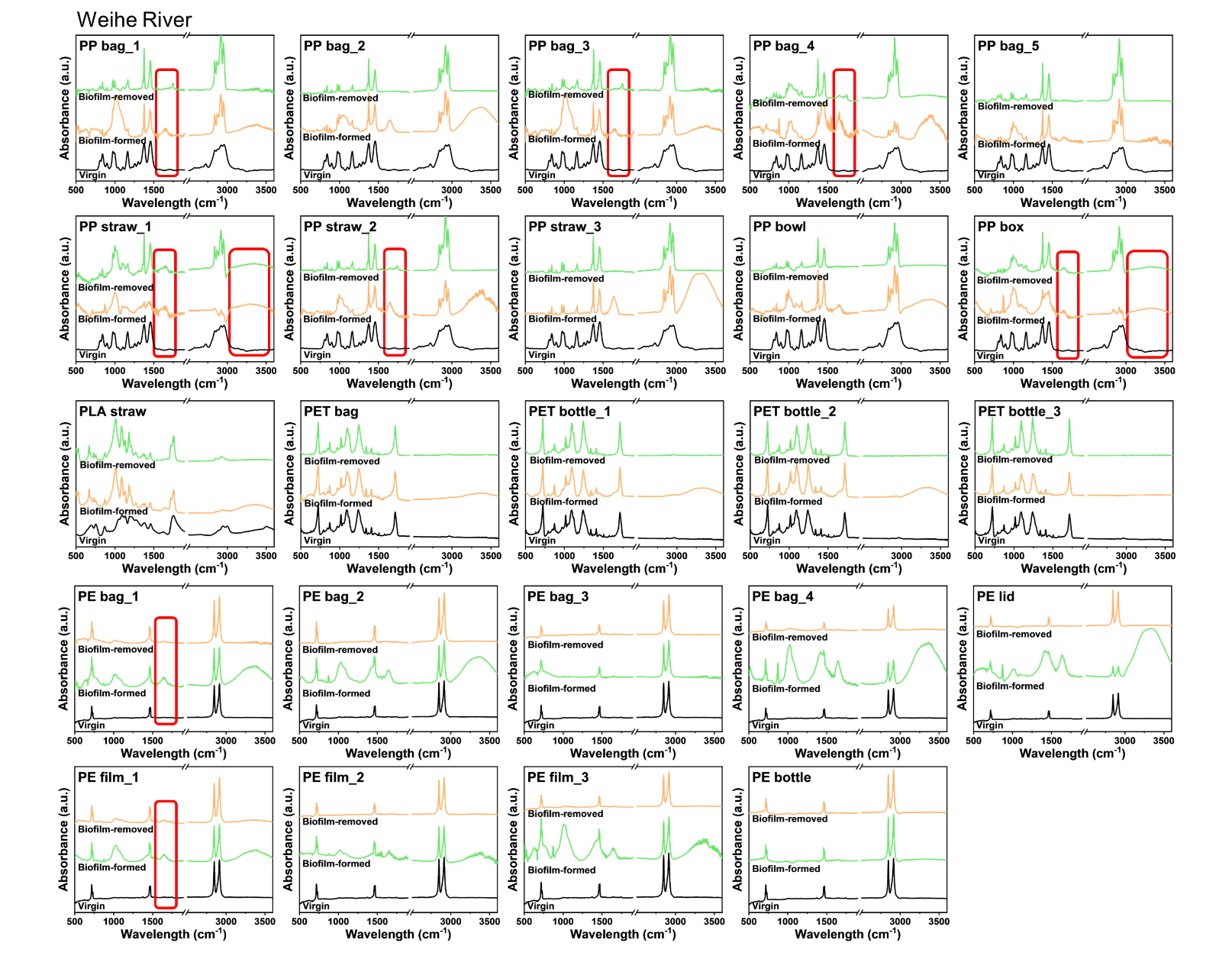


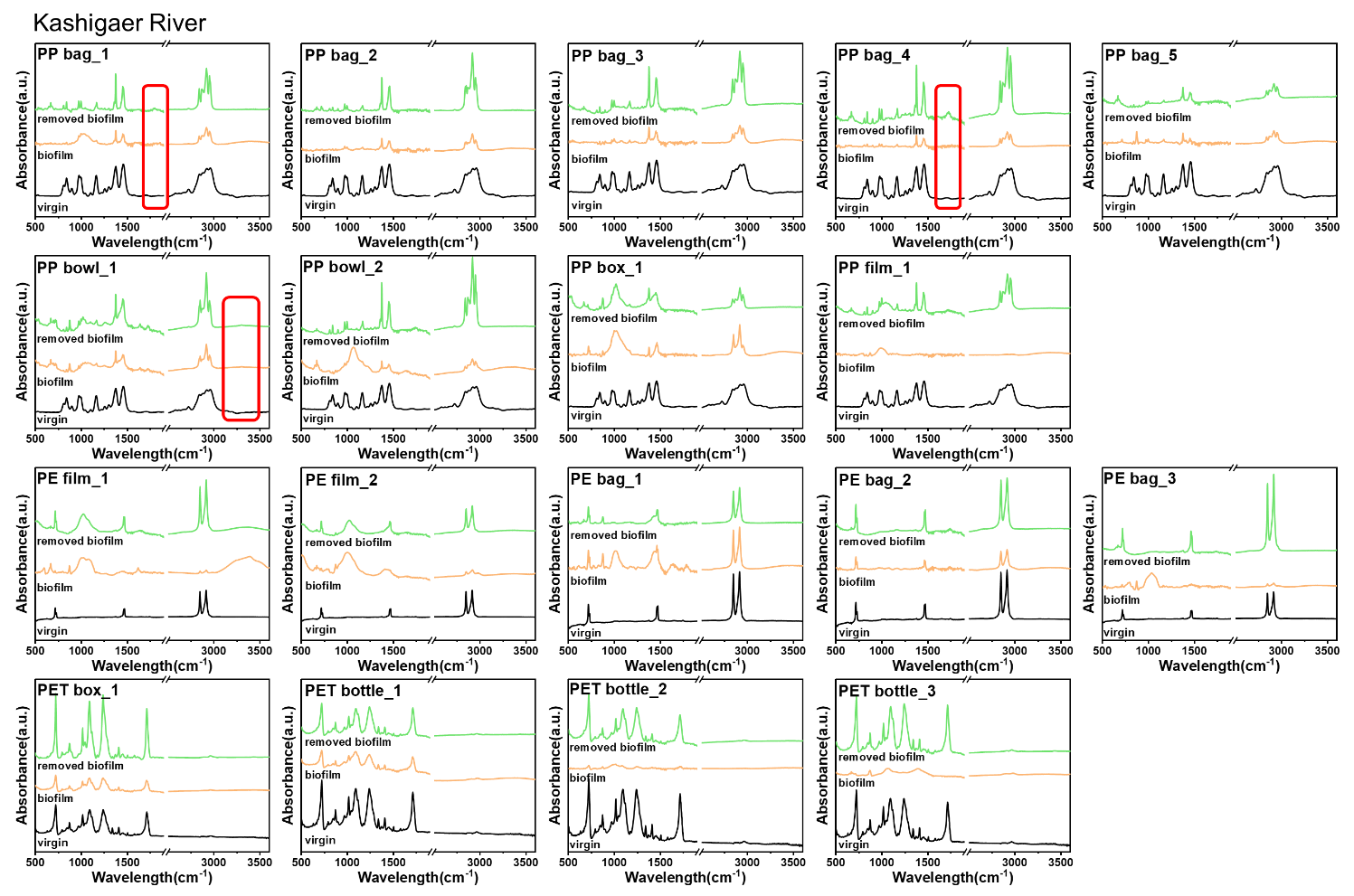


# **Fig. S10.** FTIR spectra **of virgin, biofilm-formed, and biofilm-removed plastic samples in 6 important river systems in China.** The oxygen-containing functional groups with obvious changes are marked in red circles.


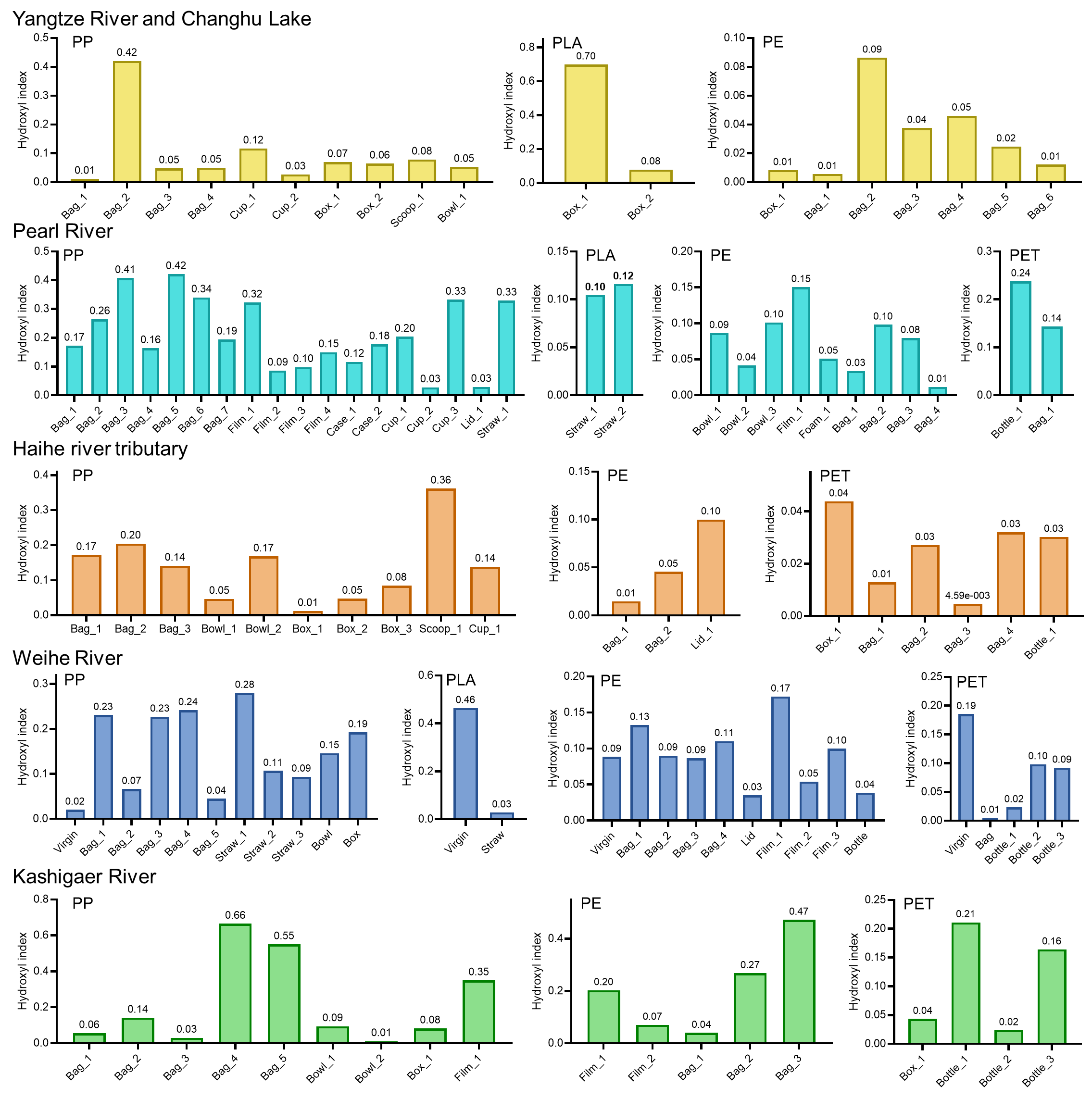


# **Fig. S11.** Hydroxyl index of biofilm-removed plastic samples in **6 important river systems in China**. The data were obtained from the calculation using the maximum intensity of the hydroxyl group (I_3348–3369_ for PP, PET, and PE and I_3502_ for PLA) relative to the methylene group (I_977_ for PP, I_971_ for PET, I_717_ for PE, and I_1452_ for PLA) of FTIR data.


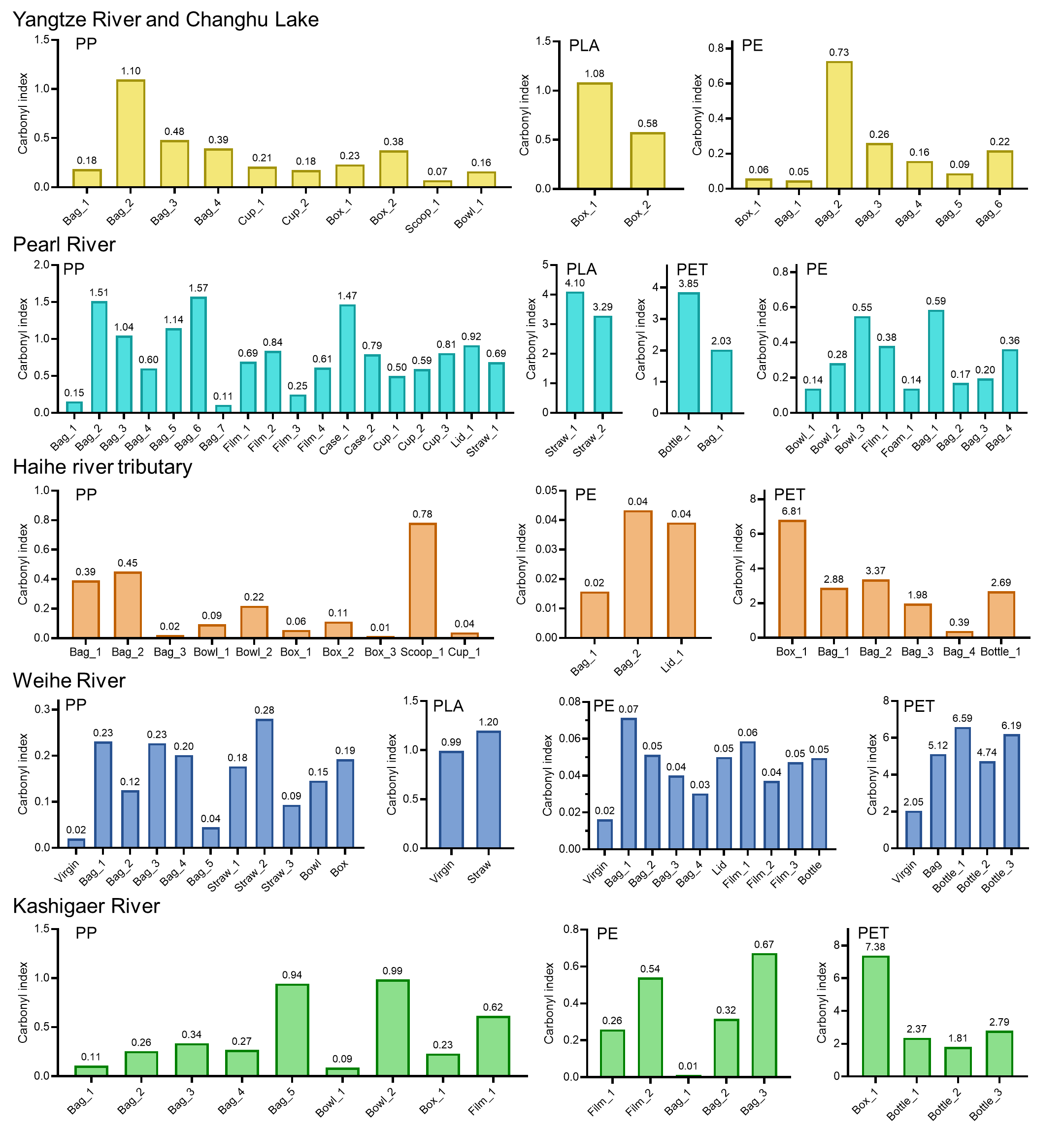


# **Fig. S**12. Carbonyl index of biofilm-removed plastic samples in **6 important river systems in China**. The data were obtained from the calculation using the maximum intensity of the carbonyl group (I_1714_ for PP, PET, and PE and I_1760_ for PLA) relative to the methylene group (I_977_ for PP, I_971_ for PET, I_1471_ for PE, and I_1452_ for PLA) of FTIR data.


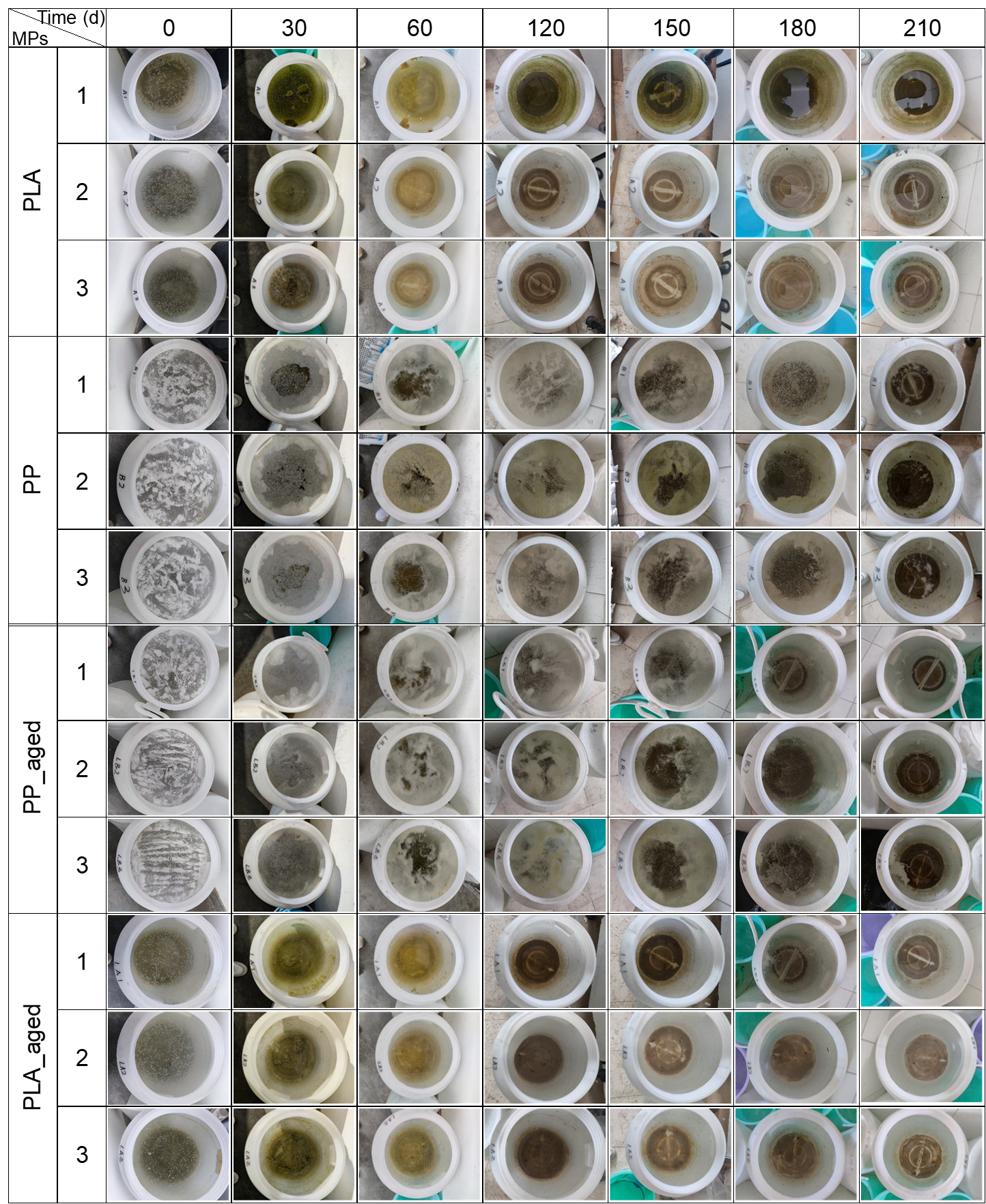


# Fig. S13. Digital images of the progression of mesocosm incubation.


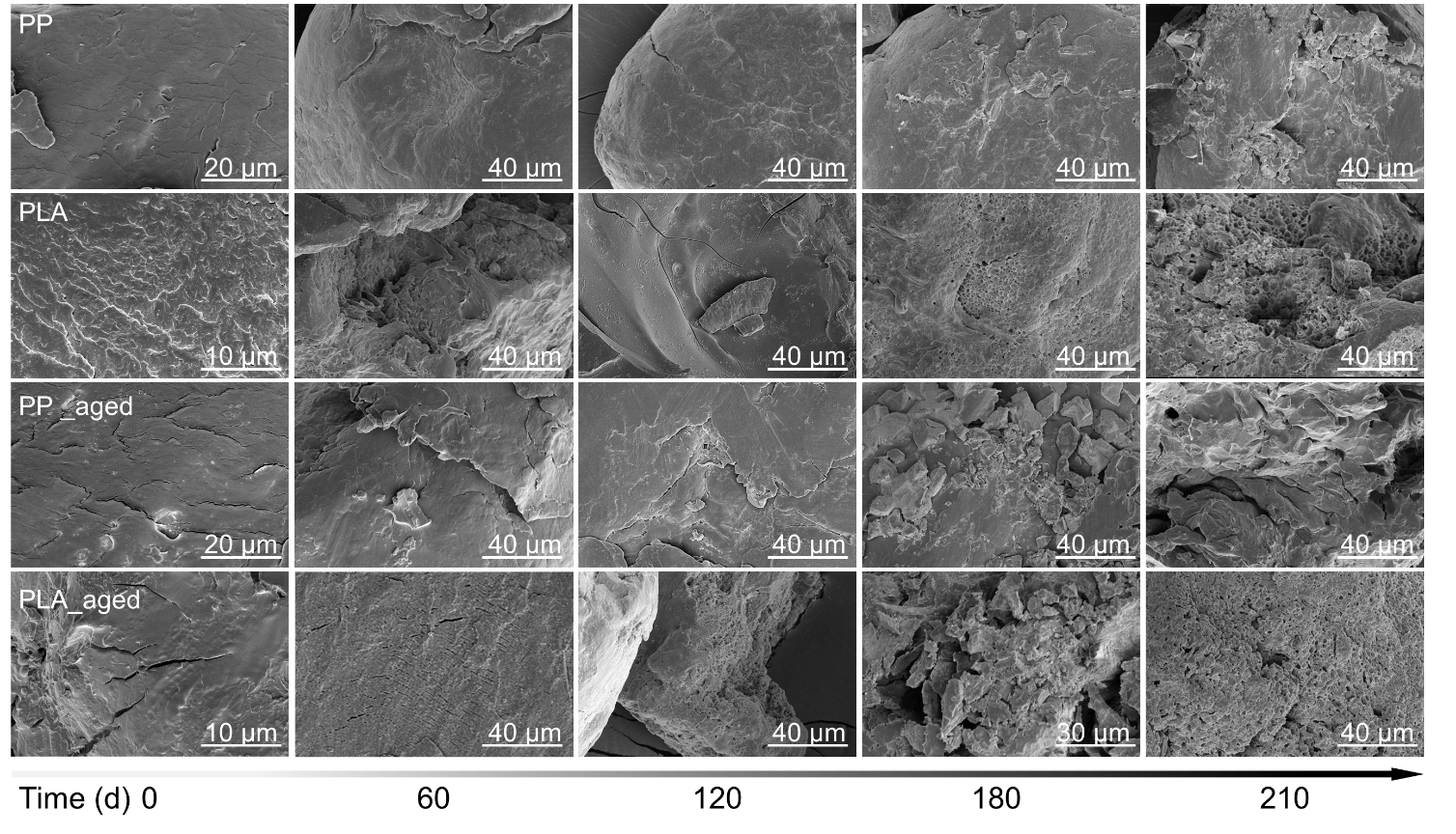


# Fig. S14. SEM images of virgin and photoaged MPs during mesocosm incubation.

#
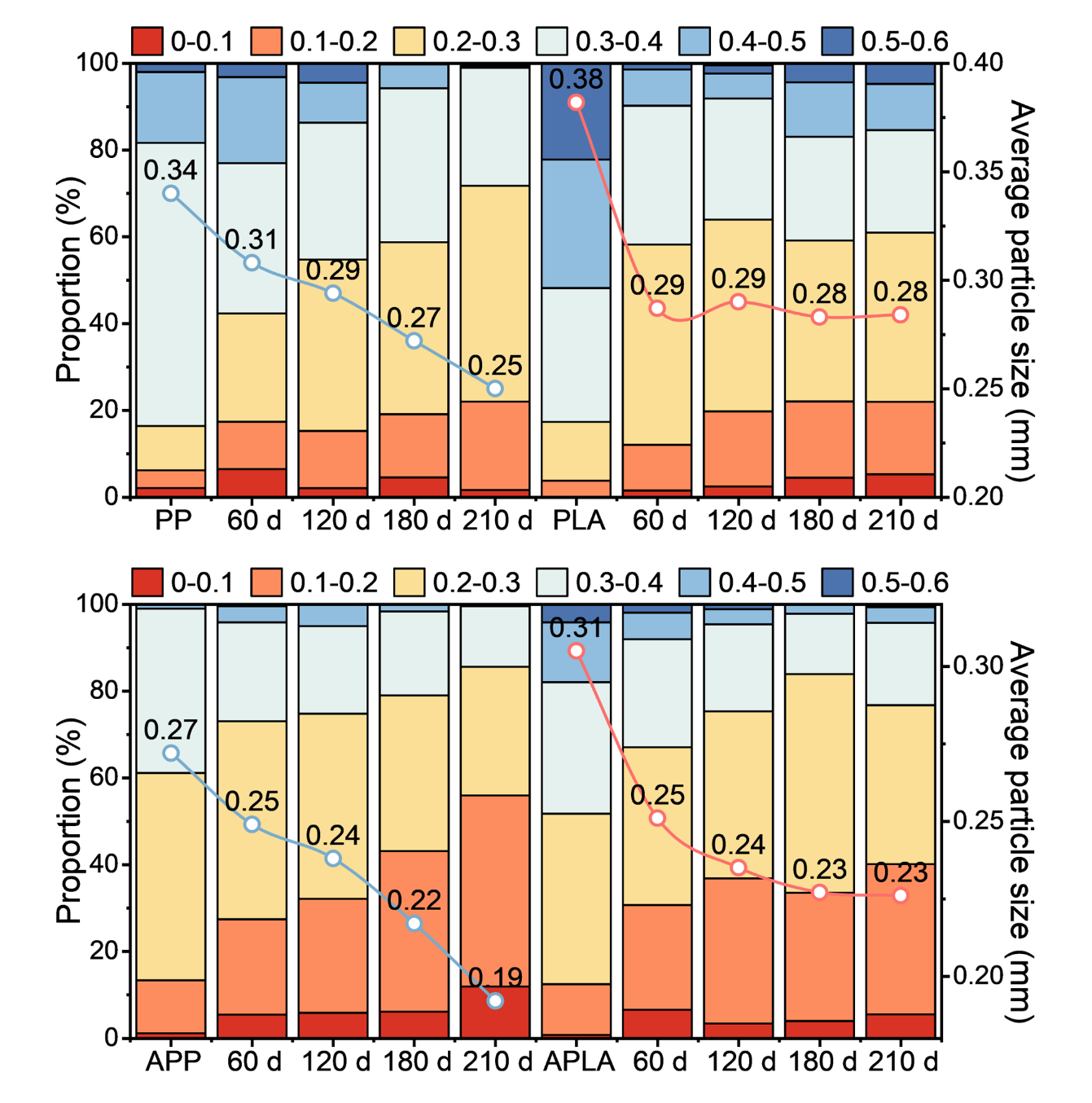


# Fig. S15. Particle size distribution and average particle size of virgin and photoaged MPs during mesocosm incubation. The terms “APP” and “APLA” denote photoaged PP and PLA, respectively. The similar meanings apply to the following figures and tables.


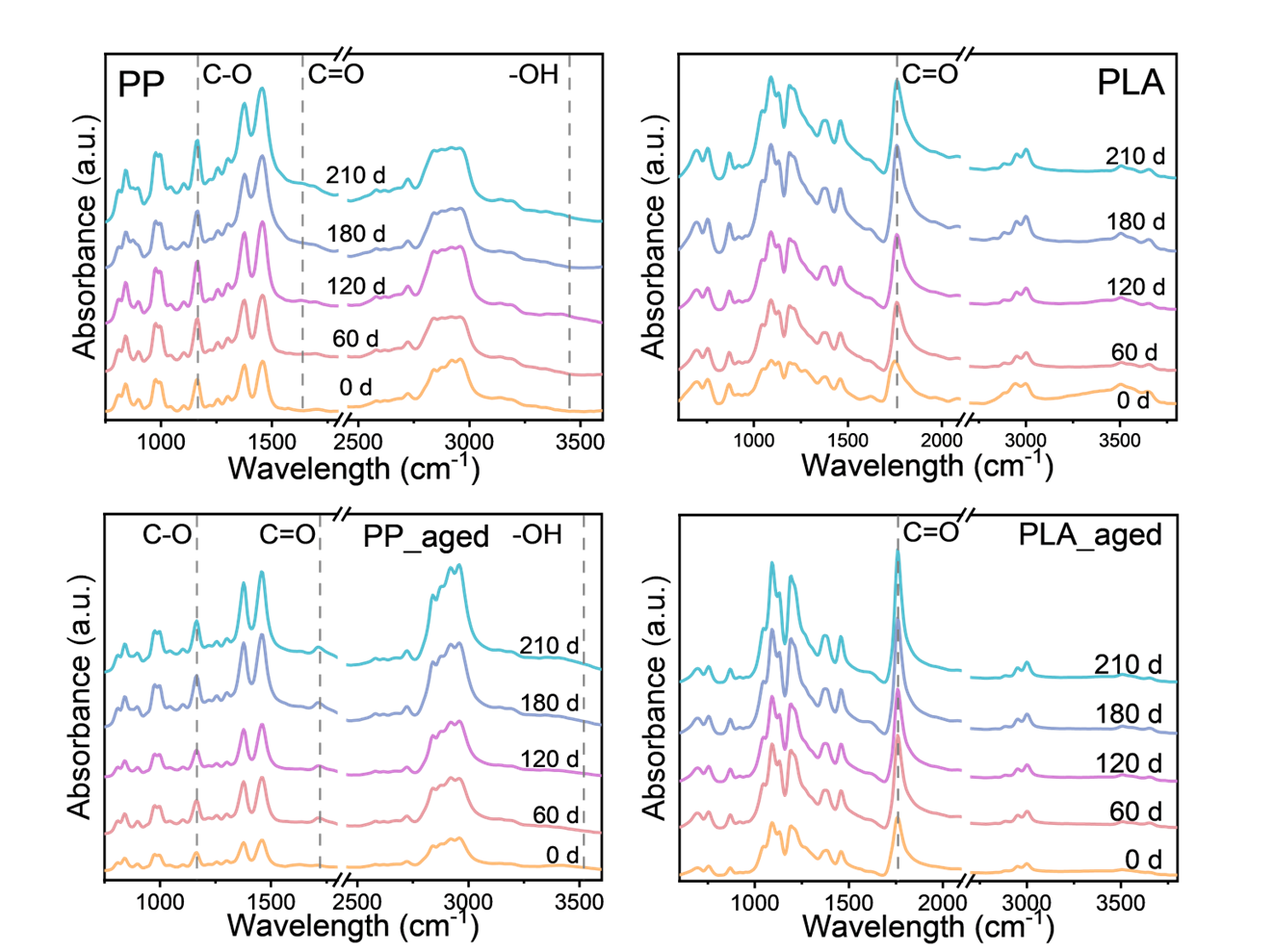


# Fig. S16. FTIR spectra of virgin and photoaged MPs during mesocosm incubation.


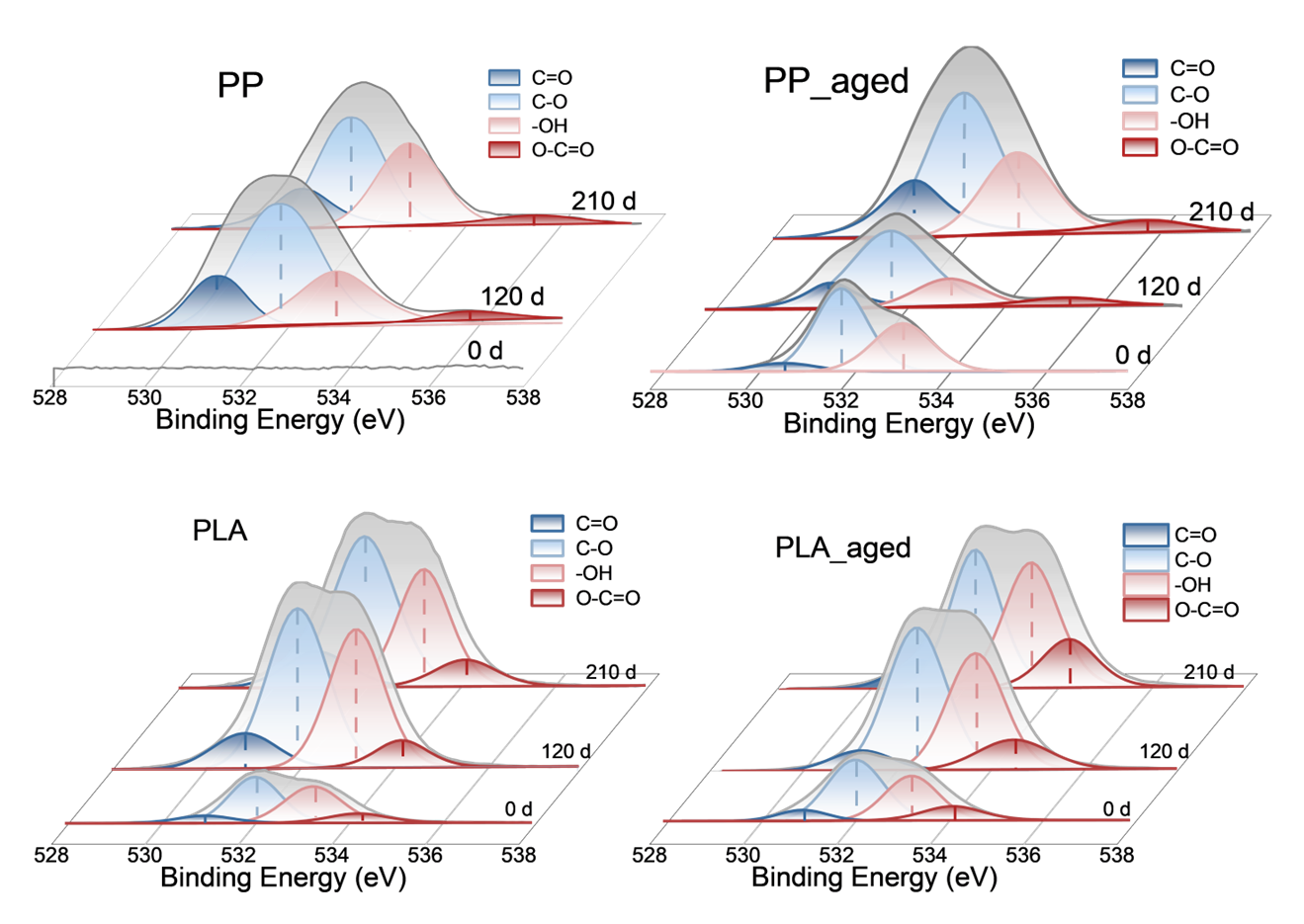


# Fig. S17. XPS spectra of virgin and photoaged MPs during mesocosm incubation.

# Fig. S18. O/C ratio of virgin and photoaged MPs during mesocosm incubation.

**
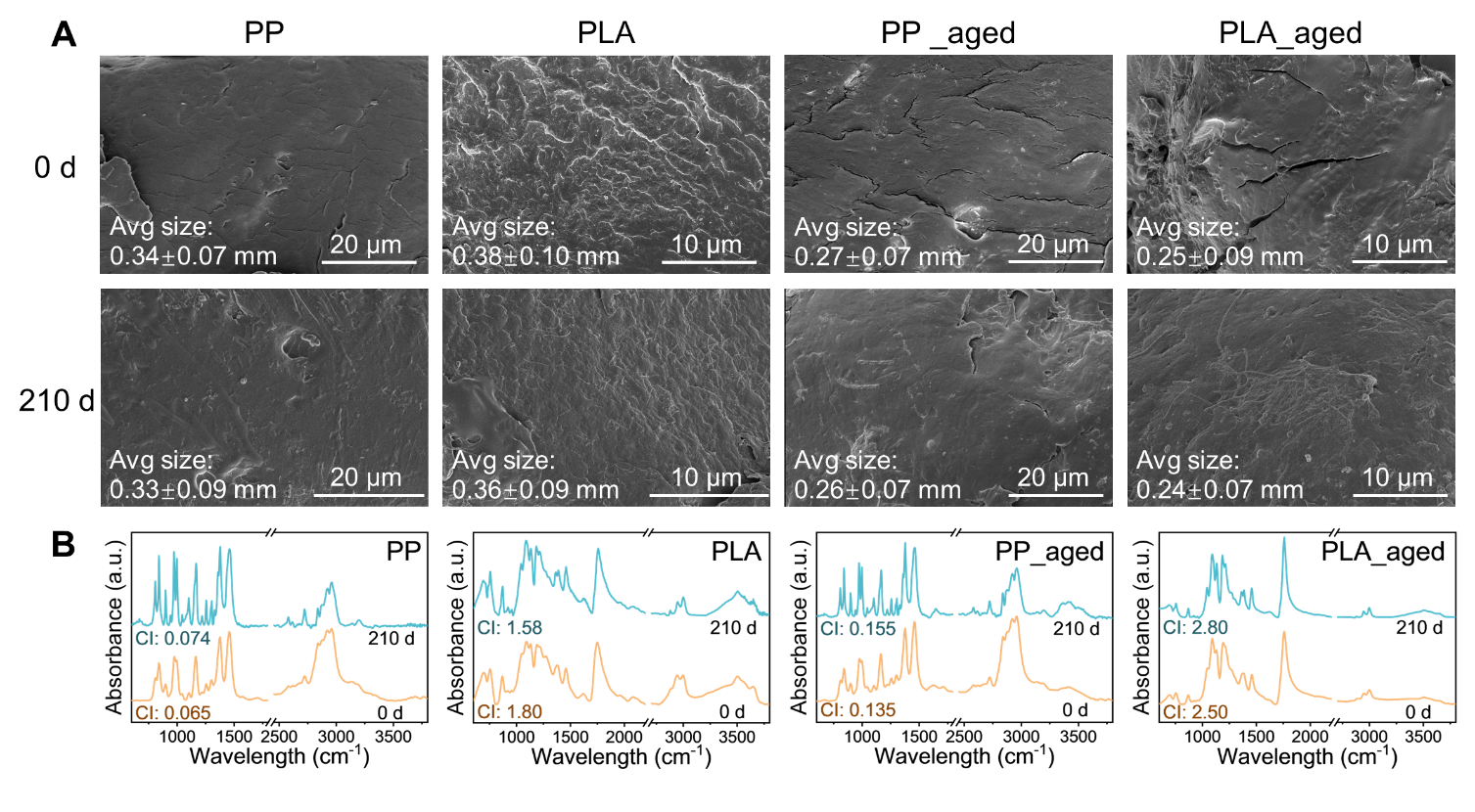
**

**Fig. S19. Physicochemical properties of original and 210-day incubated MPs in abiotic control groups.** (**A**) Surface morphology and average particle sizes, (**B**) FTIR spectra.


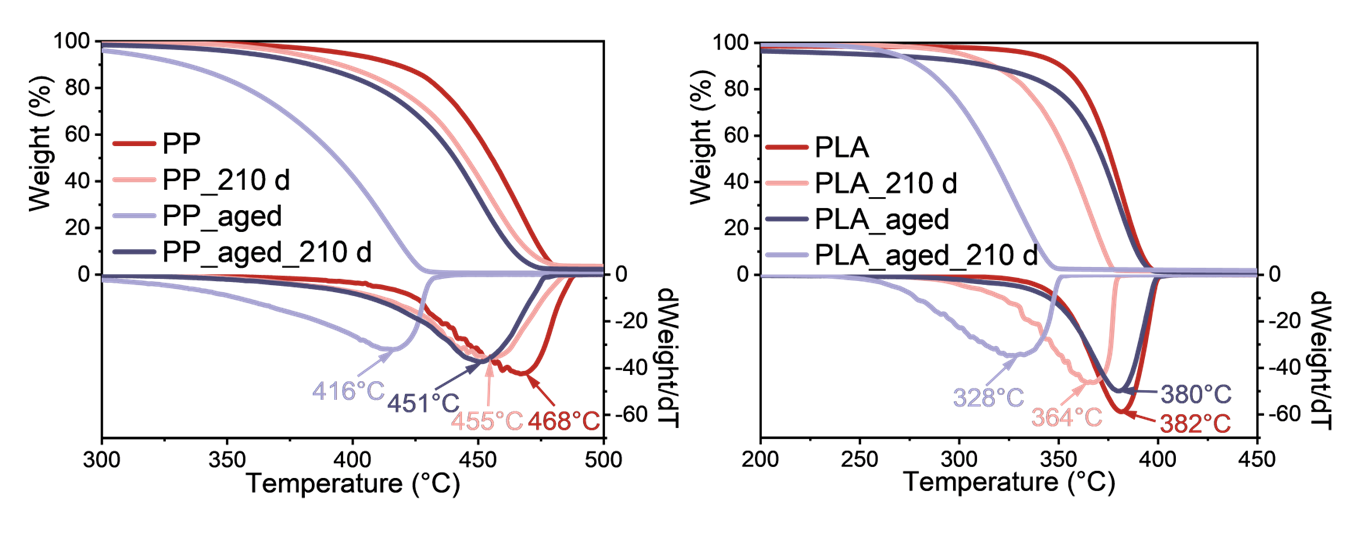


**Fig. S20. Thermal gravimetric analysis profiles of virgin and photoaged MPs during mesocosm incubation.**


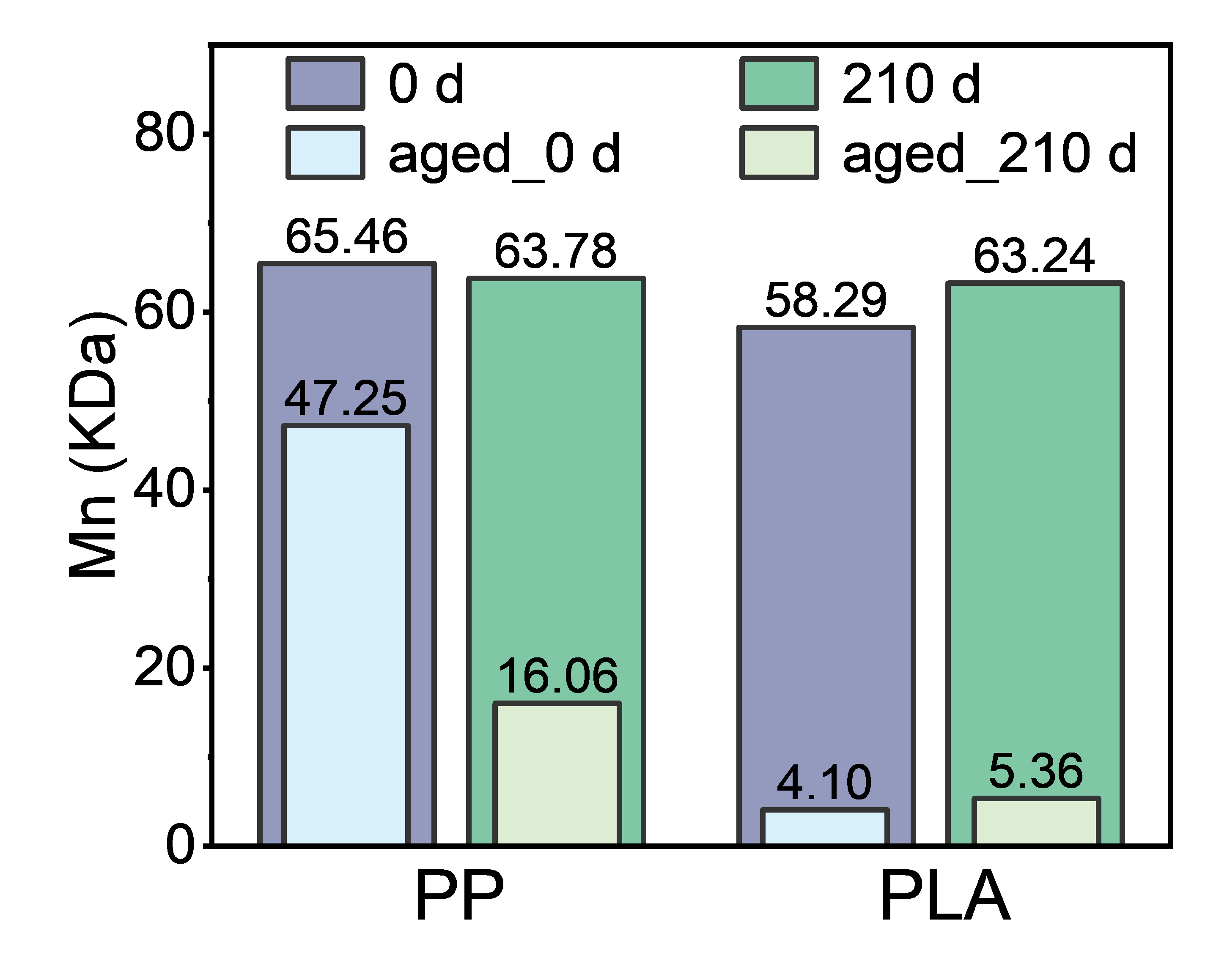


# Fig. S21. Mn of virgin and photoaged MPs during mesocosm incubation.

#
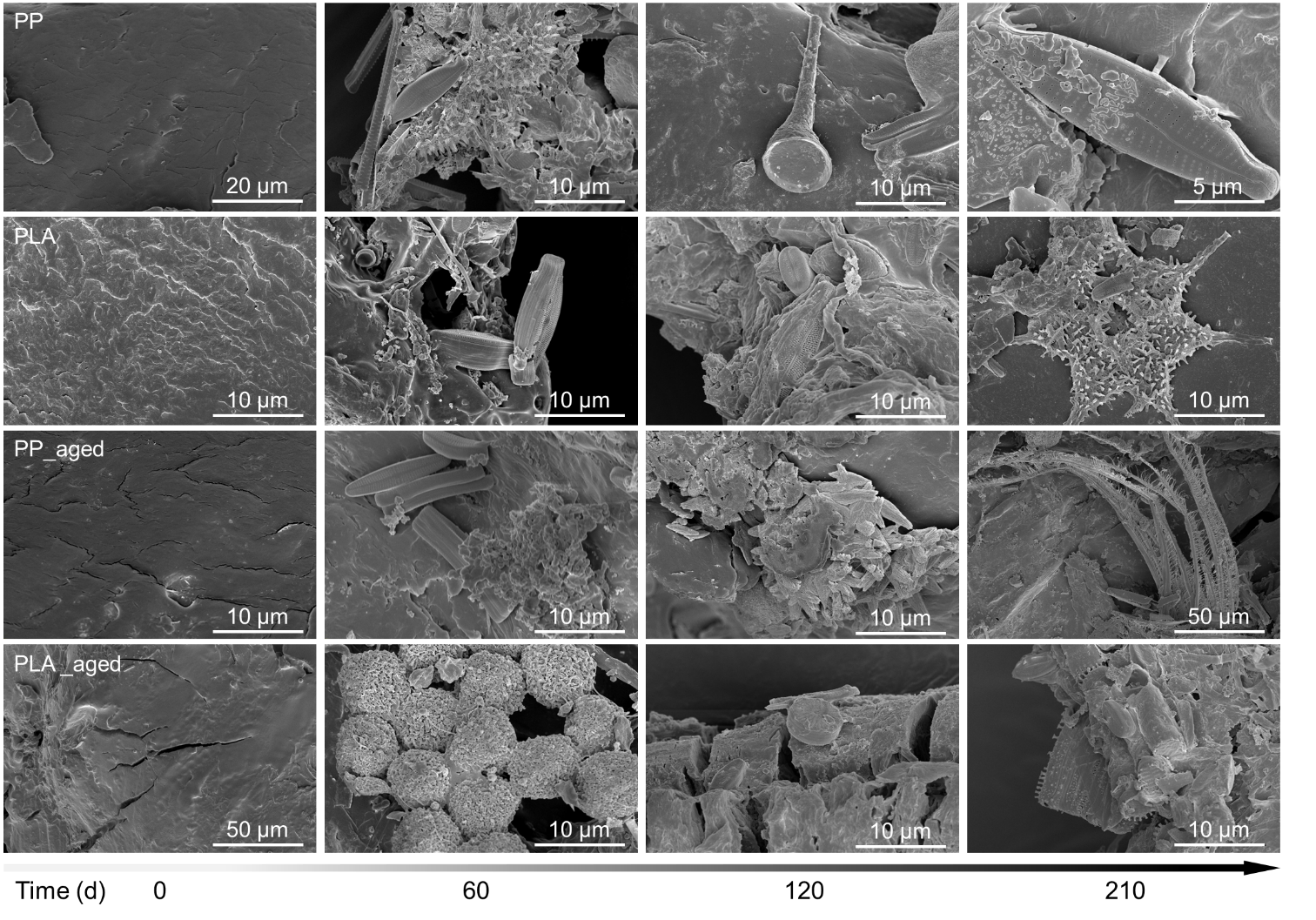


# Fig. S22. SEM images of attached microorganisms in biofilms of virgin and photoaged MPs during mesocosm incubation. Various microorganisms, including filamentous, bacillary bacteria, and ellipsoid diatoms were detected in MPs.


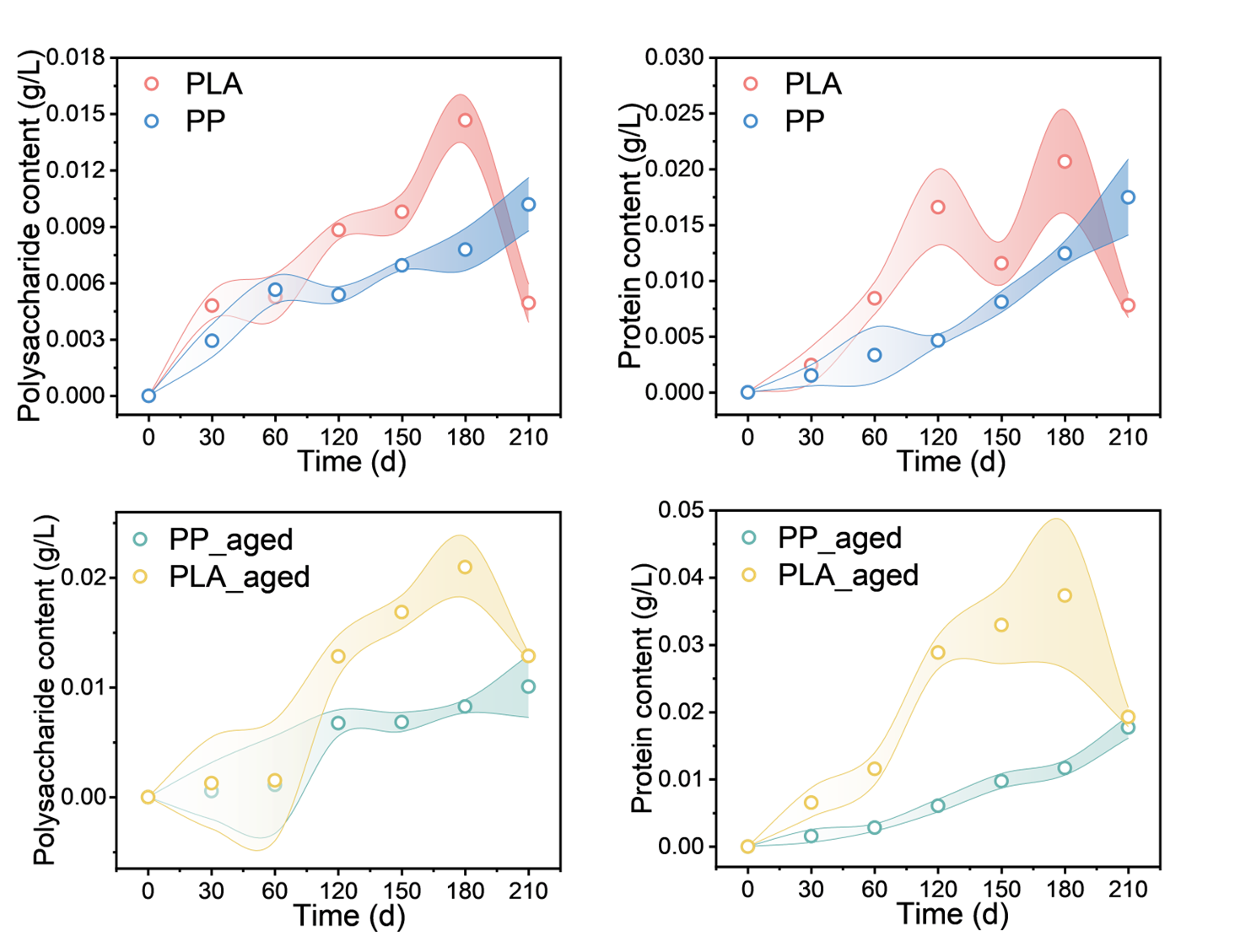


# Fig. S23. Polysaccharide and protein contents of virgin and photoaged MPs versus incubation time. Error bar represents the standard deviation from three replicate biofilm samples (n = 3).


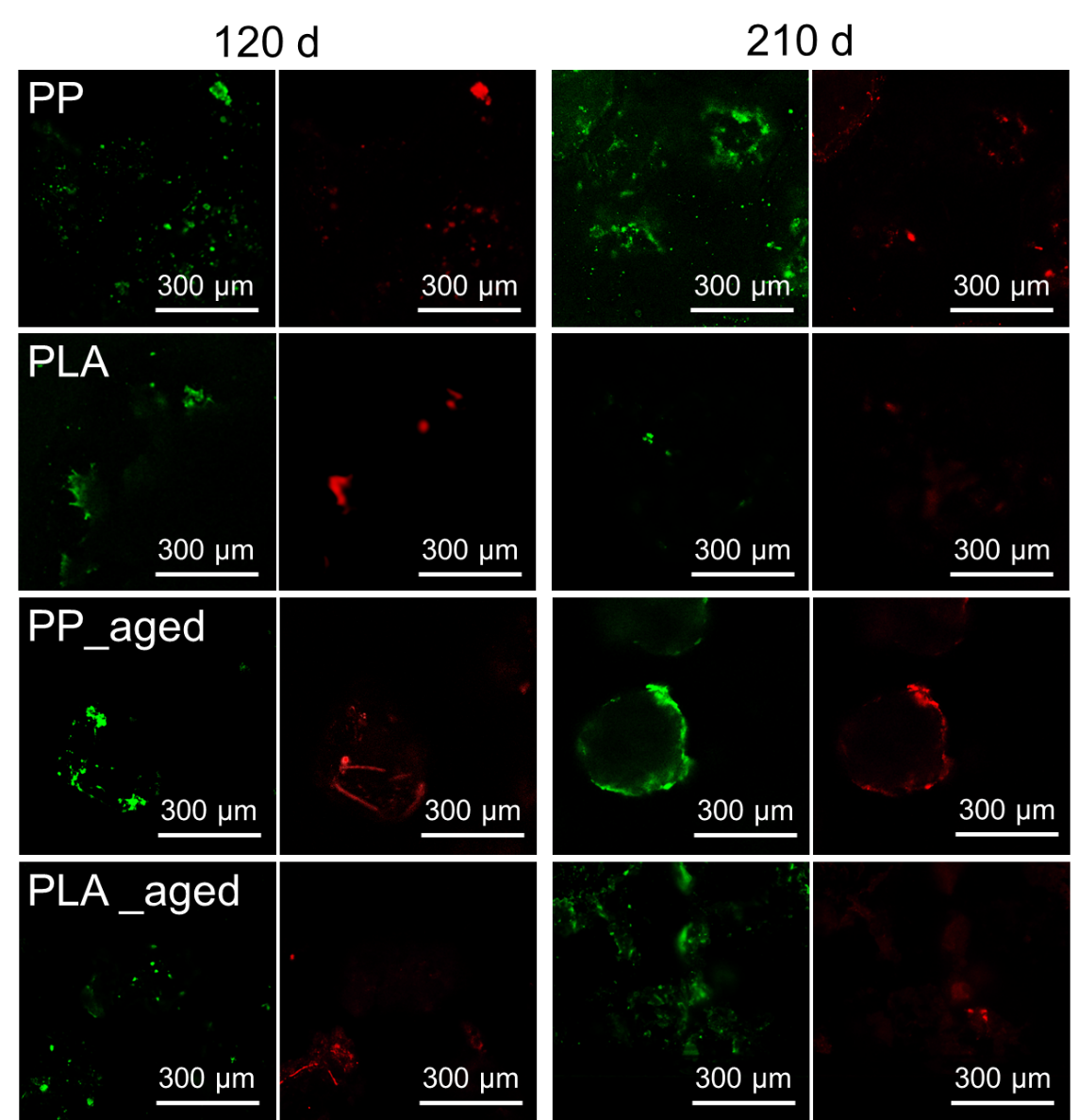


# Fig. S24. Confocal laser scanning microscope images of attached microorganisms on virgin and photoaged MPs during mesocosm incubation. Green and red signals denote the living and dead cells, respectively.


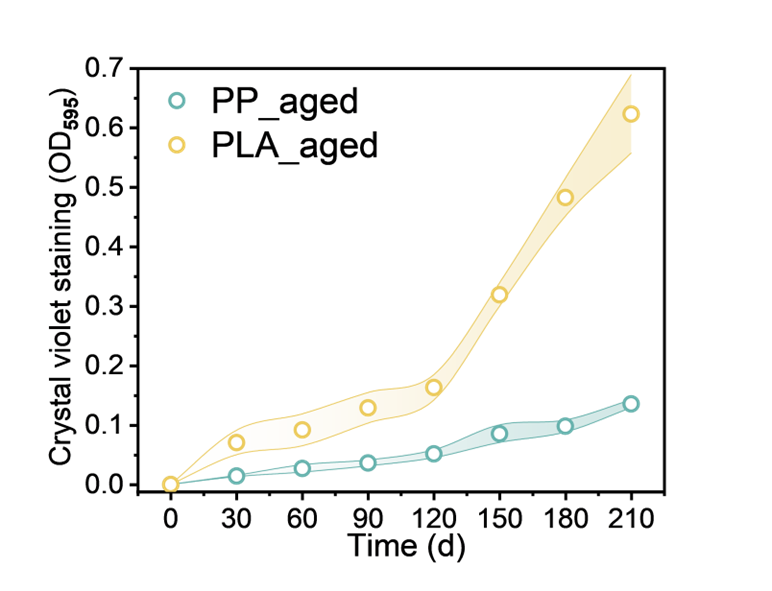


# Fig. S25. Biofilm biomass of photoaged MPs versus incubation time. Error bar represents the standard deviation from three replicate biofilm samples (n = 3).


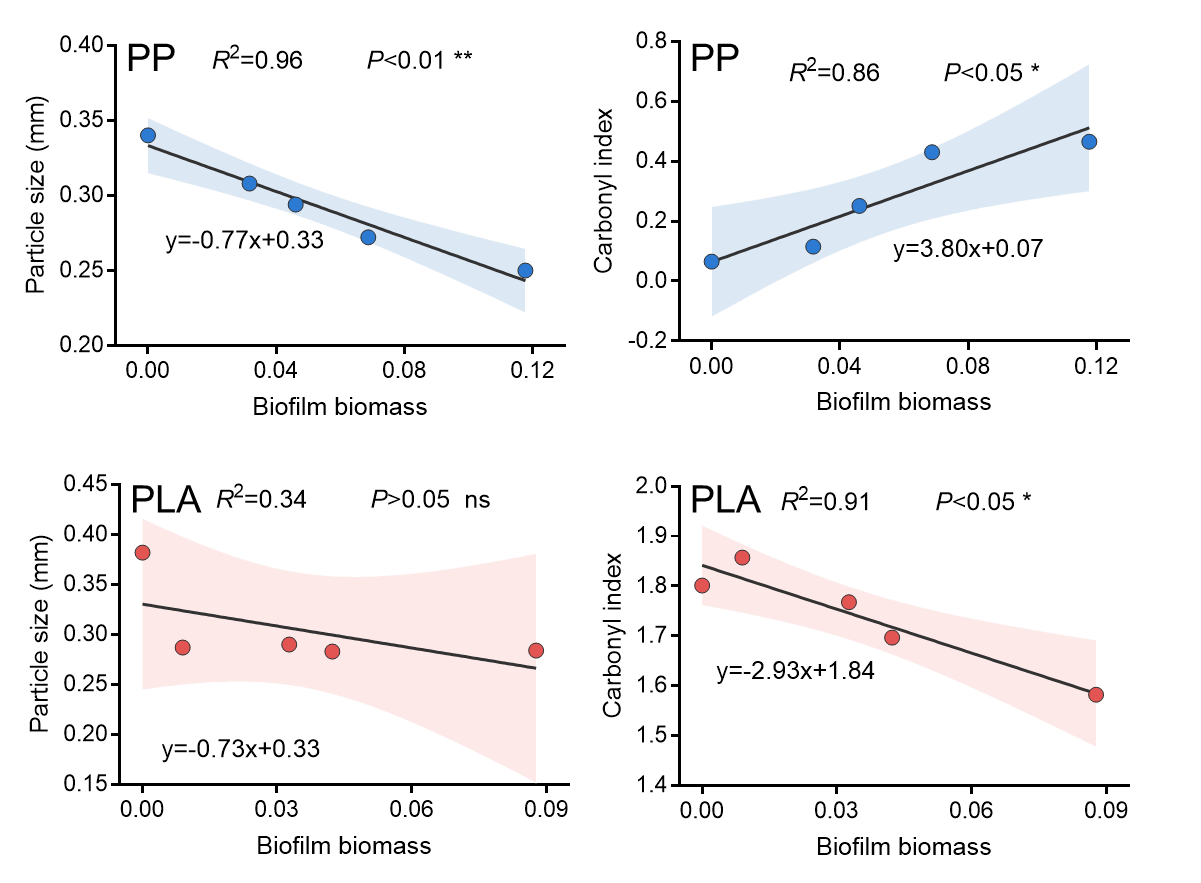


# Fig. S26. Correlation of carbonyl index and particle size with biofilm biomass of PP and PLA.

**
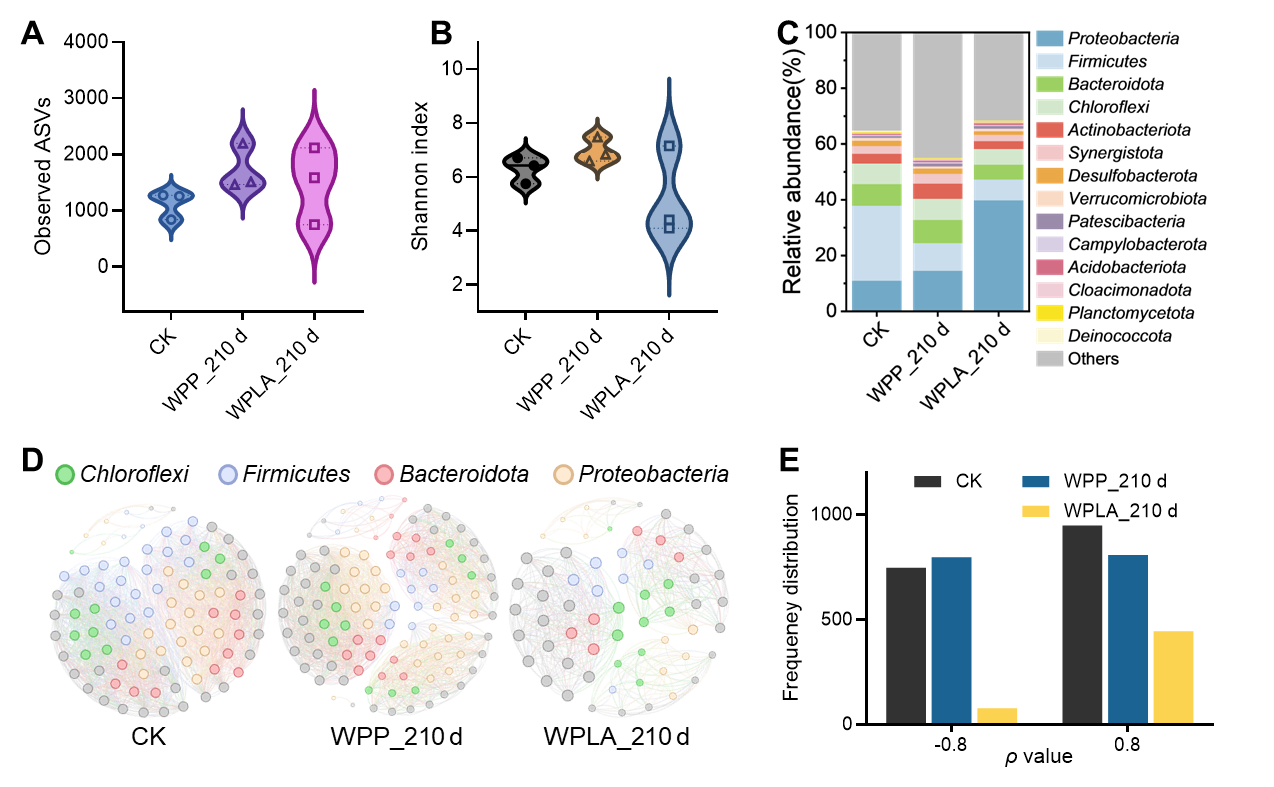
**

**Fig. S27. Taxonomic and functional profiling of freshwater microbiota.** (**A**) Observed ASVs and (**B**) Shannon index. (**C**) Microbial community compositions at the phylum level. (**D**) Co-occurrence networks and (**E**) distribution of positive and negative correlations based on ASVs. “CK”, “WPP_210 d”, and “WPLA_210 d” denote original water sample at 0 day, the water samples incubated with PP and PLA for 210 days, respectively.


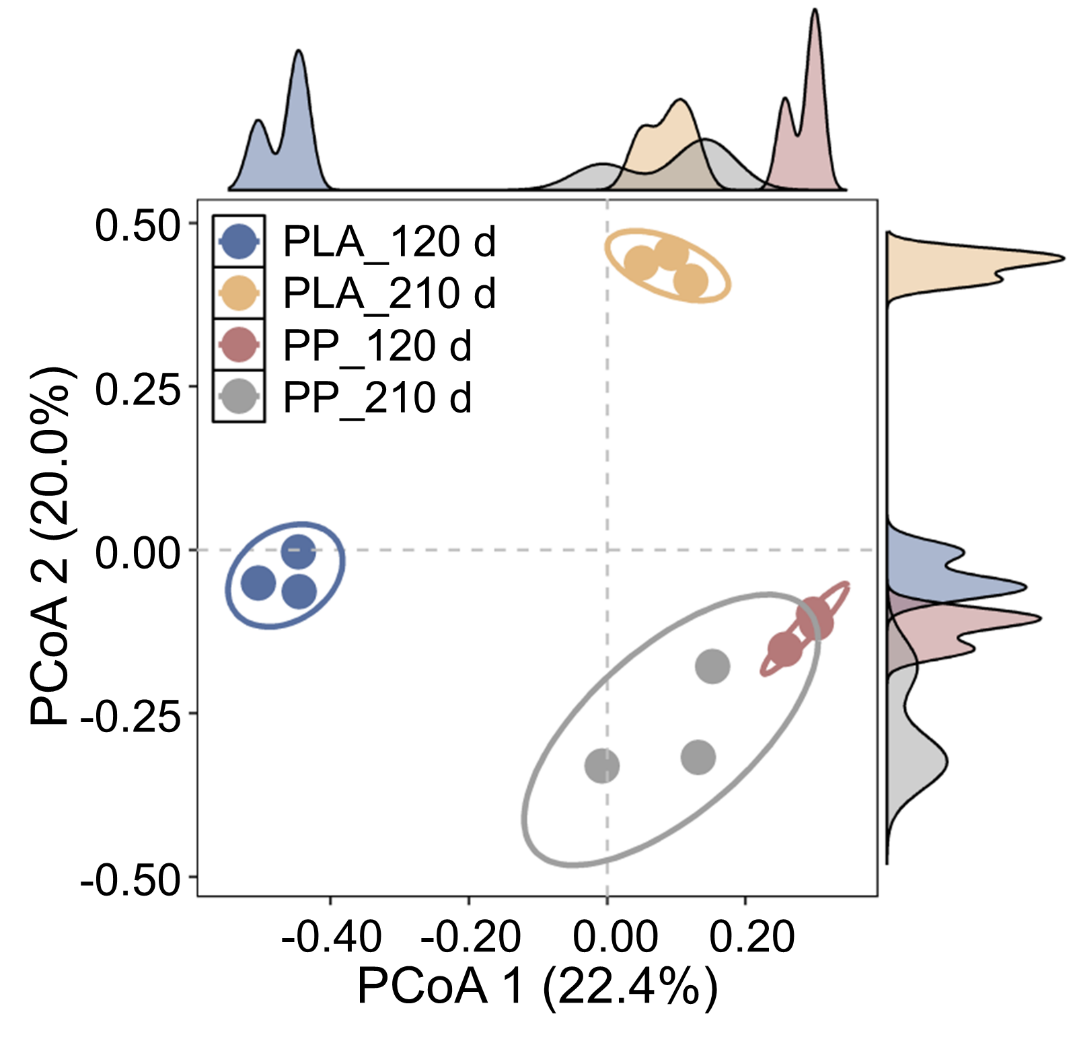


# **Fig. S28. Principal coordinate analysis of microbial communities in MP biofilms during mesocosm incubation.** 95.0% confidence ellipses were constructed for each sample type.


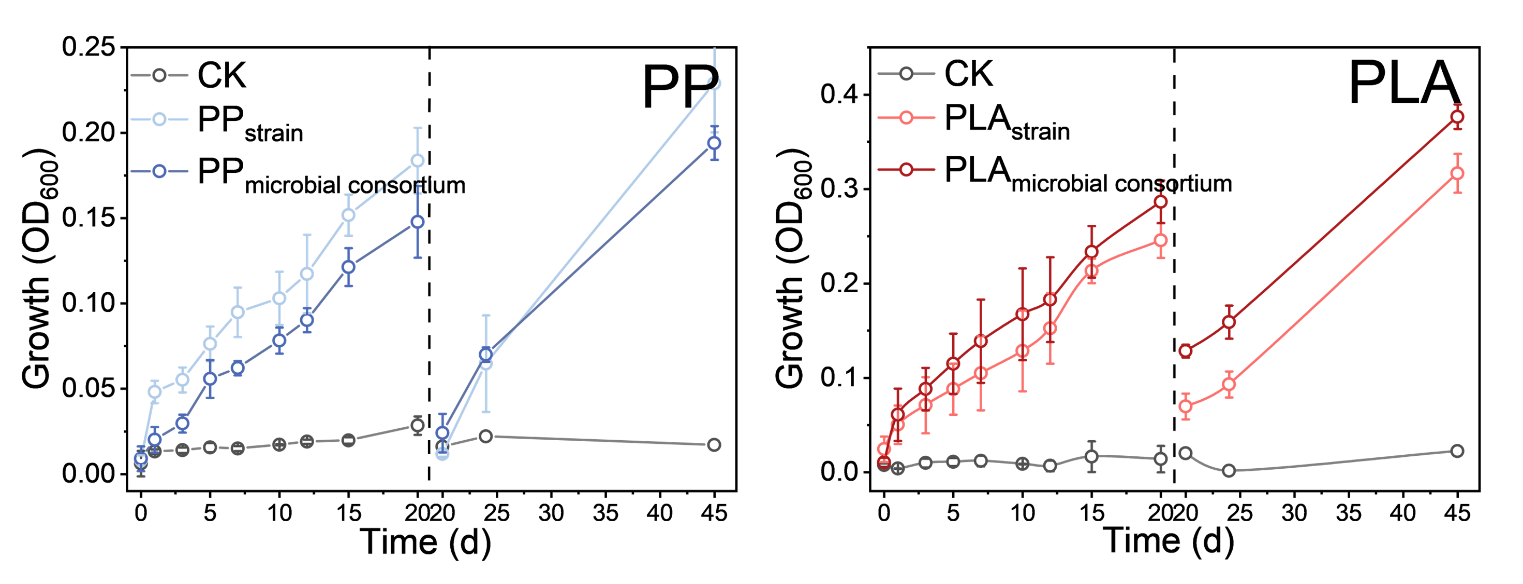


# **Fig. S29.** **Growth curves of** MP-degrading strains (PP_strain_ and PLA_strain_) and consortia (PP_consortium_ and PLA _consortium_) in **co-culture systems.** The inorganic salt medium was supplemented with PP or PLA served as the sole carbon source. CK represents a control group without bacterial strains. Error bars represent the standard deviation of three replicate medium (n = 3).


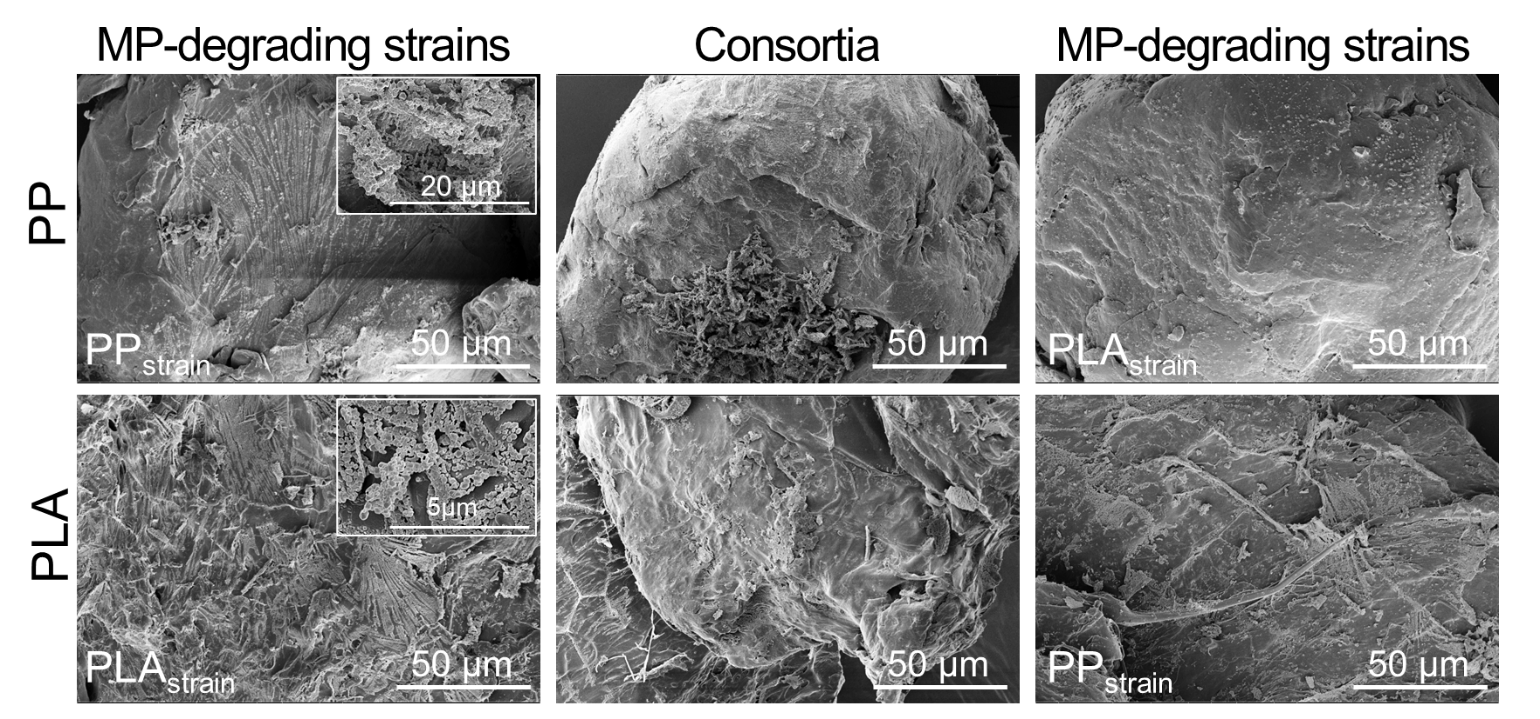


# **Fig. S30. SEM images of PP and PLA with attached bacteria after 45 days of co-culture with** MP-degrading strains or consortia**.**


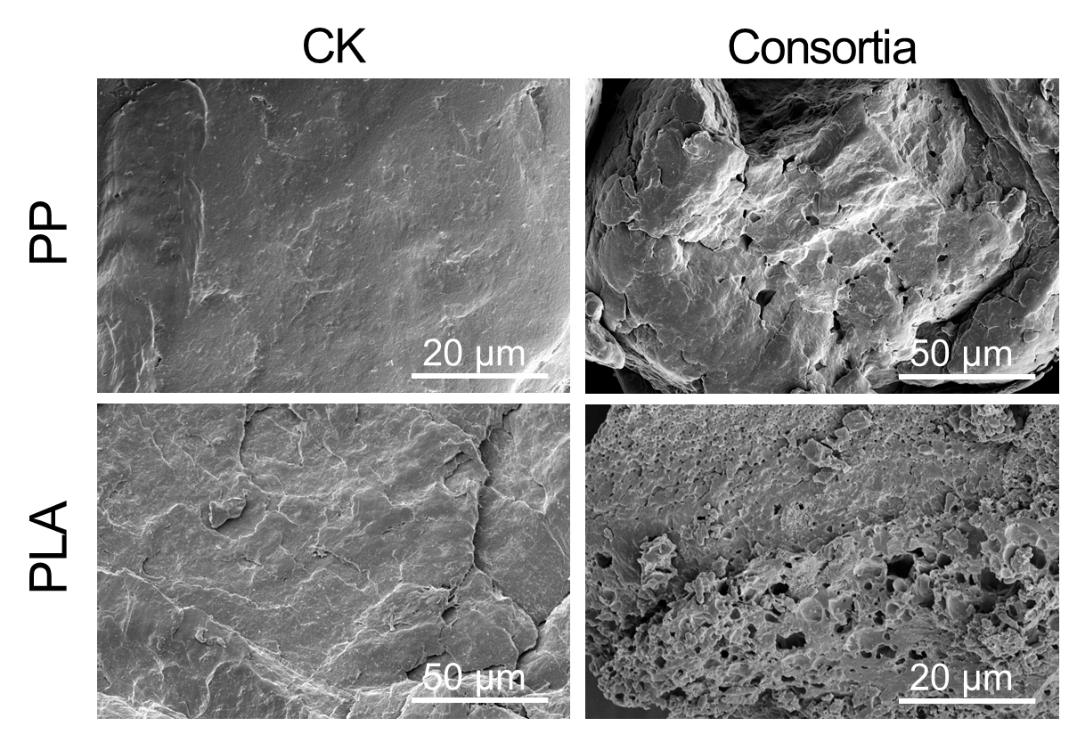


# **Fig. S31. SEM images of PP and PLA after co-culture with** MP-degrading **consortia.** CK represents virgin MPs before co-culture with MP-degrading consortia.


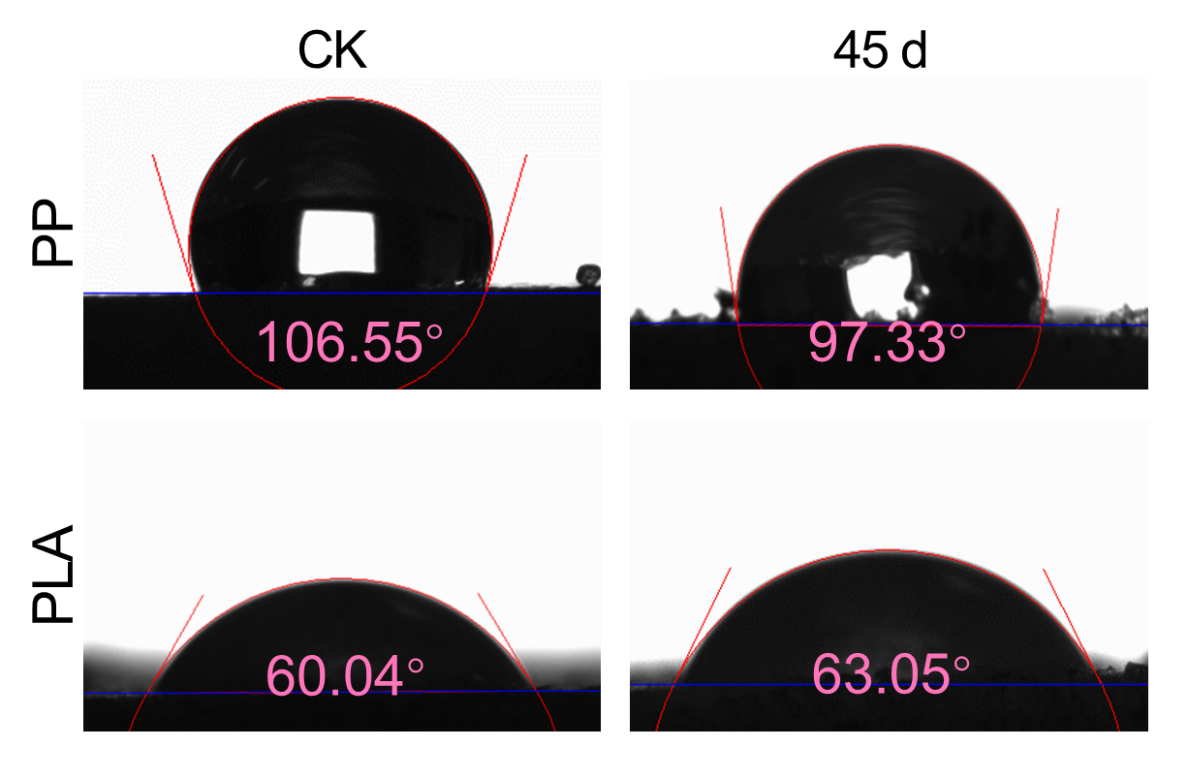


# Fig. S32. Water contact angle of PP and PLA after co-culture with MP-degrading strains. CK represents virgin MPs before co-culture with MP-degrading consortia.


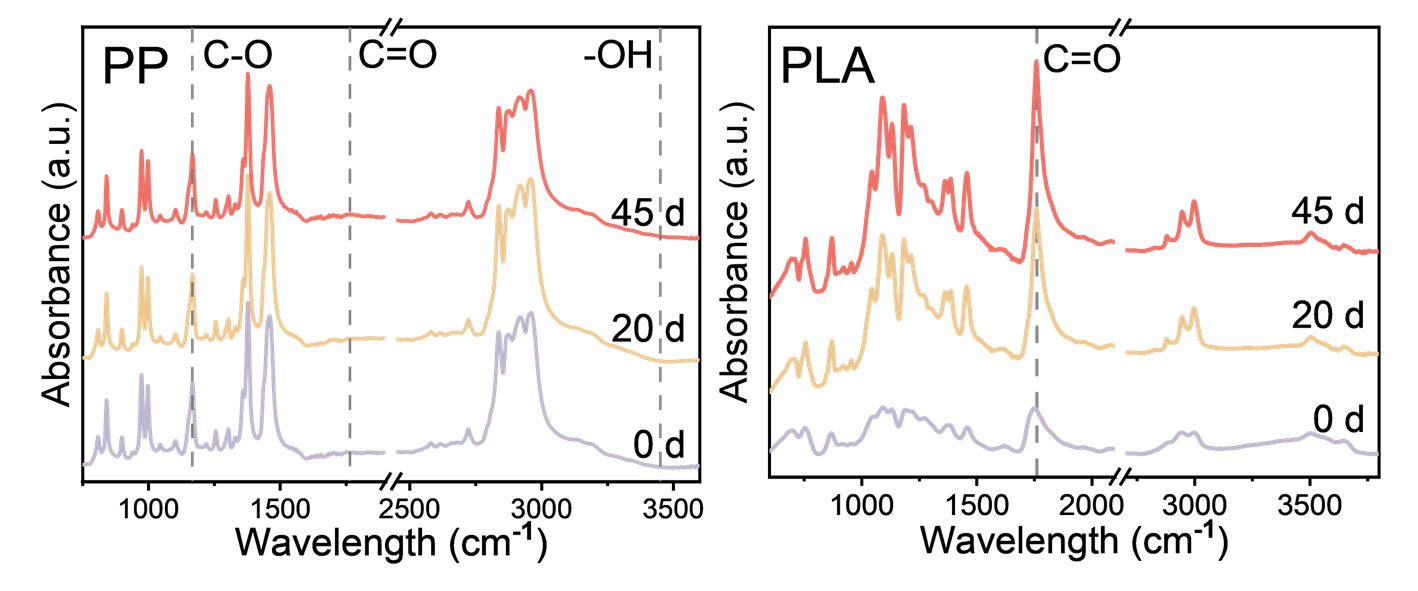


# Fig. S33. FTIR spectra of PP and PLA during **co-culture** with MP-degrading strains.


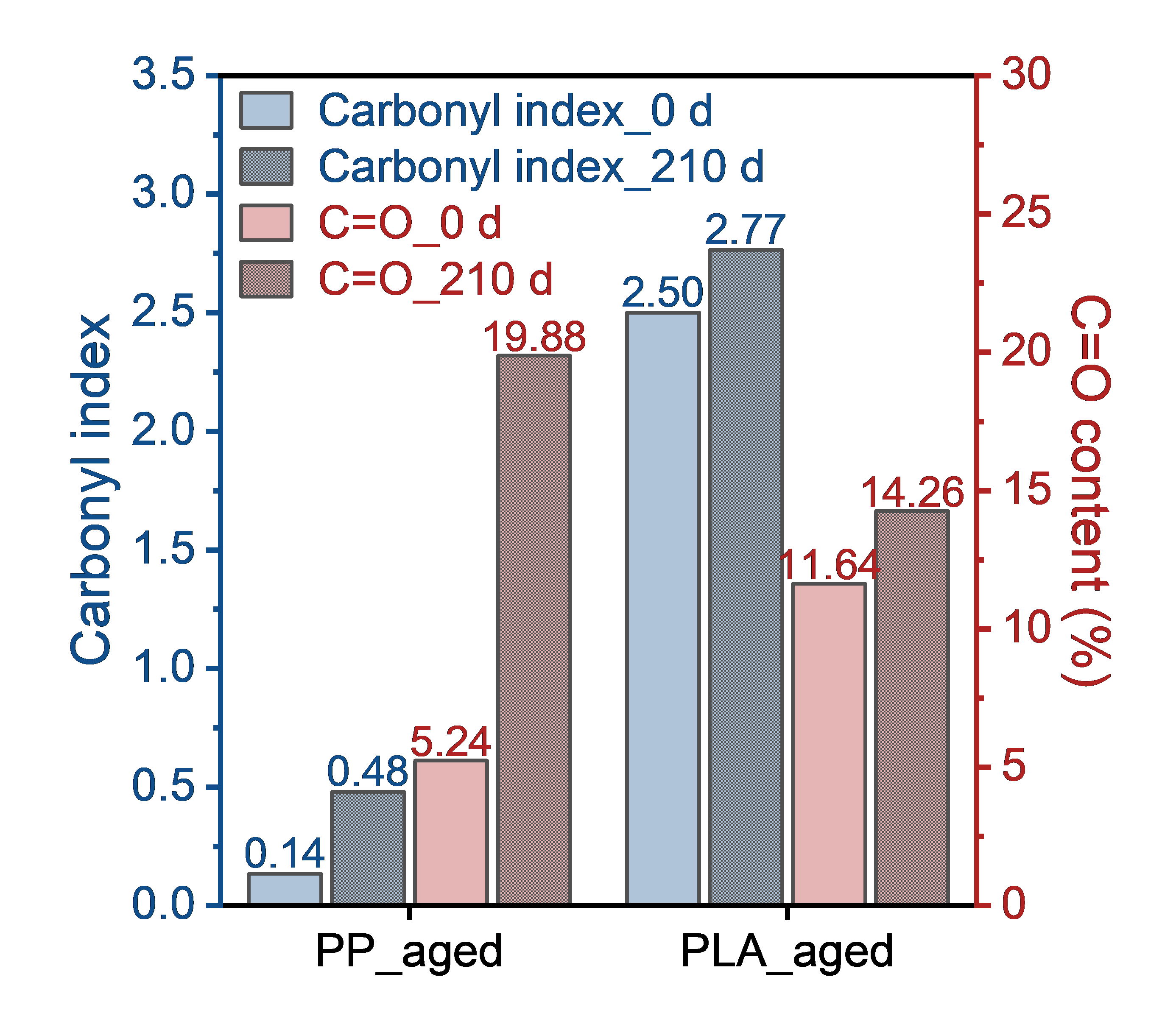


# Fig. S34. Carbonyl index and C=O content of photoaged PP and PLA during mesocosm incubation. The data were obtained from mesocosm incubation experiment.

# Table S1 The basic properties of PP, photoaged PP, PLA, and photoaged PLA.

| Subject | **virgin PP** | **PP_aged** | **virgin PLA** | **PLA_aged** |
| --- | --- | --- | --- | --- |
| Density (g/cm^3^) | 0.90–0.91 |  | 1.25–1.28 |  |
| Average sizes (mm) | 0.34 ± 0.07 | 0.27 ± 0.07 | 0.38 ± 0.10 | 0.25 ± 0.09 |
| Surface area (m^2^/g) | 0.22 | 0.63 | 0.16 | 0.50 |
| Carbon content (%) | 86.5 | 86.2 | 49.0 | 48.7 |
| Oxygen content (%) | 2.8 | 5.7 | 25.9 | 24.0 |
| Carbonyl index | 0.07 | 0.14 | 1.80 | 2.50 |
| T_m_ (°C) | 468 | 451 | 382 | 380 |
| Mn (Da) | 65456 | 47251 | 58290 | 4101 |
| Mw (Da) | 243225 | 154537 | 120423 | 30633 |
| Molecular structure | 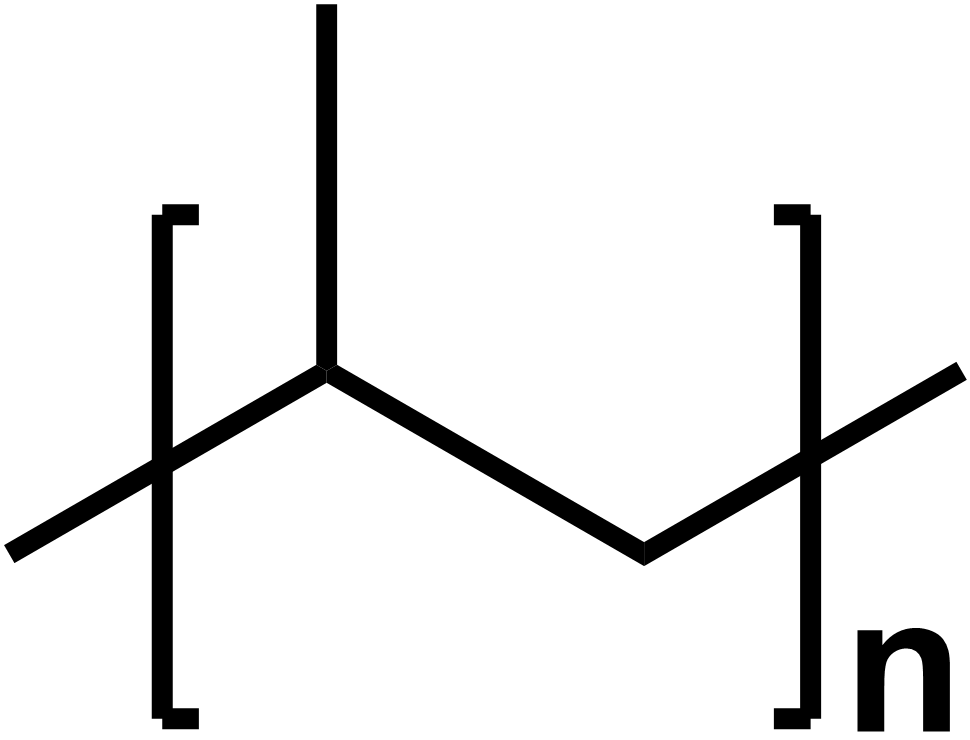 |  | 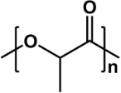 |  |

Note: The density was obtained from previous publications [46, 47]. Average sizes were determined by SEM in combination with Nano Measurer software. The specific surface areas were measured using a fully automatic physical adsorption analyzer. Carbon contents were obtained from an organic elemental analyzer. Oxygen contents were obtained from XPS results. Carbonyl index was calculated from the maximum intensity of the carbonyl group in FTIR spectra. Molecular structure information was provided by the manufacturer. T_m_ was measured by thermal gravimetric analysis. Mn and Mw were measured using GPC.

# Table S2 Inorganic salt composition.

| **Inorganic** | **Concentration (g/L)** |
| --- | --- |
| K_2_HPO_4_·3H_2_O | 0.917 |
| KH_2_PO_4_ | 0.700 |
| MgSO_4_·7H_2_O | 0.700 |
| KNO_3_ | 2.525 |
| NaCl | 0.005 |
| FeSO_4_·7H_2_O | 0.002 |
| ZnSO_4_·7H_2_O | 0.002 |
| MnSO_4_·H_2_O | 0.001 |

Note: The XPS results showed that the carbon content of PP and PLA were 80.0% and 60.0%, respectively. Based on the proportion of carbon source in the medium of 0.5% the addition was calculated to be 6.3 g/L for PP and 8.3 g/L for PLA [48].

# Table S3 The particle sizes of various types of MPs during 210 days of mesocosm incubation.

|  | **Time (d)** | **Avg (mm)** | **Max (mm)** | **Min (mm)** | **Med (mm)** |
| --- | --- | --- | --- | --- | --- |
| PP | 0 | 0.34 | 0.50 | 0.09 | 0.34 |
|  | 60 | 0.31 | 0.56 | 0.04 | 0.32 |
|  | 120 | 0.29 | 0.56 | 0.06 | 0.29 |
|  | 180 | 0.27 | 0.47 | 0.03 | 0.27 |
|  | 210 | 0.25 | 0.51 | 0.05 | 0.26 |
| PLA | 0 | 0.38 | 0.53 | 0.16 | 0.39 |
|  | 60 | 0.29 | 0.53 | 0.04 | 0.29 |
|  | 120 | 0.29 | 0.61 | 0.06 | 0.27 |
|  | 180 | 0.28 | 0.57 | 0.04 | 0.26 |
|  | 210 | 0.28 | 0.55 | 0.04 | 0.26 |
| PP_aged | 0 | 0.27 | 0.44 | 0.09 | 0.27 |
|  | 60 | 0.25 | 0.43 | 0.06 | 0.25 |
|  | 120 | 0.24 | 0.46 | 0.07 | 0.24 |
|  | 180 | 0.22 | 0.40 | 0.03 | 0.22 |
|  | 210 | 0.19 | 0.40 | 0.05 | 0.19 |
| PLA_aged | 0 | 0.30 | 0.66 | 0.07 | 0.31 |
|  | 60 | 0.25 | 0.66 | 0.05 | 0.25 |
|  | 120 | 0.24 | 0.59 | 0.06 | 0.24 |
|  | 180 | 0.23 | 0.58 | 0.03 | 0.19 |
|  | 210 | 0.23 | 0.59 | 0.02 | 0.18 |

Note: Avg, Max, Min, and Med denote average, maximum, minimum, and median particle sizes of MPs and photoaged MPs, respectively.

# Table S4 Peak fitting analysis on the relative percentages of chemical bonds of virgin and photoaged MPs during mesocosm incubation in high-resolution oxygen 1s XPS spectra.

| **XPS (Atomic%)** | | **C=O** | **C**‒**O** | ‒**OH** | **O**‒**C=O** |
| --- | --- | --- | --- | --- | --- |
| PP | 0 d | - | - | - | - |
|  | 120 d | 17.8% | 56.1% | 24.6% | 1.5% |
|  | 210 d | 16.5% | 45.9% | 33.2% | 4.5% |
| PLA | 0 d | 24.1% | 32.8% | 0.1% | 43.1% |
|  | 120 d | 47.6% | 19.0% | 9.6% | 23.8% |
|  | 210 d | 12.1% | 32.7% | 21.5% | 33.8% |
| PP_aged | 0 d | 5.2% | 57.9% | 36.9% | 0.0% |
|  | 120 d | 15.5% | 60.7% | 18.2% | 5.6% |
|  | 210 d | 19.9% | 48.8% | 27.1% | 4.2% |
| PLA_aged | 0 d | 11.6% | 33.1% | 47.8% | 7.5% |
|  | 120 d | 6.8% | 46.0% | 35.8% | 11.3% |
|  | 210 d | 14.3% | 38.3% | 38.8% | 8.7% |

# Table S5 Oxidation property and carbon content of MPs in original and 210 days of incubation in abiotic control groups.

|  | **Time (d)** | | **Carbonyl index** | **O/C ratio** | **Carbon content (%)** |
| --- | --- | --- | --- | --- | --- |
| PP | | 0 | 0.065 | 0.032 | 86.5 |
|  |  | 210 | 0.074 | 0.035 | 84.9 |
| PLA | | 0 | 1.80 | 0.53 | 49.0 |
|  |  | 210 | 1.73 | 0.51 | 48.2 |
| PP_aged | | 0 | 0.135 | 0.066 | 86.5 |
|  |  | 210 | 0.155 | 0.050 | 84.4 |
| PLA_aged | | 0 | 2.50 | 0.49 | 48.7 |
|  |  | 210 | 2.57 | 0.59 | 49.0 |

Note: Carbonyl index was calculated from the maximum intensity of the carbonyl group relative to the methylene group in FTIR spectra. O/C ratios were obtained from XPS results. Carbon contents were obtained from an organic elemental analyzer.

# Table S6 The molecular weight of various types of MPs during 210 days of mesocosm incubation.

|  | **Time (d)** | **Mp (Da)** | **Mn (Da)** | **Mw (Da)** | **Mz (Da)** | **Mz + 1 (Da)** | **Mv (Da)** | **PDI** |
| --- | --- | --- | --- | --- | --- | --- | --- | --- |
| PP | 0 | 162624 | 65456 | 243225 | 607726 | 1057671 | 209033 | 3.72 |
|  | 210 | 147684 | 63776 | 234885 | 571049 | 964332 | 202455 | 3.68 |
| PLA | 0 | 120986 | 58290 | 120423 | 193292 | 265160 | 110623 | 2.07 |
|  | 210 | 98899 | 63242 | 103954 | 149708 | 191217 | 97523 | 1.64 |
| PP_aged | 0 | 102894 | 47251 | 154537 | 372168 | 624135 | 133671 | 3.27 |
|  | 210 | 33969 | 16064 | 58128 | 161720 | 316036 | 49547 | 3.62 |
| PLA_aged | 0 | 8049 | 4101 | 30633 | 109113 | 170011 | 23242 | 7.47 |
|  | 210 | 12883 | 5361 | 30776 | 80441 | 124156 | 25278 | 5.74 |

Note: Mp, peak molecular weight; Mz, z-average molecular weight; Mz + 1, (z + 1)‑average molecular weight; Mv, viscosity‑average molecular weight; PDI, polydispersity index.

# Table S7 MP-degrading strains with ASVs data aligned in PlasticDB.

|  | **ASV** | **Genus** | **Species** | **Reference** |
| --- | --- | --- | --- | --- |
| PP_120 d | ASV807 | *Achromobacter* | *Achromobacter_arsenitoxydans* | [49] |
|  | ASV13765 | *Acidovorax* | *Acidovorax_facilis* | [50] |
|  | ASV13543 | *Acidovorax* | *Acidovorax_valerianellae* | [51] |
|  | ASV20987 | *Acidovorax* | *Acidovorax_wautersii* | [50] |
|  | ASV11574 | *Acidovorax* | *beta_proteobacterium_*BIWA18 | [50] |
|  | ASV11640 | *Acidovorax* | *beta_proteobacterium_*BIWA22 | [51] |
|  | ASV21581 | *Bacteroides* | *Bacteroides_massiliensis_*B84634_Timone_  84634_DSM_17679_JCM_13223 | [52] |
|  | ASV21573 | *Bacteroides* | *Bacteroides_vulgatus* | [52] |
|  | ASV20248 | *Bacteroides* | *Phocaeicola_vulgatus* | [52] |
|  | ASV2992 | *Bdellovibrio* | *Bdellovibrionales_bacterium_*Ga0074137 | [53] |
|  | ASV21540 | *Bdellovibrio* | *Bdellovibrionales_bacterium_*  RIFCSPHIGHO2_01_FULL_40_29 | [53] |
|  | ASV607 | *Bdellovibrio* | *unclassified_Bdellovibrio* | [53] |
|  | ASV6238 | *Brevundimonas* | *Brevundimonas_aveniformis* | [54] |
|  | ASV3127 | *Brevundimonas* | *Brevundimonas_nasdae* | [55] |
|  | ASV6289 | *Brevundimonas* | *Brevundimonas_subvibrioides* | [55] |
|  | ASV20948 | *Chitinimonas* | *Chitinimonas_taiwanensis* | [56] |
|  | ASV11015 | *Chitinimonas* | *Chitinimonas_viridis* | [56] |
|  | ASV11592 | *Chitinimonas* | *unclassified_Chitinimonas* | [56] |
|  | ASV21585 | *Delftia* | *Delftia_tsuruhatensis* | [57] |
|  | ASV20142 | *Desulfovibrio* | *Desulfovibrio_putealis* | [52] |
|  | ASV21584 | *Flavobacterium* | *Flavobacterium_anseonense* | [58] |
|  | ASV21099 | *Flavobacterium* | *Flavobacterium_collinsii* | [58] |
|  | ASV20977 | *Flavobacterium* | *Flavobacterium_nackdongense* | [58] |
|  | ASV21566 | *Flavobacterium* | *Flavobacterium_tructae* | [58] |
|  | ASV20945 | *Halomonas* | *Candidatus_Halomonas_phosphatis* | [59] |
|  | ASV2991 | *Ideonella* | *Aquincola_tertiaricarbonis* | [60] |
|  | ASV1683 | *Ideonella* | *beta_proteobacterium_*A0640 | [60] |
|  | ASV12368 | *Ideonella* | *Hyalella_azteca* | [60] |
|  | ASV20181 | *Ideonella* | *Ideonella_dechloratans* | [61] |
|  | ASV20324 | *Ideonella* | *Ideonella_*sp. | [60] |
|  | ASV1338 | *Ideonella* | *Rubrivivax_gelatinosus* | [60] |
|  | ASV21538 | *Leptothrix* | *beta_proteobacterium_*AAP121 | [62] |
|  | ASV1501 | *Leptothrix* | *beta_proteobacterium_*AAP51 | [63] |
|  | ASV1789 | *Leptothrix* | *beta_proteobacterium_*LF4_45 | [63] |
|  | ASV1687 | *Leptothrix* | *Burkholderiales_bacterium* | [63] |
|  | ASV20919 | *Leptothrix* | *Leptothrix_ginsengisoli* | [62] |
|  | ASV21569 | *Leptothrix* | *Leptothrix_mobilis* | [63] |
|  | ASV21027 | *Mitsuaria* | *Mitsuaria_chitosanitabida* | [64] |
|  | ASV17178 | *Pseudomonas* | *gamma_proteobacterium_*BIWA53 | [65] |
|  | ASV13560 | *Pseudomonas* | *Pseudomonas_alcaligenes* | [65] |
|  | ASV20925 | *Pseudomonas* | *Pseudomonas_anguilliseptica* | [65] |
|  | ASV20242 | *Pseudomonas* | *Pseudomonas_chengduensis* | [66] |
|  | ASV1900 | *Pseudomonas* | *Pseudomonas_fluorescens* | [65] |
|  | ASV1648 | *Pseudomonas* | *Pseudomonas_mandelii* | [66] |
|  | ASV5573 | *Pseudomonas* | *Pseudomonas_mendocina* | [65] |
|  | ASV21002 | *Pseudomonas* | *Pseudomonas_oryzihabitans* | [66] |
|  | ASV21029 | *Rheinheimera* | *gamma_proteobacterium_F8* | [67] |
|  | ASV4649 | *Rheinheimera* | *Pararheinheimera_arenilitoris* | [67] |
|  | ASV21597 | *Rheinheimera* | *Pararheinheimera_chironomi* | [67] |
|  | ASV19361 | *Rheinheimera* | *Pararheinheimera_tilapiae* | [67] |
|  | ASV2076 | *Rheinheimera* | *Rheinheimera_tangshanensis* | [67] |
|  | ASV17609 | *Rhodoferax* | *beta_proteobacterium_*HIBAF004 | [64] |
|  | ASV21567 | *Rhodoferax* | *beta_proteobacterium_*HIBAF013 | [64] |
|  | ASV1666 | *Rhodoferax* | *Curvibacter_putative_symbiont_of_*  *Hydra_magnipapillata* | [64] |
|  | ASV8672 | *Rhodoferax* | *Rhodoferax_ferrireducens* | [64] |
|  | ASV21554 | *Rhodoferax* | *Rhodoferax_saidenbachensis* | [64] |
|  | ASV1737 | *Rhodoferax* | *Rhodoferax_sediminis* | [64] |
|  | ASV17193 | *Rhodoferax* | *Rhodoferax_*sp. | [64] |
|  | ASV13539 | *Roseateles* | *Roseateles_depolymerans* | [68] |
|  | ASV19306 | *Shewanella* | *Shewanella_baltica* | [69] |
|  | ASV4074 | *Shewanella* | *Shewanella_putrefaciens* | [70] |
|  | ASV1300 | *Sphingomonas* | *Sphingomonas_fonticola* | [71] |
|  | ASV11235 | *Streptococcus* | *Streptococcus_pasteurianus* | [72] |
|  | ASV5645 | *Undibacterium* | *beta_proteobacterium_*Wuba70 | [56] |
|  | ASV2225 | *Undibacterium* | *Undibacterium_pigrum* | [56] |
|  | ASV20947 | *Undibacterium* | *Undibacterium_seohonense* | [56] |
| PP_210 d | ASV807 | *Achromobacter* | *Achromobacter_arsenitoxydans* | [49] |
|  | ASV13765 | *Acidovorax* | *Acidovorax_facilis* | [50] |
|  | ASV13543 | *Acidovorax* | *Acidovorax_valerianellae* | [51] |
|  | ASV13531 | *Acidovorax* | *Acidovorax_wautersii* | [50] |
|  | ASV2806 | *Acidovorax* | *beta_proteobacterium_*BIWA18 | [51] |
|  | ASV13532 | *Acidovorax* | *beta_proteobacterium_*BIWA22 | [50] |
|  | ASV461 | *Acinetobacter* | *Acinetobacter_lwoffii* | [73] |
|  | ASV13564 | *Azospirillum* | *unclassified_Azospirillum* | [74] |
|  | ASV9825 | *Bacteroides* | *Bacteroides_fluxus* | [52] |
|  | ASV10066 | *Bacteroides* | *Bacteroides_fragilis* | [52] |
|  | ASV14601 | *Bacteroides* | *Bacteroides_*sp.*_Marseille_*P3166 | [52] |
|  | ASV11708 | *Bacteroides* | *Bacteroides_vulgatus* | [52] |
|  | ASV128 | *Bacteroides* | *Phocaeicola_barnesiae* | [52] |
|  | ASV14300 | *Bacteroides* | *uncultured_Bacteroidales_bacterium* | [52] |
|  | ASV2992 | *Bdellovibrio* | *Bdellovibrionales_bacterium_*Ga0074137 | [53] |
|  | ASV607 | *Bdellovibrio* | *unclassified_Bdellovibrio* | [53] |
|  | ASV6238 | *Brevundimonas* | *Brevundimonas_aveniformis* | [55] |
|  | ASV3129 | *Brevundimonas* | *Brevundimonas_bacteroides* | [54] |
|  | ASV3451 | *Brevundimonas* | *Brevundimonas_balnearis* | [55] |
|  | ASV2840 | *Brevundimonas* | *Brevundimonas_denitrificans* | [54] |
|  | ASV11677 | *Brevundimonas* | *Brevundimonas_fluminis* | [55] |
|  | ASV7601 | *Brevundimonas* | *Brevundimonas_humi* | [54] |
|  | ASV6241 | *Brevundimonas* | *Brevundimonas_intermedia* | [55] |
|  | ASV13568 | *Brevundimonas* | *Brevundimonas_kwangchunensis* | [54] |
|  | ASV11743 | *Brevundimonas* | *Brevundimonas_nasdae* | [55] |
|  | ASV11555 | *Brevundimonas* | *Brevundimonas_subvibrioides* | [54] |
|  | ASV4007 | *Brevundimonas* | *Brevundimonas_variabilis* | [54] |
|  | ASV11592 | *Chitinimonas* | *unclassified_Chitinimonas* | [56] |
|  | ASV11911 | *Desulfovibrio* | *Desulfovibrio_bacterium_New_Zealand_D* | [52] |
|  | ASV11613 | *Desulfovibrio* | *Desulfovibrio_putealis* | [52] |
|  | ASV1700 | *Flavobacterium* | *Flavobacterium_anseonense* | [58] |
|  | ASV5558 | *Flavobacterium* | *Flavobacterium_nackdongense* | [58] |
|  | ASV2800 | *Flavobacterium* | *Flavobacterium_tructae* | [58] |
|  | ASV7749 | *Flavobacterium* | *uncultured_Bacteroidetes_bacterium* | [58] |
|  | ASV11570 | *Ideonella* | *Aquincola_tertiaricarbonis* | [61] |
|  | ASV1683 | *Ideonella* | *beta_proteobacterium_*A0640 | [60] |
|  | ASV1896 | *Ideonella* | *Hyalella_azteca* | [60] |
|  | ASV11531 | *Ideonella* | *Ideonella_dechloratans* | [60] |
|  | ASV1338 | *Ideonella* | *Rubrivivax_gelatinosus* | [60] |
|  | ASV1639 | *Leptothrix* | *beta_proteobacterium_*AAP121 | [63] |
|  | ASV13635 | *Leptothrix* | *beta_proteobacterium_*AAP51 | [62] |
|  | ASV1949 | *Leptothrix* | *beta_proteobacterium_*LF4_4*5* | [63] |
|  | ASV14347 | *Leptothrix* | *Burkholderiales_bacterium* | [62] |
|  | ASV14872 | *Leptothrix* | *Leptothrix_ginsengisoli* | [62] |
|  | ASV5023 | *Microbacterium* | *Microbacterium_lacus* | [69] |
|  | ASV10866 | *Mycobacterium* | *Mycobacterium_aromaticivorans_*  JS19b1_JCM_16368 | [75] |
|  | ASV7742 | *Paracoccus* | *Paracoccus_tibetensis* | [76] |
|  | ASV13560 | *Pseudomonas* | *Pseudomonas_alcaligenes* | [65] |
|  | ASV1838 | *Pseudomonas* | *Pseudomonas_anguilliseptica* | [66] |
|  | ASV7582 | *Pseudomonas* | *Pseudomonas_fluorescens* | [65] |
|  | ASV14322 | *Psychrobacter* | *Psychrobacter_psychrophilus* | [70] |
|  | ASV14461 | *Rheinheimera* | *Pararheinheimera_chironomi* | [67] |
|  | ASV13541 | *Rhodoferax* | *beta_proteobacterium_*HIBAF004 | [64] |
|  | ASV11672 | *Rhodoferax* | *beta_proteobacterium_*HIBAF005 | [64] |
|  | ASV1703 | *Rhodoferax* | *Curvibacter_putative_symbiont_of_*  *Hydra_magnipapillata* | [64] |
|  | ASV14882 | *Rhodoferax* | *Rhodoferax_saidenbachensis* | [64] |
|  | ASV1737 | *Rhodoferax* | *Rhodoferax_sediminis* | [64] |
|  | ASV13758 | *Roseateles* | *Roseateles_depolymerans* | [64] |
|  | ASV11580 | *Shewanella* | *Shewanella_putrefaciens* | [69] |
|  | ASV1819 | *Sphingomonas* | *Sphingomonas_fonticola* | [77] |
|  | ASV9681 | *Sphingomonas* | *Sphingomonas_gilva* | [71] |
|  | ASV10886 | *Sphingomonas* | *Sphingomonas_koreensis* | [77] |
|  | ASV7499 | *Sphingomonas* | *Sphingomonas_leidyi* | [77] |
|  | ASV4187 | *Streptococcus* | *Streptococcus_lutetiensis* | [72] |
|  | ASV11546 | *Undibacterium* | *Undibacterium_parvum* | [56] |
|  | ASV2225 | *Undibacterium* | *Undibacterium_pigrum* | [56] |
|  | ASV11624 | *Variovorax* | *Variovorax_boronicumulans* | [78] |
|  | ASV11728 | *Variovorax* | *Variovorax_ginsengisoli* | [64] |
| PLA_120 d | ASV807 | *Achromobacter* | *Achromobacter_arsenitoxydans* | [49] |
|  | ASV461 | *Acinetobacter* | *Acinetobacter_lwoffii* | [73] |
|  | ASV58 | *Bacteroides* | *Bacteroides_caecicola* | [52] |
|  | ASV273 | *Bacteroides* | *Bacteroides_caecigallinarum* | [52] |
|  | ASV12912 | *Bacteroides* | *Bacteroides_fragilis* | [52] |
|  | ASV37 | *Bacteroides* | *Bacteroides_gallinaceum* | [52] |
|  | ASV12784 | *Bacteroides* | *Bacteroides_*sp.*_Marseille_*P3166 | [52] |
|  | ASV318 | *Bacteroides* | *Bacteroides_togonis* | [52] |
|  | ASV190 | *Bacteroides* | *Bacteroides_vulgatus* | [52] |
|  | ASV2992 | *Bdellovibrio* | *Bdellovibrionales_bacterium_*Ga0074137 | [53] |
|  | ASV712 | *Leptothrix* | *beta_proteobacterium_*AAP51 | [63] |
|  | ASV17119 | *Rhodoferax* | *beta_proteobacterium_*HIBAF004 | [64] |
|  | ASV725 | *Brevundimonas* | *Brevundimonas_bacteroides* | [54] |
|  | ASV3127 | *Brevundimonas* | *Brevundimonas_nasdae* | [54] |
|  | ASV825 | *Brevundimonas* | *Brevundimonas_variabilis* | [54] |
|  | ASV1270 | *Comamonas* | *Comamonas_kerstersii* | [79] |
|  | ASV491 | *Rhodoferax* | *Curvibacter_putative_symbiont_of_*  *Hydra_magnipapillata* | [64] |
|  | ASV11911 | *Desulfovibrio* | *Desulfovibrio_bacterium_New_Zealand_D* | [52] |
|  | ASV718 | *Desulfovibrio* | *Desulfovibrio_piger* | [52] |
|  | ASV537 | *Desulfovibrio* | *Desulfovibrio_*sp*._Marseille_*P2429 | [52] |
|  | ASV358 | *Klebsiella* | *Klebsiella_pneumoniae* | [80] |
|  | ASV898 | *Ideonella* | *Leptothrix_ginsengisoli* | [60] |
|  | ASV3417 | *Mycobacterium* | *Mycobacterium_sphagni* | [75] |
|  | ASV280 | *Bacteroides* | *Phocaeicola_coprophilus* | [52] |
|  | ASV148 | *Bacteroides* | *Phocaeicola_plebeius* | [52] |
|  | ASV7 | *Bacteroides* | *Phocaeicola_salanitronis* | [52] |
|  | ASV1338 | *Ideonella* | *Rubrivivax_gelatinosus* | [60] |
|  | ASV1300 | *Sphingomonas* | *Sphingomonas_fonticola* | [77] |
|  | ASV9681 | *Sphingomonas* | *Sphingomonas_gilva* | [77] |
|  | ASV16188 | *Streptococcus* | *Streptococcus_canis* | [72] |
|  | ASV634 | *Streptococcus* | *Streptococcus_equinus* | [72] |
|  | ASV11235 | *Streptococcus* | *Streptococcus_pasteurianus* | [72] |
|  | ASV57 | *Bacteroides* | *unclassified_Bacteroides* | [52] |
|  | ASV286 | *Desulfovibrio* | *unclassified_Desulfovibrio* | [52] |
|  | ASV8818 | *Sphingomonas* | *unclassified_Sphingomonas* | [77] |
|  | ASV116 | *Bacteroides* | *uncultured_Bacteroidales_bacterium* | [52] |
|  | ASV81 | *Desulfovibrio* | *uncultured_Desulfovibrionaceae_bacterium* | [52] |
| PLA_210 d | ASV4136 | *Acinetobacter* | *Acinetobacter_lwoffii* | [73] |
|  | ASV184 | *Bacteroides* | *uncultured_Bacteroidales_bacterium* | [52] |
|  | ASV273 | *Bacteroides* | *Bacteroides_caecigallinarum* | [52] |
|  | ASV39 | *Bacteroides* | *Bacteroides_gallinaceum* | [52] |
|  | ASV4027 | *Bacteroides* | *Bacteroides_thetaiotaomicron* | [52] |
|  | ASV63 | *Bacteroides* | *Bacteroides_caecicola* | [52] |
|  | ASV6419 | *Bacteroides* | *Phocaeicola_dorei* | [52] |
|  | ASV6547 | *Bacteroides* | *Bacteroides_vulgatus* | [52] |
|  | ASV7 | *Bacteroides* | *Phocaeicola_salanitronis* | [52] |
|  | ASV7056 | *Bacteroides* | *unclassified_Bacteroides* | [52] |
|  | ASV7762 | *Bacteroides* | *Bacteroides_fragilis* | [52] |
|  | ASV7849 | *Bacteroides* | *Phocaeicola_vulgatus* | [52] |
|  | ASV1278 | *Bdellovibrio* | *unclassified_Bdellovibrio* | [53] |
|  | ASV1840 | *Bdellovibrio* | *Bdellovibrionales_bacterium_*RBG_16_40_8 | [53] |
|  | ASV5445 | *Bdellovibrio* | *Bdellovibrio_exovorus* | [53] |
|  | ASV5648 | *Bdellovibrio* | *Bdellovibrionales_bacterium_*Ga0074137 | [53] |
|  | ASV7739 | *Bdellovibrio* | *Bdellovibrionales_bacterium_*  RIFCSPHIGHO2_01_FULL_40_29 | [53] |
|  | ASV2840 | *Brevundimonas* | *Brevundimonas_denitrificans* | [54] |
|  | ASV3129 | *Brevundimonas* | *Brevundimonas_bacteroides* | [54] |
|  | ASV4007 | *Brevundimonas* | *Brevundimonas_variabilis* | [54] |
|  | ASV6238 | *Brevundimonas* | *Brevundimonas_aveniformis* | [54] |
|  | ASV6241 | *Brevundimonas* | *Brevundimonas_intermedia* | [54] |
|  | ASV6289 | *Brevundimonas* | *Brevundimonas_subvibrioides* | [54] |
|  | ASV6316 | *Brevundimonas* | *Brevundimonas_fluminis* | [54] |
|  | ASV7601 | *Brevundimonas* | *Brevundimonas_humi* | [54] |
|  | ASV7768 | *Chitinimonas* | *Chitinimonas_viridis* | [56] |
|  | ASV2282 | *Desulfovibrio* | *unclassified_Desulfovibrio* | [52] |
|  | ASV7023 | *Desulfovibrio* | *uncultured_Desulfovibrionaceae_bacterium* | [52] |
|  | ASV1695 | *Flavobacterium* | *Flavobacterium_collinsii* | [58] |
|  | ASV1700 | *Flavobacterium* | *Flavobacterium_anseonense* | [58] |
|  | ASV2126 | *Flavobacterium* | *Flavobacterium_terrigena* | [58] |
|  | ASV2800 | *Flavobacterium* | *Flavobacterium_tructae* | [58] |
|  | ASV5558 | *Flavobacterium* | *Flavobacterium_nackdongense* | [58] |
|  | ASV6582 | *Flavobacterium* | *Flavobacterium_cheonanense* | [58] |
|  | ASV5871 | *Halomonas* | *Candidatus_Halomonas_phosphatis* | [59] |
|  | ASV1660 | *Ideonella* | *Rubrivivax_gelatinosus* | [61] |
|  | ASV1683 | *Ideonella* | *beta_proteobacterium_*A0640 | [60] |
|  | ASV1733 | *Ideonella* | *Aquincola_tertiaricarbonis* | [60] |
|  | ASV1896 | *Ideonella* | *Hyalella_azteca* | [60] |
|  | ASV2005 | *Ideonella* | *Ideonella_dechloratans* | [60] |
|  | ASV898 | *Ideonella* | *Leptothrix_ginsengisoli* | [60] |
|  | ASV1624 | *Leptothrix* | *beta_proteobacterium_*AAP51 | [63] |
|  | ASV1687 | *Leptothrix* | *Burkholderiales_bacterium* | [63] |
|  | ASV1789 | *Leptothrix* | *beta_proteobacterium_*LF4_45 | [63] |
|  | ASV3322 | *Leptothrix* | *Leptothrix_mobilis* | [63] |
|  | ASV5343 | *Leptothrix* | *beta_proteobacterium_*AAP121 | [62] |
|  | ASV7758 | *Leptothrix* | *Leptothrix_cholodnii* | [63] |
|  | ASV5650 | *Leucobacter* | *Leucobacter_komagatae* | [81] |
|  | ASV5023 | *Microbacterium* | *Microbacterium_lacus* | [69] |
|  | ASV3417 | *Mycobacterium* | *Mycobacterium_sphagni* | [75] |
|  | ASV5714 | *Paracoccus* | *Paracoccus_tibetensis* | [76] |
|  | ASV1638 | *Pseudomonas* | *Pseudomonas_monteilii* | [65] |
|  | ASV1672 | *Pseudomonas* | *Pseudomonas_laurentiana* | [65] |
|  | ASV1704 | *Pseudomonas* | *Pseudomonas_anguilliseptica* | [65] |
|  | ASV1900 | *Pseudomonas* | *Pseudomonas_fluorescens* | [65] |
|  | ASV5346 | *Pseudomonas* | *Pseudomonas_putida* | [66] |
|  | ASV5573 | *Pseudomonas* | *Pseudomonas_mendocina* | [65] |
|  | ASV7863 | *Pseudomonas* | *Pseudomonas_azotoformans* | [65] |
|  | ASV2076 | *Rheinheimera* | *Rheinheimera_tangshanensis* | [67] |
|  | ASV4632 | *Rheinheimera* | *Spumella_vulgaris* | [67] |
|  | ASV5334 | *Rheinheimera* | *Pararheinheimera_arenilitoris* | [67] |
|  | ASV6437 | *Rheinheimera* | *Pararheinheimera_chironomi* | [67] |
|  | ASV1737 | *Rhodoferax* | *Rhodoferax_sediminis* | [64] |
|  | ASV491 | *Rhodoferax* | *Curvibacter_putative_symbiont_of_*  *Hydra_magnipapillata* | [64] |
|  | ASV5460 | *Rhodoferax* | *Rhodoferax_ferrireducens* | [64] |
|  | ASV7649 | *Rhodoferax* | *Rhodoferax_saidenbachensis* | [64] |
|  | ASV5335 | *Shewanella* | *Shewanella_putrefaciens* | [70] |
|  | ASV1674 | *Sphingomonas* | *Sphingomonas_lacunae* | [71] |
|  | ASV7499 | *Sphingomonas* | *Sphingomonas_leidyi* | [71] |
|  | ASV7751 | *Sphingomonas* | *Sphingomonas_gilva* | [77] |
|  | ASV7752 | *Sphingomonas* | *unclassified_Sphingomonas* | [77] |
|  | ASV7791 | *Sphingomonas* | *Sphingomonas_fonticola* | [71] |
|  | ASV634 | *Streptococcus* | *Streptococcus_equinus* | [72] |
|  | ASV6468 | *Streptococcus* | *Streptococcus_infantis* | [72] |
|  | ASV5645 | *Undibacterium* | *beta_proteobacterium_*Wuba70 | [56] |
|  | ASV7875 | *Undibacterium* | *Undibacterium_seohonense* | [56] |
|  | ASV2744 | *Variovorax* | *Variovorax_ginsengisoli* | [78] |

# Table S8 Reported MP-degrading strains from published studies.

| **Microorganism** | **Degradation conditions** | **Weight loss** | **Reference** |
| --- | --- | --- | --- |
| *Pseudomonas* sp. ADL15 (KX812776) | 40 days | 17.3% | [82] |
| *Rhodococcus* sp. ADL36 (KX812777) | 40 days | 7.3% | [82] |
| *Bacillus lentus* (AB021189) | 40 days | 40.0% | [83] |
| *Bacillus licheniformis* (AF478085) | 40 days | 34.1% | [83] |
| *Staphylococcus epidermidis* (AE015931) | 40 days | 9.9% | [83] |
| *Klebsiella pneumoniae* (MH021669) | 40 days | 3.8% | [83] |
| *Escherichia coli* (KX609714) | 40 days | 3.5% | [83] |
| *Lysinibacillus* sp. (JJY0216) | 26 days | 4.0% | [84] |
| *Bacillus cereus* | 40 days | 12.0% | [85] |
| *Sporosarcina globispora* | 40 days | 11% | [85] |
| *Rhodococcus* sp. strain 36 | 40 days | 6.4% | [86] |
| *Bacillus* sp. strain 27 | 40 days | 4.0% | [86] |
| *Staphylococcus* sp. | 180 days | 9.5% | [87] |
| *Bacillus cereus* (MW881525.1) | 21 days | 63.1% | [88] |
| *Bacillus paramycoides* | 21 days | 79.0% | [88] |
| *Aneurinibacillus aneurinilyticus bt*DSCE01 (MF359591.1) | 140 days, 50°C | 24.4% | [89] |
| *Brevibacillus agri bt*DSCE02 (MF359592.1) | 140 days, 50°C | 27.0% | [89] |
| *Brevibacillus* sp. btDSCE03 (MF359593.1) | 140 days, 50°C | 22.8% | [89] |
| *Brevibacillus brevis bt*DSCE04 (MF359594.1) | 140 days, 50°C | 23.4% | [89] |
| *Pseudoalteromonas lipolytica* | 80 days, 15 °C | 1.3% | [90] |
| *Pseudoalteromonas tetraodonis* | 80 days, 15 °C | 0.7% | [90] |
| *Bacillus tropicus* (OP604494) | 28 days | 51.5% | [91] |
| *Bacillus cereus* (OP604518) | 28 days | 47.5% | [91] |
| *Stenotrophomonas acidaminiphila* (OP604537) | 28 days | 33.0% | [91] |
| *Brucella pseudintermedia* (OP604555) | 28 days | 28.5% | [91] |
| *Bacillus cereus* (OP604570) | 28 days | 35.5% | [91] |
| *Bacillus safensis* | 30 days | 8.0% | [92] |
| *Bacillus brevis* | 20 days, 58°C | - | [93] |
| *Bacillus stearothermophilus* | 20 days | 25.0% | [94] |
| *Bacillus licheniformis* | 5 months, 32°C | 40.0% | [95] |
| *Geobacillus* | 20 days, 32°C | - | [96] |
| *Pseudomonas* sp. strain DS04-T(EU362995.1) | 15 days | 55.0% | [97] |
| *Stenotrophomonas maltophilia* LB 2-3 | 40 days, 37°C | - | [98] |
| *Bacillus licheniformis* (ATCC14580) | 30 days, 37°C | 2.8% | [99] |
| *Bacillus subtilis* (ATCC6051) | 30 days, 37°C | 3.5% | [99] |
| *Priestia* sp. (DG69) | 30°C | - | [100] |
| *Neobacillus* sp. (DG40) | 30°C | - | [100] |
| *Priestia aryabhattai* VT 3.12 | 28 days | 69.0% | [101] |
| *Bacillus pseudomycoides* VT 3.15 | 28 days | 66.0% | [101] |
| *Bacillus pumilus* VT 3.16 | 28 days | 64.0% | [101] |
| *Ideonella sakaiensis*201-F6 | - | 60.0% | [60] |
| *Escherichia coli* | 72 h | 1.0-2.0% | [102] |
| *Bacillus australimaris* (CGK221) | 90 days | - | [103] |
| *Bacillus safensis* (CGK192) | 90 days | - | [103] |
| *Bacillus* sp. (BCBT21) | 55°C | 11.0% | [104] |
| *Bacillus subtilis* (WB600) | - | - | [105] |
| *Bacillus subtilis strain* (KM411502.1) | 49 days | 70.0% | [106] |
| *Bacillus subtilis* (TB8) | 60 days | 16.0% | [107] |
| *Bacillus flexus* | 45 days | 13.1–18.6% | [108] |

# Table S9 Reaction orders of various chemical bonds of PP after 45 days of co-culture with PLA_strain_.

| **Peak (cm**^–^**^1^)** | **Band assignments** | **Sign** | | | | |
| --- | --- | --- | --- | --- | --- | --- |
|  |  | **841** | **1163** | **1375** | **1741** | **3393** |
| 841 | ‒CH | +(–) | +(+) | +(+) | +(+) | +(–) |
| 1163 | C‒O |  | +(+) | +(+) | +(+) | +(–) |
| 1375 | O‒C=O |  |  | +(–) | +(–) | +(–) |
| 1741 | C=O |  |  |  | +(–) | +(–) |
| 3393 | ‒OH |  |  |  |  | +(+) |

Note: The symbol outside the brackets represents synchronous results, and those in the brackets indicate asynchronous results.

# Table S10 Reaction orders of various chemical bonds of PLA after 45 days of co-culture with PLA_strain_.

| **Peak (cm**^–^**^1^)** | **Band assignments** | **Sign** | | | | | |
| --- | --- | --- | --- | --- | --- | --- | --- |
|  |  | **1080** | **1126** | **1177** | **1754** | **2948** | **3502** |
| 1080 | ‒CH_2_‒OH | +(–) | +(–) | +(–) | +(–) | +(–) | +(–) |
| 1126 | ‒CH‒OH |  | +(+) | +(–) | +(–) | +(–) | +(–) |
| 1177 | ‒C-OH |  |  | +(+) | +(–) | +(–) | +(–) |
| 1754 | ‒C=O |  |  |  | +(+) | +(–) | +(–) |
| 2948 | ‒CH stretching |  |  |  |  | +(+) | +(+) |
| 3502 | ‒OH |  |  |  |  |  | +(–) |

Note: The symbol outside the brackets represents synchronous results, and those in the brackets indicate asynchronous results.

**Table S11 The particle size of PP and PLA during 45 days of co-culture.**

|  | **Treatment** | **Time (d)** | **Avg (mm)** | **Max (mm)** | **Min (mm)** | **Med (mm)** |
| --- | --- | --- | --- | --- | --- | --- |
| PP | CK | 0 | 0.27 | 0.45 | 0.08 | 0.27 |
|  |  | 20 | 0.27 | 0.44 | 0.15 | 0.27 |
|  |  | 45 | 0.27 | 0.38 | 0.15 | 0.27 |
|  | PP_strain_ | 20 | 0.23 | 0.53 | 0.05 | 0.24 |
|  |  | 45 | 0.21 | 0.43 | 0.04 | 0.21 |
|  | PLA_strain_ | 20 | 0.26 | 0.42 | 0.09 | 0.26 |
|  |  | 45 | 0.26 | 0.40 | 0.13 | 0.26 |
|  | PP_microbial consortium_ | 20 | 0.25 | 0.37 | 0.09 | 0.35 |
|  |  | 45 | 0.23 | 0.41 | 0.04 | 0.24 |
| PLA | CK | 0 | 0.27 | 0.46 | 0.12 | 0.27 |
|  |  | 20 | 0.27 | 0.53 | 0.09 | 0.27 |
|  |  | 45 | 0.27 | 0.55 | 0.07 | 0.27 |
|  | PLA_strain_ | 20 | 0.24 | 0.53 | 0.05 | 0.24 |
|  |  | 45 | 0.21 | 0.46 | 0.05 | 0.21 |
|  | PP_strain_ | 20 | 0.26 | 0.47 | 0.04 | 0.26 |
|  |  | 45 | 0.25 | 0.45 | 0.08 | 0.25 |
|  | PLA_microbial consortium_ | 20 | 0.24 | 0,48 | 0.03 | 0.24 |
|  |  | 45 | 0.22 | 0.45 | 0.05 | 0.22 |

Note: Avg, Max, Min, and Med denote average, maximum, minimum, and median particle sizes of MPs, respectively.

# Table S12 Classification of key functional genes annotated in PP_strain_ and PLA_strain_.

|  | **#gene_ID** | **KO** | **KO_function** | **Pathway_ID** | **Pathway_name** | **Enzyme** | **E-value** | **Query-cover** | **score** |
| --- | --- | --- | --- | --- | --- | --- | --- | --- | --- |
| PP | GE000163 | K00632 | Acetyl-CoA acyltransferase | ko01130; ko00071; ko01212; ko00592; ko00281; ko01120; ko00280; ko01110; ko00362; ko01100 | Biosynthesis of antibiotics; fatty acid degradation; fatty acid metabolism; alpha-linolenic acid metabolism; geraniol degradation; microbial metabolism in diverse environments; valine, leucine and isoleucine degradation; biosynthesis of secondary metabolites; benzoate degradation; metabolic pathways | EC 2.3.1.16 | 7.2e-212 | 100.00 | 741 |
|  | GE000164 | K07516 | 3-hydroxyacyl-CoA dehydrogenase | ko00650; ko00362; ko01120; ko01200; ko01100; ko01212; ko00071 | Butanoate metabolism; benzoate degradation; microbial metabolism in diverse environments; carbon metabolism; metabolic pathways; fatty acid metabolism; fatty acid degradation | EC 1.1.1.35 | 0.0e+00 | 100.00 | 1462 |
|  | GE000172 | K00059 | 3-oxoacyl-[acyl-carrier protein] reductase | ko01100; ko01040; ko00061; ko01212; ko00780 | Metabolic pathways; biosynthesis of unsaturated fatty acids; fatty acid biosynthesis; fatty acid metabolism; biotin metabolism | EC 1.1.1.100 | 7.7e-111 | 100.00 | 405 |
|  | GE000244 | K00059 | 3-oxoacyl-[acyl-carrier protein] reductase | ko01212; ko00061; ko01040; ko01100; ko00780 | Fatty acid metabolism; Fatty acid biosynthesis; Biosynthesis of unsaturated fatty acids; Metabolic pathways; Biotin metabolism | EC 1.1.1.100 | 7.9e-138 | 100.00 | 495 |
|  | GE000598 | K04564 | Superoxide dismutase, Fe-Mn family | -- | -- | EC 1.15.1.1 | 4.7e-114 | 100.00 | 416 |
|  | GE000689 | K00626 | Acetyl-CoA C-acetyltransferase | ko00640; ko00900; ko01100; ko01200; ko00380; ko01120; ko00650; ko00280; ko00310; ko02020; ko01110; ko01212; ko00620; ko01130; ko00072; ko00071; ko00362; ko00630 | Propanoate metabolism; terpenoid backbone biosynthesis; metabolic pathways; carbon metabolism; tryptophan metabolism; microbial metabolism in diverse environments; butanoate metabolism; valine, leucine and isoleucine degradation; lysine degradation; two-component system; biosynthesis of secondary metabolites; fatty acid metabolism; pyruvate metabolism; biosynthesis of antibiotics; synthesis and degradation of ketone bodies; fatty acid degradation; benzoate degradation; glyoxylate and dicarboxylate metabolism | EC 2.3.1.9 | 4.4e-201 | 100.00 | 706 |
|  | GE000996 | K00164 | 2-oxoglutarate dehydrogenase E1 component | ko01100; ko01200; ko01120; ko00380; ko00310; ko00020; ko01130; ko01110 | Metabolic pathways; carbon metabolism; microbial metabolism in diverse environments; tryptophan metabolism; lysine degradation; TCA cycle; biosynthesis of antibiotics; biosynthesis of secondary metabolites | EC 1.2.4.2 | 0.0e+00 | 100.00 | 1854 |
|  | GE001002 | K00128 | Aldehyde dehydrogenase (NAD^+^) | ko00561; ko00071; ko00330; ko01130; ko01110; ko00040; ko01100; ko00010; ko00340; ko00053; ko00280; ko01120; ko00620; ko00310; ko00410; ko00380; ko00625 | Glycerolipid metabolism; fatty acid degradation; arginine and proline metabolism; biosynthesis of antibiotics; biosynthesis of secondary metabolites; pentose and glucuronate interconversions; metabolic pathways; glycolysis/gluconeogenesis; histidine metabolism; ascorbate and aldarate metabolism; valine, leucine and isoleucine degradation; microbial metabolism in diverse environments; pyruvate metabolism; lysine degradation; beta-alanine metabolism; tryptophan metabolism; chloroalkane and chloroalkene degradation | EC 1.2.1.3 | 8.1e-285 | 100.00 | 984 |
|  | GE001237 | K00059 | 3-oxoacyl-[acyl-carrier protein] reductase | ko00780; ko00061; ko01212; ko01100; ko01040 | Biotin metabolism; fatty acid biosynthesis; fatty acid metabolism; metabolic pathways; biosynthesis of unsaturated fatty acids | EC 1.1.1.100 | 2.3e-115 | 100.00 | 420 |
|  | GE001332 | K00059 | 3-oxoacyl-[acyl-carrier protein] reductase | ko01212; ko00780; ko01100; ko00061; ko01040 | Fatty acid metabolism; biotin metabolism; metabolic pathways; fatty acid biosynthesis; biosynthesis of unsaturated fatty acids | EC 1.1.1.100 | 2.9e-126 | 100.00 | 456 |
|  | GE001570 | K00128 | Aldehyde dehydrogenase (NAD^+^) | ko00410; ko00310; ko01130; ko00040; ko00620; ko00280; ko01110; ko00380; ko00330; ko01100; ko00010; ko00340; ko01120; ko00625; ko00053; ko00071; ko00561 | beta-alanine metabolism; lysine degradation; biosynthesis of antibiotics; pentose and glucuronate interconversions; pyruvate metabolism; valine, leucine and isoleucine degradation; biosynthesis of secondary metabolites; tryptophan metabolism; arginine and proline metabolism; metabolic pathways; glycolysis/gluconeogenesis; histidine metabolism; microbial metabolism in diverse environments; chloroalkane and chloroalkene degradation; ascorbate and aldarate metabolism; fatty acid degradation; glycerolipid metabolism | EC 1.2.1.3 | 4.6e-242 | 100.00 | 842 |
|  | GE001774 | K00128 | Aldehyde dehydrogenase (NAD^+^) | ko00053; ko00410; ko00620; ko00310; ko00340; ko00040; ko00010; ko00561; ko00380; ko01110; ko01120; ko00280; ko01130; ko00330; ko01100; ko00071; ko00625 | Ascorbate and aldarate metabolism; beta-alanine metabolism; pyruvate metabolism; lysine degradation; histidine metabolism; pentose and glucuronate interconversions; glycolysis/gluconeogenesis; glycerolipid metabolism; tryptophan metabolism; biosynthesis of secondary metabolites; microbial metabolism in diverse environments; valine, leucine and isoleucine degradation; biosynthesis of antibiotics; arginine and proline metabolism; metabolic pathways; fatty acid degradation; chloroalkane and chloroalkene degradation | EC 1.2.1.3 | 5.8e-242 | 100.00 | 842 |
|  | GE001975 | K00128 | Aldehyde dehydrogenase (NAD^+^) | ko00620; ko00071; ko00340; ko00310; ko00380; ko01130; ko00053; ko00040; ko00010; ko01110; ko00625; ko01120; ko00280; ko01100; ko00330; ko00561; ko00410 | Pyruvate metabolism; fatty acid degradation; histidine metabolism; lysine degradation; tryptophan metabolism; biosynthesis of antibiotics; ascorbate and aldarate metabolism; pentose and glucuronate interconversions; glycolysis/gluconeogenesis; biosynthesis of secondary metabolites; chloroalkane and chloroalkene degradation; microbial metabolism in diverse environments; valine, leucine and isoleucine degradation; metabolic pathways; arginine and proline metabolism; glycerolipid metabolism; beta-alanine metabolism | EC 1.2.1.3 | 2.1e-264 | 99.00 | 916 |
|  | GE002072 | K00121 | S-(hydroxymethyl)glutathione dehydrogenase/alcohol dehydrogenase | ko01100; ko00071; ko00625; ko00350; ko00626; ko01110; ko00680; ko01220; ko00010; ko01200; ko01130; ko01120 | Metabolic pathways; fatty acid degradation; chloroalkane and chloroalkene degradation; tyrosine metabolism; naphthalene degradation; biosynthesis of secondary metabolites; methane metabolism; degradation of aromatic compounds; glycolysis/gluconeogenesis; carbon metabolism; biosynthesis of antibiotics; microbial metabolism in diverse environments | EC 1.1.1.284 1.1.1.1 | 3.6e-200 | 100.00 | 703 |
|  | GE002736 | K01897 | Long-chain acyl-CoA synthetase | ko01100; ko02024; ko00071; ko00061; ko01212 | Metabolic pathways; quorum sensing; fatty acid degradation; fatty acid biosynthesis; fatty acid metabolism | EC 6.2.1.3 | 3.6e-229 | 98.96 | 799 |
|  | GE002737 | K00626 | Acetyl-CoA C-acetyltransferase | ko00310; ko01212; ko00380; ko01130; ko00280; ko01110; ko00630; ko00362; ko00071; ko00640; ko01100; ko00900; ko00650; ko00072; ko00620; ko01120; ko01200; ko02020 | Lysine degradation; fatty acid metabolism; tryptophan metabolism; biosynthesis of antibiotics; valine, leucine and isoleucine degradation; biosynthesis of secondary metabolites; glyoxylate and dicarboxylate metabolism; benzoate degradation; fatty acid degradation; propanoate metabolism; metabolic pathways; terpenoid backbone biosynthesis; butanoate metabolism; synthesis and degradation of ketone bodies; pyruvate metabolism; microbial metabolism in diverse environments; carbon metabolism; two-component system | EC 2.3.1.9 | 5.3e-177 | 99.73 | 625 |
|  | GE002745 | K01897 | Long-chain acyl-CoA synthetase | ko01100; ko00071; ko00061; ko01212; ko02024 | Metabolic pathways; fatty acid degradation; fatty acid biosynthesis; fatty acid metabolism; quorum sensing | EC 6.2.1.3 | 3.0e-282 | 99.81 | 976 |
|  | GE002824 | K14338 | Cytochrome P450/NADPH-cytochrome P450 reductase | ko00627; ko01120; ko00380; ko00071 | Aminobenzoate degradation; microbial metabolism in diverse environments; tryptophan metabolism; fatty acid degradation. | EC 1.14.14.1 1.6.2.4 | 0.0e+00 | 100.00 | 1892 |
|  | GE002957 | K00240 | Succinate dehydrogenase/fumarate reductase, iron-sulfur subunit | ko00190; ko01110; ko01200; ko00020; ko01100; ko01120; ko01130; ko00650 | Oxidative phosphorylation; biosynthesis of secondary metabolites; carbon metabolism; TCA cycle; metabolic pathways; microbial metabolism in diverse environments; biosynthesis of antibiotics; butanoate metabolism | EC 1.3.5.1 1.3.5.4 | 2.0e-146 | 100.00 | 523 |
|  | GE002958 | K00239 | Succinate dehydrogenase/fumarate reductase, flavoprotein subunit | ko01100; ko00190; ko00020; ko01200; ko01110; ko01120; ko00650; ko01130 | Metabolic pathways; oxidative phosphorylation; TCA cycle; carbon metabolism; biosynthesis of secondary metabolites; microbial metabolism in diverse environments; butanoate metabolism; biosynthesis of antibiotics | EC 1.3.5.1 1.3.5.4 | 0.0e+00 | 100.00 | 1185 |
|  | GE002967 | K13767 | Enoyl-CoA hydratase | ko01212; ko00362; ko01120; ko00071; ko01100 | Fatty acid metabolism; benzoate degradation; microbial metabolism in diverse environments; fatty acid degradation; metabolic pathways | EC 4.2.1.17 | 1.4e-131 | 100.00 | 474 |
|  | GE002969 | K01897 | Long-chain acyl-CoA synthetase | ko00061; ko00071; ko01212; ko02024; ko01100 | Fatty acid biosynthesis; fatty acid degradation; fatty acid metabolism; quorum sensing; metabolic pathways | EC 6.2.1.3 | 2.2e-311 | 100.00 | 1072 |
|  | GE003047 | K00059 | 3-oxoacyl-[acyl-carrier protein] reductase | ko01100; ko01212; ko00780; ko00061; ko01040 | Metabolic pathways; fatty acid metabolism; biotin metabolism; fatty acid biosynthesis; biosynthesis of unsaturated fatty acids | EC 1.1.1.100 | 5.5e-136 | 100.00 | 489 |
|  | GE003229 | K00059 | 3-oxoacyl-[acyl-carrier protein] reductase | ko00061; ko01040; ko00780; ko01100; ko01212 | Fatty acid biosynthesis; biosynthesis of unsaturated fatty acids; biotin metabolism; metabolic pathways; fatty acid metabolism | EC 1.1.1.100 | 6.1e-84 | 99.19 | 316 |
|  | GE003344 | K14338 | Cytochrome P450/NADPH-cytochrome P450 reductase | ko00627; ko01120; ko00380; ko00071 | Aminobenzoate degradation; microbial metabolism in diverse environments; tryptophan metabolism; fatty acid degradation | EC 1.14.14.1 1.6.2.4 | 0.0e+00 | 100.00 | 2019 |
|  | GE003354 | K00128 | Aldehyde dehydrogenase (NAD^+^) | ko00380; ko00620; ko01110; ko00071; ko01120; ko00340; ko00010; ko00053; ko00280; ko00410; ko00310; ko00625; ko01100; ko00040; ko00561; ko00330; ko01130 | Tryptophan metabolism; pyruvate metabolism; biosynthesis of secondary metabolites; fatty acid degradation; microbial metabolism in diverse environments; histidine metabolism; glycolysis/gluconeogenesis; ascorbate and aldarate metabolism; valine, leucine and isoleucine degradation; beta-alanine metabolism; lysine degradation; chloroalkane and chloroalkene degradation; metabolic pathways; pentose and glucuronate interconversions; glycerolipid metabolism; arginine and proline metabolism; biosynthesis of antibiotics | EC 1.2.1.3 | 8.2e-274 | 100.00 | 948 |
|  | GE003561 | K04091 | Alkanesulfonate monooxygenase | ko00920 | Sulfur metabolism | EC 1.14.14.5 | 1.7e-205 | 100.00 | 720 |
|  | GE003565 | K03781 | Catalase | ko00380; ko00630; ko01110; ko01200; ko01130 | Tryptophan metabolism; glyoxylate and dicarboxylate metabolism; biosynthesis of secondary metabolites; carbon metabolism; biosynthesis of antibiotics | EC 1.11.1.6 | 7.1e-286 | 99.79 | 988 |
|  | GE003571 | K00121 | S-(hydroxymethyl)glutathione dehydrogenase/alcohol dehydrogenase | ko01100; ko01220; ko00071; ko01130; ko00626; ko00350; ko00680; ko01110; ko00010; ko01200; ko00625; ko01120 | Metabolic pathways; degradation of aromatic compounds; fatty acid degradation; biosynthesis of antibiotics; naphthalene degradation; tyrosine metabolism; methane metabolism; biosynthesis of secondary metabolites; glycolysis/gluconeogenesis; carbon metabolism; chloroalkane and chloroalkene degradation; microbial metabolism in diverse environments | EC 1.1.1.284 1.1.1.1 | 7.9e-208 | 100.00 | 728 |
|  | GE003945 | K00059 | 3-oxoacyl-[acyl-carrier protein] reductase | ko01040; ko00061; ko01212; ko00780; ko01100 | Biosynthesis of unsaturated fatty acids; fatty acid biosynthesis; fatty acid metabolism; biotin metabolism; metabolic pathways | EC 1.1.1.100 | 2.6e-120 | 94.38 | 437 |
| PLA | GE000164 | K00104 | Glycolate oxidase | ko00630; ko01130; ko01120; ko01100; ko01110 | Glyoxylate and dicarboxylate metabolism; biosynthesis of antibiotics; microbial metabolism in diverse environments; metabolic pathways; biosynthesis of secondary metabolites | EC 1.1.3.15 | 7.5e-235 | 98.98 | 818 |
|  | GE000505 | K01916 | NAD^+^ synthase | ko01100; ko00760 | Metabolic pathways; nicotinate and nicotinamide metabolism | EC 6.3.1.5 | 4.2e-126 | 98.91 | 456 |
|  | GE000617 | K00161 | Pyruvate dehydrogenase E1 component alpha subunit | ko01100; ko01130; ko00620; ko01110; ko01200; ko01120; ko00010; ko00020 | Metabolic pathways; biosynthesis of antibiotics; pyruvate metabolism; biosynthesis of secondary metabolites; carbon metabolism; microbial metabolism in diverse environments; glycolysis/gluconeogenesis; TCA cycle | EC 1.2.4.1 | 2.1e-197 | 100.00 | 693 |
|  | GE000618 | K00162 | Pyruvate dehydrogenase E1 component beta subunit | ko00010; ko01130; ko01120; ko01110; ko01200; ko00620; ko00020; ko01100 | Glycolysis/gluconeogenesis; biosynthesis of antibiotics; microbial metabolism in diverse environments; biosynthesis of secondary metabolites; carbon metabolism; pyruvate metabolism; TCA cycle; metabolic pathways | EC 1.2.4.1 | 1.2e-172 | 100.00 | 611 |
|  | GE000974 | K07248 | Lactaldehyde dehydrogenase/glycolaldehyde dehydrogenase | ko00630; ko01120; ko00620 | Glyoxylate and dicarboxylate metabolism; microbial metabolism in diverse environments; pyruvate metabolism | EC 1.2.1.22 1.2.1.21 | 2.3e-207 | 99.59 | 727 |
|  | GE001006 | K00128 | Aldehyde dehydrogenase (NAD^+^) | ko00410; ko00620; ko00561; ko01120; ko00071; ko01110; ko00625; ko00330; ko00053; ko00380; ko00010; ko01100; ko00040; ko00310; ko00280; ko00340; ko01130 | Beta-alanine metabolism; pyruvate metabolism; glycerolipid metabolism; microbial metabolism in diverse environments; fatty acid degradation; biosynthesis of secondary metabolites; chloroalkane and chloroalkene degradation; arginine and proline metabolism; ascorbate and aldarate metabolism; tryptophan metabolism; glycolysis/gluconeogenesis; metabolic pathways; pentose and glucuronate interconversions; lysine degradation; valine, leucine and isoleucine degradation; histidine metabolism; biosynthesis of antibiotics | EC 1.2.1.3 | 1.7e-207 | 100.00 | 727 |
|  | GE001275 | K03928 | Carboxylesterase | -- | -- | EC 3.1.1.1 | 1.9e-93 | 97.98 | 347 |
|  | GE001686 | K03928 | Carboxylesterase | -- | -- | EC 3.1.1.1 | 5.5e-112 | 98.05 | 409 |
|  | GE001817 | K00925 | Acetate kinase | ko00430; ko00620; ko01120; ko01100; ko01200; ko00680; ko00640 | Taurine and hypotaurine metabolism; pyruvate metabolism; microbial metabolism in diverse environments; metabolic pathways; carbon metabolism; methane metabolism; propanoate metabolism | EC 2.7.2.1 | 2.6e-209 | 100.00 | 733 |
|  | GE002154 | K00016 | L-lactate dehydrogenase | ko01130; ko01120; ko00010; ko01100; ko00620; ko01110; ko00640; ko00270 | Biosynthesis of antibiotics; microbial metabolism in diverse environments; glycolysis/gluconeogenesis; metabolic pathways; pyruvate metabolism; biosynthesis of secondary metabolites; propanoate metabolism; cysteine and methionine metabolism | EC 1.1.1.27 | 1.1e-130 | 98.08 | 471 |
|  | GE002301 | K00016 | L-lactate dehydrogenase | ko01110; ko00640; ko01120; ko01100; ko00620; ko01130; ko00010; ko00270 | Biosynthesis of secondary metabolites; propanoate metabolism; microbial metabolism in diverse environments; metabolic pathways; pyruvate metabolism; biosynthesis of antibiotics; glycolysis/gluconeogenesis; cysteine and methionine metabolism | EC 1.1.1.27 | 5.1e-104 | 98.71 | 383 |
|  | GE002717 | K04771 | Serine protease Do | ko02020 | Two-component system | EC 3.4.21.107 | 1.2e-138 | 84.88 | 498 |
|  | GE002851 | K00128 | Aldehyde dehydrogenase (NAD^+^) | ko00310; ko00280; ko00010; ko00340; ko01100; ko00071; ko01110; ko00053; ko00620; ko00410; ko00625; ko01130; ko00330; ko00561; ko00380; ko00040; ko01120 | Lysine degradation; valine, leucine and isoleucine degradation; glycolysis/gluconeogenesis; histidine metabolism; metabolic pathways; fatty acid degradation; biosynthesis of secondary metabolites; ascorbate and aldarate metabolism; pyruvate metabolism; beta-alanine metabolism; chloroalkane and chloroalkene degradation; biosynthesis of antibiotics; arginine and proline metabolism; glycerolipid metabolism; tryptophan metabolism; pentose and glucuronate interconversions; microbial metabolism in diverse environments | EC 1.2.1.3 | 7.2e-189 | 100.00 | 665 |
|  | GE002890 | K00016 | L-lactate dehydrogenase | ko01100; ko01110; ko00640; ko00010; ko00620; ko01130; ko00270; ko01120 | Metabolic pathways; biosynthesis of secondary metabolites; propanoate metabolism; glycolysis/gluconeogenesis; pyruvate metabolism; biosynthesis of antibiotics; cysteine and methionine metabolism; microbial metabolism in diverse environments | EC 1.1.1.27 | 1.8e-75 | 97.15 | 288 |
|  | GE003126 | K04771 | Serine protease Do | ko02020 | Two-component system | EC 3.4.21.107 | 8.9e-157 | 99.50 | 558 |
|  | GE003177 | K00625 | Phosphate acetyltransferase | ko01200; ko00640; ko00430; ko00680; ko01100; ko00620; ko01120 | Carbon metabolism; propanoate metabolism; taurine and hypotaurine metabolism; methane metabolism; metabolic pathways; pyruvate metabolism; microbial metabolism in diverse environments | EC 2.3.1.8 | 7.1e-157 | 100.00 | 558 |
|  | GE003337 | K03928 | Carboxylesterase | -- | -- | EC 3.1.1.1 | 2.8e-129 | 100.00 | 466 |
|  | GE003644 | K01046 | Triacylglycerol lipase | ko01100; ko00561 | Metabolic pathways; glycerolipid metabolism | EC 3.1.1.3 | 2.2e-105 | 100.00 | 387 |
|  | GE000974 | K07248 | Lactaldehyde dehydrogenase/glycolaldehyde dehydrogenase | ko00630; ko01120; ko00620 | Glyoxylate and dicarboxylate metabolism; microbial metabolism in diverse environments; pyruvate metabolism | EC 1.2.1.22 1.2.1.21 | 2.3e-207 | 99.59 | 727 |
|  | GE003356 | K00632 | Acetyl-CoA acyltransferase | ko01120; ko00281; ko01130; ko00280; ko01110; ko01212; ko00071; ko00592; ko00362; ko01100 | Microbial metabolism in diverse environments; geraniol degradation; biosynthesis of antibiotics; valine, leucine and isoleucine degradation; biosynthesis of secondary metabolites; fatty acid metabolism; fatty acid degradation; alpha-linolenic acid metabolism; benzoate degradation; metabolic pathways | EC 2.3.1.16 | 2.2e-200 | 100.00 | 703 |

**References**

1. Claessens M, Van Cauwenberghe L, Vandegehuchte MB *et al.* New techniques for the detection of microplastics in sediments and field collected organisms. *Mar Pollut Bull*. 2013;**70**:227-33 <https://doi.org/https://doi.org/10.1016/j.marpolbul.2013.03.009>

2. Nava V, Frezzotti ML, Leoni B. Raman spectroscopy for the analysis of microplastics in aquatic systems. *Appl Spectrosc*. 2021;**75**:1341-57 <https://doi.org/10.1177/00037028211043119>

3. China MoEaEotPsRo Water quality-determination of dissolved oxygen-electrochemical probe method

4. China MoEaEotPsRo Water quality- determination of total nitrogen - alkaline potassium persulfate digestion uv spectrophotometric method.

5. China MoEaEotPsRo Water quality-determination of total phosphorus-ammonium molybdate spectrophotometric method.

6. Wang H, Zhu J, He Y *et al.* Photoaging process and mechanism of four commonly commercial microplastics. *J Hazard Mater*. 2023;**451**:131151 <https://doi.org/https://doi.org/10.1016/j.jhazmat.2023.131151>

7. Huang Y, Ding J, Zhang G *et al.* Interactive effects of microplastics and selected pharmaceuticals on red tilapia: Role of microplastic aging. *Sci Total Environ*. 2021;**752**:142256 <https://doi.org/https://doi.org/10.1016/j.scitotenv.2020.142256>

8. Christensen GD, Simpson WA, Younger JJ *et al.* Adherence of coagulase-negative staphylococci to plastic tissue culture plates: A quantitative model for the adherence of staphylococci to medical devices. *J Clin Microbiol*. 1985;**22**:996-1006 <https://doi.org/10.1128/jcm.22.6.996-1006.1985>

9. Lowry O, Rosebrough N, Farr AL *et al.* Protein measurement with the phenol reagent. *J Biol Chem*. 1951;**193**:265-75 <https://doi.org/https://doi.org/10.1016/S0021-9258(19)52451-6>

10. DuBois M, Gilles KA, Hamilton JK *et al.* Colorimetric method for determination of sugars and related substances. *Anal Chem*. 1956;**28**:350-56 <https://doi.org/10.1021/ac60111a017>

11. Tu C, Chen T, Zhou Q *et al.* Biofilm formation and its influences on the properties of microplastics as affected by exposure time and depth in the seawater. *Sci Total Environ*. 2020;**734**:139237 <https://doi.org/https://doi.org/10.1016/j.scitotenv.2020.139237>

12. Callahan BJ, McMurdie PJ, Rosen MJ *et al.* Dada2: High-resolution sample inference from illumina amplicon data. *Nat Methods*. 2016;**13**:581-3 <https://doi.org/10.1038/nmeth.3869>

13. Bolyen E, Rideout JR, Dillon MR *et al.* Reproducible, interactive, scalable and extensible microbiome data science using qiime 2. *Nat Biotechnol*. 2019;**37**:852-57 <https://doi.org/10.1038/s41587-019-0209-9>

14. Quast C, Pruesse E, Yilmaz P *et al.* The silva ribosomal rna gene database project: Improved data processing and web-based tools. *Nucleic Acids Res*. 2013;**41**:D590-6 <https://doi.org/10.1093/nar/gks1219>

15. Bolger AM, Lohse M, Usadel B. Trimmomatic: A flexible trimmer for illumina sequence data. *Bioinformatics*. 2014;**30**:2114-20 <https://doi.org/10.1093/bioinformatics/btu170>

16. Langmead B, Salzberg SL. Fast gapped-read alignment with bowtie 2. *Nat Methods*. 2012;**9**:357-9 <https://doi.org/10.1038/nmeth.1923>

17. Franzosa EA, McIver LJ, Rahnavard G *et al.* Species-level functional profiling of metagenomes and metatranscriptomes. *Nat Methods*. 2018;**15**:962-68 <https://doi.org/10.1038/s41592-018-0176-y>

18. Niu L, Chen Y, Li Y *et al.* Diversity, abundance and distribution characteristics of potential polyethylene and polypropylene microplastic degradation bacterial communities in the urban river. *Water Res*. 2023;**232**:119704 <https://doi.org/https://doi.org/10.1016/j.watres.2023.119704>

19. Li W, Miao L, Adyel TM *et al.* Characterization of dynamic plastisphere and their underlying effects on the aging of biodegradable and traditional plastics in freshwater ecosystems. *J Hazard Mater*. 2023;**446**:130714 <https://doi.org/https://doi.org/10.1016/j.jhazmat.2022.130714>

20. Ho W-K, Law JC-F, Zhang T *et al.* Effects of weathering on the sorption behavior and toxicity of polystyrene microplastics in multi-solute systems. *Water Res*. 2020;**187**:116419 <https://doi.org/https://doi.org/10.1016/j.watres.2020.116419>

21. Pedireddy S, Lee HK, Tjiu WW *et al.* One-step synthesis of zero-dimensional hollow nanoporous gold nanoparticles with enhanced methanol electrooxidation performance. *Nat Commun*. 2014;**5**:4947 <https://doi.org/10.1038/ncomms5947>

22. Zhang J-H, Li Z, Xu J *et al.* Versatile self-assembled electrospun micropyramid arrays for high-performance on-skin devices with minimal sensory interference. *Nat Commun*. 2022;**13**:5839 <https://doi.org/10.1038/s41467-022-33454-y>

23. Han H, Yan P, Li Q *et al.* Photothermal upcycling of waste polyvinyl chloride plastics. *Environ Sci Technol*. 2024;**58**:21861-70 <https://doi.org/10.1021/acs.est.4c07350>

24. Schrap SM, Sleijpen GLG, Seinen W *et al.* Sorption kinetics of chlorinated hydrophobic organic chemicals. *Environ Sci Pollut Res*. 1994;**1**:81-92 <https://doi.org/10.1007/BF02986511>

25. Wang Z, Ding J, Song X *et al.* Aging of poly (lactic acid)/poly (butylene adipate-co-terephthalate) blends under different conditions: Environmental concerns on biodegradable plastic. *Sci Total Environ*. 2023;**855**:158921 <https://doi.org/https://doi.org/10.1016/j.scitotenv.2022.158921>

26. González-Torres P, García-Ruiz Á, La Rubia MD. Degradation and migration in olive oil packaged in polyethylene terephthalate under thermal treatment and storage conditions. *Appl Sci*. 2024;**14**:7507

27. Rodrigues MO, Abrantes N, Gonçalves FJM *et al.* Spatial and temporal distribution of microplastics in water and sediments of a freshwater system (antuã river, portugal). *Sci Total Environ*. 2018;**633**:1549-59 <https://doi.org/https://doi.org/10.1016/j.scitotenv.2018.03.233>

28. Rajakumar K, Sarasvathy V, Thamarai Chelvan A *et al.* Natural weathering studies of polypropylene. *J Polym Environ*. 2009;**17**:191-202 <https://doi.org/10.1007/s10924-009-0138-7>

29. Campanale C, Savino I, Massarelli C *et al.* Fourier transform infrared spectroscopy to assess the degree of alteration of artificially aged and environmentally weathered microplastics. *Polymers*. 2023;**15**:911

30. Alavian Petroody SS, Hashemi SH, Škrlep L *et al.* UV light causes structural changes in microplastics exposed in bio-solids. *Polymers*. 2023;**15**:4322

31. Chelomin VP, Istomina AA, Mazur AA *et al.* New insights into the mechanisms of toxicity of aging microplastics. *Toxics*. 2024;**12** <https://doi.org/10.3390/toxics12100726>

32. Niu Z, Curto M, Le Gall M *et al.* Accelerated fragmentation of two thermoplastics (polylactic acid and polypropylene) into microplastics after uv radiation and seawater immersion. *Ecotoxicol Environ Saf*. 2024;**271**:115981 <https://doi.org/https://doi.org/10.1016/j.ecoenv.2024.115981>

33. Esakkimuthu ES, Ponnuchamy V, Mikuljan M *et al.* Fungal enzyme degradation of lignin-PLA composites: Insights from experiments and molecular docking simulations. *Heliyon*. 2024;**10** <https://doi.org/10.1016/j.heliyon.2023.e23838>

34. Catteau A, Le Guernic A, Palos Ladeiro M *et al.* Integrative biomarker response - threshold (IBR-T): Refinement of IBRv2 to consider the reference and threshold values of biomarkers. *J Environ Manage*. 2023;**341**:118049 <https://doi.org/https://doi.org/10.1016/j.jenvman.2023.118049>

35. Beliaeff B, Burgeot T. Integrated biomarker response: A useful tool for ecological risk assessment. *Environ Toxicol Chem*. 2002;**21**:1316-22 <https://doi.org/10.1002/etc.5620210629>

36. Toyofuku M, Nomura N, Eberl L. Types and origins of bacterial membrane vesicles. *Nat Rev Microbiol*. 2019;**17**:13-24 <https://doi.org/10.1038/s41579-018-0112-2>

37. Haber F, Weiss J, Pope WJ. The catalytic decomposition of hydrogen peroxide by iron salts. *Proceedings of the Royal Society of London Series A - Mathematical and Physical Sciences*. 1934;**147**:332-51 <https://doi.org/doi:10.1098/rspa.1934.0221>

38. Yim MB, Chock PB, Stadtman ER. Copper, zinc superoxide dismutase catalyzes hydroxyl radical production from hydrogen peroxide. *Proc Natl Acad Sci USA*. 1990;**87**:5006-10 <https://doi.org/doi:10.1073/pnas.87.13.5006>

39. Wang J, Zhang T, Shen X *et al.* Serum metabolomics for early diagnosis of esophageal squamous cell carcinoma by UHPLC-QTOF/MS. *Metabolomics*. 2016;**12**:116 <https://doi.org/10.1007/s11306-016-1050-5>

40. Thévenot EA, Roux A, Xu Y *et al.* Analysis of the human adult urinary metabolome variations with age, body mass index, and gender by implementing a comprehensive workflow for univariate and OPLS statistical analyses. *J Proteome Res*. 2015;**14**:3322-35 <https://doi.org/10.1021/acs.jproteome.5b00354>

41. Reubsaet L, Sweredoski MJ, Moradian A. Data-independent acquisition for the Orbitrap q exactive hf: A tutorial. *J Proteome Res*. 2019;**18**:803-13 <https://doi.org/10.1021/acs.jproteome.8b00845>

42. Cox J, Hein MY, Luber CA *et al.* Accurate proteome-wide label-free quantification by delayed normalization and maximal peptide ratio extraction, termed maxlfq. *Mol Cell Proteomics*. 2014;**13**:2513-26 <https://doi.org/10.1074/mcp.M113.031591>

43. Unwin RD, Griffiths JR, Whetton AD. Simultaneous analysis of relative protein expression levels across multiple samples using itraq isobaric tags with 2D nano LC-MS/MS. *Nat Protoc*. 2010;**5**:1574-82 <https://doi.org/10.1038/nprot.2010.123>

44. Tatusov RL, Fedorova ND, Jackson JD *et al.* The cog database: An updated version includes eukaryotes. *BMC Bioinformatics*. 2003;**4**:41 <https://doi.org/10.1186/1471-2105-4-41>

45. Machacova K, Vainio E, Urban O *et al.* Seasonal dynamics of stem N_2_O exchange follow the physiological activity of boreal trees. *Nat Commun*. 2019;**10**:4989 <https://doi.org/10.1038/s41467-019-12976-y>

46. Song YK, Hong SH, Jang M *et al.* Combined effects of uv exposure duration and mechanical abrasion on microplastic fragmentation by polymer type. *Environ Sci Technol*. 2017;**51**:4368-76 <https://doi.org/10.1021/acs.est.6b06155>

47. Yu K, Ni J, Zhou H *et al.* Effects of in-situ crystallization on poly (lactic acid) microcellular foaming: Density functional theory and experiment. *Polymer*. 2020;**200**:122539 <https://doi.org/https://doi.org/10.1016/j.polymer.2020.122539>

48. Chauhan A, Jindal T Microbiological culture media: Types, role and composition. In: Chauhan A, Jindal T (eds.). *Microbiological methods for environment, food and pharmaceutical analysis*, Cham: Springer International Publishing. 23-66. Retreived from <https://doi.org/10.1007/978-3-030-52024-3_3>

49. Das G, Bordoloi NK, Rai SK *et al.* Biodegradable and biocompatible epoxidized vegetable oil modified thermostable poly(vinyl chloride): Thermal and performance characteristics post biodegradation with *Pseudomonas Aeruginosa* and *Achromobacter* sp. *J Hazard Mater*. 2012;**209-210**:434-42 <https://doi.org/https://doi.org/10.1016/j.jhazmat.2012.01.043>

50. Wang Y, Inagawa Y, Saito T *et al.* Enzymatic hydrolysis of bacterial poly(3-hydroxybutyrate-co-3-hydroxypropionate)s by poly(3-hydroxyalkanoate) depolymerase from *Acidovorax* sp. Tp4. *Biomacromolecules*. 2002;**3**:828-34 <https://doi.org/10.1021/bm020019p>

51. Kobayashi T, Sugiyama A, Kawase Y *et al.* Biochemical and genetic characterization of an extracellular poly(3-hydroxybutyrate) depolymerase from *Acidovorax* sp. Strain tp4. *J Environ Polym Degrad*. 1999;**7**:9-18 <https://doi.org/10.1023/A:1021885901119>

52. Dwyer DF, Tiedje JM. Metabolism of polyethylene glycol by two anaerobic bacteria, *Desulfovibrio Desulfuricans* and a *Bacteroides* sp. *Appl Environ Microbiol*. 1986;**52**:852-6 <https://doi.org/10.1128/aem.52.4.852-856.1986>

53. Martínez V, de la Peña F, García-Hidalgo J *et al.* Identification and biochemical evidence of a medium-chain-length polyhydroxyalkanoate depolymerase in the *Bdellovibrio Bacteriovorus* predatory hydrolytic arsenal. *Appl Environ Microbiol*. 2012;**78**:6017-26 <https://doi.org/10.1128/AEM.01099-12>

54. Sekhar VC, Nampoothiri KM, Mohan AJ *et al.* Microbial degradation of high impact polystyrene (HIPS), an e-plastic with decabromodiphenyl oxide and antimony trioxide. *J Hazard Mater*. 2016;**318**:347-54 <https://doi.org/https://doi.org/10.1016/j.jhazmat.2016.07.008>

55. Sudhakar M, Priyadarshini C, Doble M *et al.* Marine bacteria mediated degradation of nylon 66 and 6. *Int Biodeterior Biodegrad*. 2007;**60**:144-51 <https://doi.org/https://doi.org/10.1016/j.ibiod.2007.02.002>

56. Morohoshi T, Oi T, Aiso H *et al.* Biofilm formation and degradation of commercially available biodegradable plastic films by bacterial consortiums in freshwater environments. *Microbes Environ*. 2018;**33**:332-35 <https://doi.org/10.1264/jsme2.ME18033>

57. Montazer Z, Habibi-Najafi MB, Mohebbi M *et al.* Microbial degradation of UV-pretreated low-density polyethylene films by novel polyethylene-degrading bacteria isolated from plastic-dump soil. *J Polym Environ*. 2018;**26**:3613-25 <https://doi.org/10.1007/s10924-018-1245-0>

58. Koutny M, Amato P, Muchova M *et al.* Soil bacterial strains able to grow on the surface of oxidized polyethylene film containing prooxidant additives. *Int Biodeterior Biodegrad*. 2009;**63**:354-57 <https://doi.org/https://doi.org/10.1016/j.ibiod.2008.11.003>

59. Khandare SD, Chaudhary DR, Jha B. Marine bacterial biodegradation of low-density polyethylene (LDPE) plastic. *Biodegradation*. 2021;**32**:127-43 <https://doi.org/10.1007/s10532-021-09927-0>

60. Yoshida S, Hiraga K, Takehana T *et al.* A bacterium that degrades and assimilates poly(ethylene terephthalate). *Science*. 2016;**351**:1196-99 <https://doi.org/10.1126/science.aad6359>

61. Austin HP, Allen MD, Donohoe BS *et al.* Characterization and engineering of a plastic-degrading aromatic polyesterase. *Proc Natl Acad Sci USA*. 2018;**115**:E4350-E57 <https://doi.org/10.1073/pnas.1718804115>

62. Nakajima-Kambe T, Toyoshima K, Saito C *et al.* Rapid monomerization of poly(butylene succinate)-co-(butylene adipate) by *Leptothrix* sp. *J Biosci Bioeng*. 2009;**108**:513-16 <https://doi.org/https://doi.org/10.1016/j.jbiosc.2009.05.018>

63. Nakajima-Kambe T, Ichihashi F, Matsuzoe R *et al.* Degradation of aliphatic-aromatic copolyesters by bacteria that can degrade aliphatic polyesters. *Polym Degrad Stab*. 2009;**94**:1901-05 <https://doi.org/https://doi.org/10.1016/j.polymdegradstab.2009.08.006>

64. Suyama T, Tokiwa Y, Ouichanpagdee P *et al.* Phylogenetic affiliation of soil bacteria that degrade aliphatic polyesters available commercially as biodegradable plastics. *Appl Environ Microbiol*. 1998;**64**:5008-11 <https://doi.org/10.1128/aem.64.12.5008-5011.1998>

65. Schirmer A, Jendrossek D. Molecular characterization of the extracellular poly(3-hydroxyoctanoic acid) [P(3HO)] depolymerase gene of *Pseudomonas Fluorescens* gk13 and of its gene product. *J Bacteriol*. 1994;**176**:7065-73 <https://doi.org/10.1128/jb.176.22.7065-7073.1994>

66. Nadeem H, Alia KB, Muneer F *et al.* Isolation and identification of low-density polyethylene degrading novel bacterial strains. *Arch Microbiol*. 2021;**203**:5417-23 <https://doi.org/10.1007/s00203-021-02521-1>

67. Kato C, Honma A, Sato S *et al.* Poly 3-hydroxybutyrate- co -3-hydroxyhexanoate films can be degraded by the deep-sea microbes at high pressure and low temperature conditions. *High Press Res*. 2019;**39**:1-10 <https://doi.org/10.1080/08957959.2019.1584196>

68. Ahmad A, Tsutsui A, Iijima S *et al.* Gene structure and comparative study of two different plastic-degrading esterases from roseateles depolymerans strain tb-87. *Polym Degrad Stab*. 2019;**164**:109-17 <https://doi.org/https://doi.org/10.1016/j.polymdegradstab.2019.04.003>

69. Suzuki M, Tachibana Y, Kazahaya J-i *et al.* Difference in environmental degradability between poly(ethylene succinate) and poly(3-hydroxybutyrate). *J Polym Res*. 2017;**24**:217 <https://doi.org/10.1007/s10965-017-1383-4>

70. Sekiguchi T, Sato T, Enoki M *et al.* Isolation and characterization of biodegradable plastic degrading bacteria from deep-sea environments. *JAMSTEC Rep Res Dev*. 2011;**11**:33-41 <https://doi.org/10.5918/jamstecr.11.33>

71. Hatanaka T, Asahi N, Tsuji M. Purification and characterization of poly(vinyl alcohol) dehydrogenase from *Pseudomonas* sp. 113p3. *Biosci Biotechnol Biochem*. 1995;**59**:1813-16 <https://doi.org/10.1271/bbb.59.1813>

72. Kathiresan K. Polythene and plastics-degrading microbes from the mangrove soil. *Rev Biol Trop*. 2003;**51**:629-33

73. Hussein AA, Alzuhairi M, Aljanabi NH. Degradation and depolymerization of plastic waste by local bacterial isolates and bubble column reactor. *AIP Conf Proc*. 2018;**1968** <https://doi.org/10.1063/1.5039268>

74. Kadouri D, Jurkevitch E, Okon Y. Poly β-hydroxybutyrate depolymerase (PhaZ) in *Azospirillum Brasilense* and characterization of a phaz mutant. *Arch Microbiol*. 2003;**180**:309-18 <https://doi.org/10.1007/s00203-003-0590-z>

75. Boyandin AN, Prudnikova SV, Karpov VA *et al.* Microbial degradation of polyhydroxyalkanoates in tropical soils. *Int Biodeterior Biodegrad*. 2013;**83**:77-84 <https://doi.org/https://doi.org/10.1016/j.ibiod.2013.04.014>

76. Lu J, Takahashi A, Ueda S. 3-hydroxybutyrate oligomer hydrolase and 3-hydroxybutyrate dehydrogenase participate in intracellular polyhydroxybutyrate and polyhydroxyvalerate degradation in paracoccus denitrificans. *Appl Environ Microbiol*. 2014;**80**:986-93 <https://doi.org/doi:10.1128/AEM.03396-13>

77. Ohta T, Tani A, Kimbara K *et al.* A novel nicotinoprotein aldehyde dehydrogenase involved in polyethylene glycol degradation. *Appl Microbiol Biotechnol*. 2005;**68**:639-46 <https://doi.org/10.1007/s00253-005-1936-z>

78. Mergaert J, Ruffieux K, Bourban C *et al.* In vitro biodegradation of polyester-based plastic materials by selected bacterial cultures. *J Polym Environ*. 2000;**8**:17-27 <https://doi.org/10.1023/A:1010168011209>

79. Kasuya K, Inoue Y, Tanaka T *et al.* Biochemical and molecular characterization of the polyhydroxybutyrate depolymerase of *Comamonas Acidovorans* ym1609, isolated from freshwater. *Appl Environ Microbiol*. 1997;**63**:4844-52 <https://doi.org/10.1128/aem.63.12.4844-4852.1997>

80. Awasthi S, Srivastava P, Singh P *et al.* Biodegradation of thermally treated high-density polyethylene (HDPE) by *Klebsiella Pneumoniae* ch001. *3 Biotech*. 2017;**7**:332 <https://doi.org/10.1007/s13205-017-0959-3>

81. Sangeetha Devi R, Ramya R, Kannan K *et al.* Investigation of biodegradation potentials of high density polyethylene degrading marine bacteria isolated from the coastal regions of tamil nadu, india. *Mar Pollut Bull*. 2019;**138**:549-60 <https://doi.org/https://doi.org/10.1016/j.marpolbul.2018.12.001>

82. Habib S, Iruthayam A, Abd Shukor MY *et al.* Biodeterioration of untreated polypropylene microplastic particles by antarctic bacteria. *Polymers*. 2020;**12**:2616

83. Amadi LO, Nosayame TO Biodegradation of polypropylene by bacterial isolates from the organs of a fish, *liza grandisquamis* harvested from ohiakwu estuary in rivers state, nigeria.

84. Jeon J-M, Park S-J, Choi T-R *et al.* Biodegradation of polyethylene and polypropylene by *Lysinibacillus* species jjy0216 isolated from soil grove. *Polym Degrad Stab*. 2021;**191**:109662 <https://doi.org/https://doi.org/10.1016/j.polymdegradstab.2021.109662>

85. Helen A, Uche EC, Hamid FS. Screening for polypropylene degradation potential of bacteria isolated from mangrove ecosystems in peninsular malaysia. *Int J Biosci Biochem Bioinform*. 2017;**7**:245-51

86. Auta HS, Emenike CU, Jayanthi B *et al.* Growth kinetics and biodeterioration of polypropylene microplastics by *Bacillus* sp. and *Rhodococcus* sp. Isolated from mangrove sediment. *Mar Pollut Bull*. 2018;**127**:15-21 <https://doi.org/https://doi.org/10.1016/j.marpolbul.2017.11.036>

87. Srivastava A, Oliya P, Singh S *et al.* Polypropylene degradation potential of microbes isolated from dumping site. *Pollut Res*. 2020;**39**:282-91

88. Nanthini Devi K, Raju P, Santhanam P *et al.* Biodegradation of low-density polyethylene and polypropylene by microbes isolated from vaigai river, madurai, india. *Arch Microbiol*. 2021;**203**:6253-65 <https://doi.org/10.1007/s00203-021-02592-0>

89. Skariyachan S, Patil AA, Shankar A *et al.* Enhanced polymer degradation of polyethylene and polypropylene by novel thermophilic consortia of *Brevibacillus* sps. And aneurinibacillus sp. Screened from waste management landfills and sewage treatment plants. *Polym Degrad Stab*. 2018;**149**:52-68 <https://doi.org/https://doi.org/10.1016/j.polymdegradstab.2018.01.018>

90. Lv S, Wang Q, Li Y *et al.* Biodegradation of polystyrene (PS) and polypropylene (PP) by deep-sea psychrophilic bacteria of *Pseudoalteromonas* in accompany with simultaneous release of microplastics and nanoplastics. *Sci Total Environ*. 2024;**948**:174857 <https://doi.org/https://doi.org/10.1016/j.scitotenv.2024.174857>

91. Jeyavani J, Al-Ghanim KA, Govindarajan M *et al.* Bacterial screening in indian coastal regions for efficient polypropylene microplastics biodegradation. *Sci Total Environ*. 2024;**918**:170499 <https://doi.org/https://doi.org/10.1016/j.scitotenv.2024.170499>

92. Wang Y, Hu T, Zhang W *et al.* Biodegradation of polylactic acid by a mesophilic bacteria *Bacillus Safensis*. *Chemosphere*. 2023;**318**:137991 <https://doi.org/https://doi.org/10.1016/j.chemosphere.2023.137991>

93. Tomita K, Kuroki Y, Nagai K. Isolation of thermophiles degrading poly(l-lactic acid). *J Biosci Bioeng*. 1999;**87**:752-55 <https://doi.org/https://doi.org/10.1016/S1389-1723(99)80148-0>

94. Tomita K, Tsuji H, Nakajima T *et al.* Degradation of poly(d-lactic acid) by a thermophile. *Polym Degrad Stab*. 2003;**81**:167-71 <https://doi.org/https://doi.org/10.1016/S0141-3910(03)00086-7>

95. Arena M, Abbate C, Fukushima K *et al.* Degradation of poly (lactic acid) and nanocomposites by bacillus licheniformis. *Environ Sci Pollut Res*. 2011;**18**:865-70 <https://doi.org/10.1007/s11356-011-0443-2>

96. Tomita K, Nakajima T, Kikuchi Y *et al.* Degradation of poly(l-lactic acid) by a newly isolated thermophile. *Polym Degrad Stab*. 2004;**84**:433-38 <https://doi.org/https://doi.org/10.1016/j.polymdegradstab.2003.12.006>

97. Wang Z, Wang Y, Guo Z *et al.* Purification and characterization of poly(l-lactic acid) depolymerase from pseudomonas sp strain ds04-t. *Polym Eng Sci*. 2011;**51**:454-59 <https://doi.org/10.1002/pen.21857>

98. Jeon HJ, Kim MN. Biodegradation of poly(l-lactide) (PLA) exposed to uv irradiation by a mesophilic bacterium. *Int Biodeterior Biodegrad*. 2013;**85**:289-93 <https://doi.org/https://doi.org/10.1016/j.ibiod.2013.08.013>

99. Yao Z, Seong HJ, Jang Y-S. Degradation of low density polyethylene by *Bacillus* species. *Applied Biological Chemistry*. 2022;**65**:84 <https://doi.org/10.1186/s13765-022-00753-3>

100. Herrera DAG, Mojicevic M, Pantelic B *et al.* Exploring microorganisms from plastic-polluted sites: Unveiling plastic degradation and pha production potential. *Microorganisms*. 2023;**11**:2914

101. Dhaka V, Singh S, Ramamurthy PC *et al.* Biological degradation of polyethylene terephthalate by rhizobacteria. *Environ Sci Pollut Res*. 2023;**30**:116488-97 <https://doi.org/10.1007/s11356-022-20324-9>

102. Uscátegui YL, Arévalo FR, Díaz LE *et al.* Microbial degradation, cytotoxicity and antibacterial activity of polyurethanes based on modified castor oil and polycaprolactone. *J Biomater Sci Polym Ed*. 2016;**27**:1860-79 <https://doi.org/10.1080/09205063.2016.1239948>

103. Sharma KK, Panwar H, Gupta KK. Isolation and characterization of bio-prospecting gut strains *Bacillus Safensis* cgk192 and *Bacillus Australimaris* cgk221 for plastic (hdpe) degradation. *Biotechnol Lett*. 2024;**46**:671-89 <https://doi.org/10.1007/s10529-024-03486-z>

104. Dang TCH, Nguyen DT, Thai H *et al.* Plastic degradation by thermophilic *Bacillus* sp. Bcbt21 isolated from composting agricultural residual in vietnam. *Adv Nat Sci: Nanosci Nanotechnol*. 2018;**9**:015014 <https://doi.org/10.1088/2043-6254/aaabaf>

105. Wang N, Guan F, Lv X *et al.* Enhancing secretion of polyethylene terephthalate hydrolase petase in bacillus subtilis WB600 mediated by the spamy signal peptide. *Lett Appl Microbiol*. 2020;**71**:235-41 <https://doi.org/10.1111/lam.13312>

106. Dąbrowska GB, Janczak K, Richert A. Combined use of bacillus strains and miscanthus for accelerating biodegradation of poly(lactic acid) and poly(ethylene terephthalate). *PeerJ*. 2021;**9**:e10957 <https://doi.org/10.7717/peerj.10957>

107. Maheswaran B, Al-Ansari M, Al-Humaid L *et al.* In vivo degradation of polyethylene terephthalate using microbial isolates from plastic polluted environment. *Chemosphere*. 2023;**310**:136757 <https://doi.org/https://doi.org/10.1016/j.chemosphere.2022.136757>

108. Giacomucci L, Raddadi N, Soccio M *et al.* Polyvinyl chloride biodegradation by pseudomonas citronellolis and bacillus flexus. *New Biotechnology*. 2019;**52**:35-41 <https://doi.org/https://doi.org/10.1016/j.nbt.2019.04.005>
